# Functional dissection of conserved RNAi pathways in antiviral immunity of several hemipteran insects

**DOI:** 10.1371/journal.ppat.1014753

**Published:** 2026-10-30

**Authors:** Yan Zhang, Zhuang-Xin Ye, Qing-Lu Ma, Bi-Yun Wang, Ji-Chong Zhuo, Hai-Jian Huang, Chuan-Xi Zhang, Jian-Ping Chen, Jun-Min Li

**Affiliations:** State Key Laboratory for Quality and Safety of Agro-Products, Key Laboratory of Biotechnology in Plant Protection of MARA, Zhejiang Key Laboratory of Green Plant Protection, Institute of Plant Virology, Ningbo University, Ningbo, China; Wageningen Bioveterinary Research, NETHERLANDS, KINGDOM OF THE

## Abstract

The small silencing RNA pathways are crucial post-transcriptional gene silencing mechanisms involved in antiviral defense, gene regulation, and transposon suppression. In insects, the three main pathways are the microRNA (miRNA), Piwi-interacting RNA (piRNA), and small interfering RNA (siRNA) pathways. While these pathways are well-studied in model insects like fruit flies and mosquitoes (Order: Diptera), their functions in non-model insects remain largely unexplored. In this study, we conducted a comprehensive functional screening of core RNAi-related genes in a hemipteran insect, the small brown planthopper (*Laodelphax striatellus*). Our results demonstrated that genes in the miRNA pathway (*AGO1* and *DCR1*) and the piRNA pathway (*Piwi* and *AGO3*) are essential for the survival, development, and fecundity of *L. striatellus*, but they are not actively involved in the antiviral response. Unexpectedly, knocking down the key siRNA pathway gene *DCR2* did not produce a detectable change in viral accumulation or the abundance of virus-derived small interfering RNAs. This finding suggests that substantial depletion of *DCR2* does not detectably impair the measured antiviral siRNA responses in *L. striatellus* under our experimental conditions. A similar lack of detectable antiviral phenotypes following *DCR2* knockdown was observed in three other representative hemipteran insects from different families/genera (*Sogatella furcifera*, *Nilaparvata lugens*, and *Bemisia tabaci*). Additionally, CRISPR/Cas9-mediated knockout of DCR2 resulted in embryonic lethality in homozygous mutant strains of *L. striatellus*, while heterozygous mutants showed significantly prolonged developmental duration. Functional RNAi-of-RNAi assays further demonstrated that *DCR2* pre-knockdown did not detectably reduce silencing of a subsequently introduced dsRNA reporter in *L. striatellus*, and DCR2-heterozygous mutants with reduced DCR2 expression showed no change in viral accumulation. These results reveal an essential developmental role for DCR2 and indicate that partial DCR2 depletion does not measurably compromise antiviral immunity under the tested conditions. Collectively, our findings broaden our understanding of the organization and biological functions of small silencing RNA pathways in hemipteran insects.

## Author summary

In insects, the three major small silencing RNA pathways - the microRNA, Piwi-interacting RNA, and small interfering RNA (siRNA) pathways - play distinct yet interconnected roles in host biology, with the siRNA pathway serving as the primary antiviral defense. Compared to their well-documented roles in model dipteran insects, the functions of these pathways in non-model insects remain to be fully elucidated. In this study, we performed a comprehensive functional screen of core small silencing RNA-related genes in the hemipteran insect *Laodelphax striatellus* and demonstrated that depletion of AGO2, but not DCR2, consistently increased viral accumulation in the hemipteran species examined. Strikingly, CRISPR/Cas9-mediated knockout of DCR2 caused embryonic lethality in homozygous mutants of *L. striatellus*, suggesting that DCR2 is essential for embryonic viability in *L. striatellus*. RNAi-of-RNAi assays and analyses of DCR2 heterozygous mutants likewise did not reveal detectable impairment of the measured RNAi or antiviral phenotypes in this species. These findings broaden our understanding of RNAi-mediated antiviral immunity and developmental regulation in non-model insects.

## Introduction

RNA interference (RNAi) is a sequence-specific gene silencing mechanism found in plants, fungi and insects, playing vital roles in defense against RNA virus infection [1,2]. There are three main small silencing RNA pathways in insects, including the microRNA (miRNA) pathway, the Piwi-interacting RNA (piRNA) pathway, and the small interfering RNA (siRNA) pathway according to different types of small RNAs (sRNAs). These three small silencing RNA pathways play distinct but interconnected roles in insect biology, with siRNA mainly for antiviral defense, miRNA for gene regulation, and piRNA for transposon silencing [3]. miRNAs are a class of endogenous sRNAs with a length of about 22 nucleotides (nt), which play various essential regulatory roles in gene expression via degradation or translational repression of mRNAs [4,5]. The piRNA pathway is crucial for protecting the genome against the activity of transposable elements in animals, generating a class of piRNA of the size 25–30 nt, which regulates mRNA stability, protein synthesis, chromatin organization and genome structure by binding with PIWI clade of Argonaute proteins [6,7]. siRNAs are approximately 20–25 nt small RNAs derived from double-stranded RNA that mediate gene silencing by guiding the degradation of complementary single-stranded transcripts [8,9].

Previous studies revealed that the miRNA pathway plays a vital role in insect development [10–13], while piRNAs mainly function in germ cells [14–18]. In Hemiptera insects, silencing the miRNA pathway core genes (*AGO1* or *DCR1*) reduced miRNA levels followed by reduced fecundity and survival of *Bemisia tabaci*, *Sogatella furcifera*, *Nilaparvata lugens* and *Aphis gossypii* [13,19–21]. It has been revealed that piRNA pathway mediates antiviral defense in mosquitoes but not in fruit flies although both of them are Dipteran insects. Knockdown of *Piwi4* has been shown to enhance the replication of semliki forest virus in *Aedes aegypti* mosquito cells (Aag2) [16]. *Piwi5* and *AGO3* were demonstrated to be essential for piRNA biogenesis from sindbis virus (SINV) in infected Aag2 cells [17]. In contrast to *A. aegypti*, the piRNA pathway is not required for antiviral defense in *Drosophila melanogaster* [18]. Additionally, endogenous viral elements have been reported to be widespread in arthropod genomes and can also produce piRNAs [22]. In the small brown planthopper (*Laodelphax striatellus*), endogenous toti-like viral elements (LsETLVEs) are ubiquitously integrated into the host genome and produce piRNAs ranging from 24 to 29 nt in length [23].

Although the siRNA pathway is widely regarded as the primary antiviral immune mechanism in insects, most studies have been conducted in model Dipteran insects, particularly fruit flies [24–29] and mosquitoes [30–33]. During virus infection, Dicer-2 (DCR2) recognizes virus-derived dsRNA and cleaves it into siRNA duplexes. The siRNAs are then integrated into the RNA-induced silencing complex (RISC), where Argonaute-2 (AGO2) binds to the guide strand and mediates cleavage of the target RNA, while the passenger strand is discarded and degraded by a nuclease C3PO [34]. The DCR2-R2D2 heterodimer plays critical roles in both siRNA production and the loading of siRNAs onto AGO2. R2D2 contains two dsRNA-binding domains (RBD1 and RBD2). In *Drosophila*, dsRNA binds to the helicase domain of DCR2, whereas siRNA binds to R2D2 and is subsequently loaded onto AGO2 with the aid of the Hsc70/Hsp90 chaperone machinery [35]. Mutant flies lacking *AGO2* were more susceptible to viral infections with *Drosophila* C virus and cricket paralysis virus (CrPV). Moreover, *Dicer2* mutant flies were hypersensitive to CrPV infection [36,37]. R2D2 forms a heterodimer with DCR2 *in vivo*, which is required for siRNA loading into RISC, and *R2D2* mutant flies also exhibit enhanced disease susceptibility to CrPV [37]. In mosquitoes, viral titers and infection rates of SINV and dengue virus were significantly altered in *AGO2* and *DCR2* deficient insects [31,38,39]. Beyond flies and mosquitoes, siRNA-mediated antiviral immunity has also been systematically studied in non-model arthropods, such as sand flies [40], Asian longhorned ticks [41,42], and shrimp [43].

Virus-derived small interfering RNAs (vsiRNAs) are key components of the siRNA-based antiviral response in insects. In Dipteran insects, vsiRNAs typically exhibit a 21-nt peak and are generated through DCR2-mediated cleavage [44]. Interestingly, previous studies have reported variations in vsiRNA peak lengths across different insect orders [45], implying potential differences in their production mechanisms mediated by siRNA pathway of various host insects. For example, vsiRNA peak is at 20-nt in Lepidopteran insects [46], 22-nt in Hymenopteran insects [47], and both 21- and 22-nt in Hemipteran insects [48,49]. Additionally, DCR2 has also been found to inhibit molting and regulate wing expansion in *S. furcifera* [50], indicating possible functional diversity of DCR2 besides mediating antiviral RNAi in insects.

Our current understanding of insect small silencing RNA pathways primarily relies on studies of mosquitoes and flies, while mechanisms of small silencing RNA pathways in non-model insects, particularly during viral infection, remain poorly understood. The three planthopper species *L. striatellus*, *N. lugens*, and *S. furcifera* (family Delphacidae, order Hemiptera) are among the most destructive insect pests in rice ﬁelds. In addition to transmitting the economically important rice viruses (such as rice stripe virus, RSV), our recent investigations reveal that *L. striatellus* also harbors a diverse array of symbiotic viruses or insect-specific RNA viruses [49]. To elucidate roles of RNAi in hemipteran insects, this study systematically investigates the pivotal functions of key small silencing RNA pathways genes in *L. striatellus*. Notably, we found that AGO2 depletion consistently enhanced viral accumulation, whereas DCR2 depletion did not produce a detectable antiviral phenotype under the conditions examined. Similar contrasting knockdown phenotypes for AGO2 and DCR2 were observed in three other hemipteran insects (*N. lugens*, *S. furcifera*, and *B. tabaci*). Importantly, CRISPR/Cas9-mediated DCR2 knockout in *L. striatellus* yielded homozygous mutant embryos that failed to hatch, indicating an essential role for DCR2 during embryonic development in *L. striatellus*, broadening our understanding of siRNA-mediated antiviral immunity in non-model insects.

## Results

### Identification of key small silencing RNA pathway genes in the three rice planthoppers

Based on the homologous search to the genes in model insects (*D. melanogaster* and *A. aegypti*), 21 core small silencing RNA pathway genes (*AGO1*, *DCR1*, *Piwi*, *AGO3*, *AGO2*, *DCR2* and *R2D2*) were identified in three rice planthoppers, among which 11 genes were newly identified, including *Ls_DCR1* (accession no. OP778735), *Ls_Piwi* (accession no. OP778736), *Ls_AGO3* (accession no. OP778737), *Ls_R2D2* (accession no. OP778738) of *L. striatellus* and *Sf_AGO1* (accession no. OP778739), *Sf_DCR1* (accession no. OP778741), *Sf_Piwi* (accession no. OP778744), *Sf_AGO3* (accession no. OP778745), *Sf_AGO2* (accession no. OP778740), *Sf_DCR2* (accession no. OP778742), and *Sf_R2D2* (accession no. OP778743) of *S. furcifera*. Phylogenetic analysis based on protein sequences of the small silencing RNA pathway core genes in *L. striatellus* revealed that most genes (except R2D2) clustered closely with those of *S. furcifera*. Together with *N. lugens*, they formed distinct clades separate from those of other species (Fig 1), consistent with the taxonomic relationships among the three species [51]. Domain analysis suggested that most genes of the three rice planthoppers possess conserved domains of the three small silencing RNA pathways core genes similar to other insects (Fig 1). It is worth noting that Ls_DCR1 lacks the DEAD/DEAH helicase_C (DHC) domain (Fig 1A), Ls_R2D2 has an additional dsRNA binding (dRB) motif (Fig 1B) compared to their counterparts in other insects, suggesting potential functional differences of these genes.

**Fig 1:**
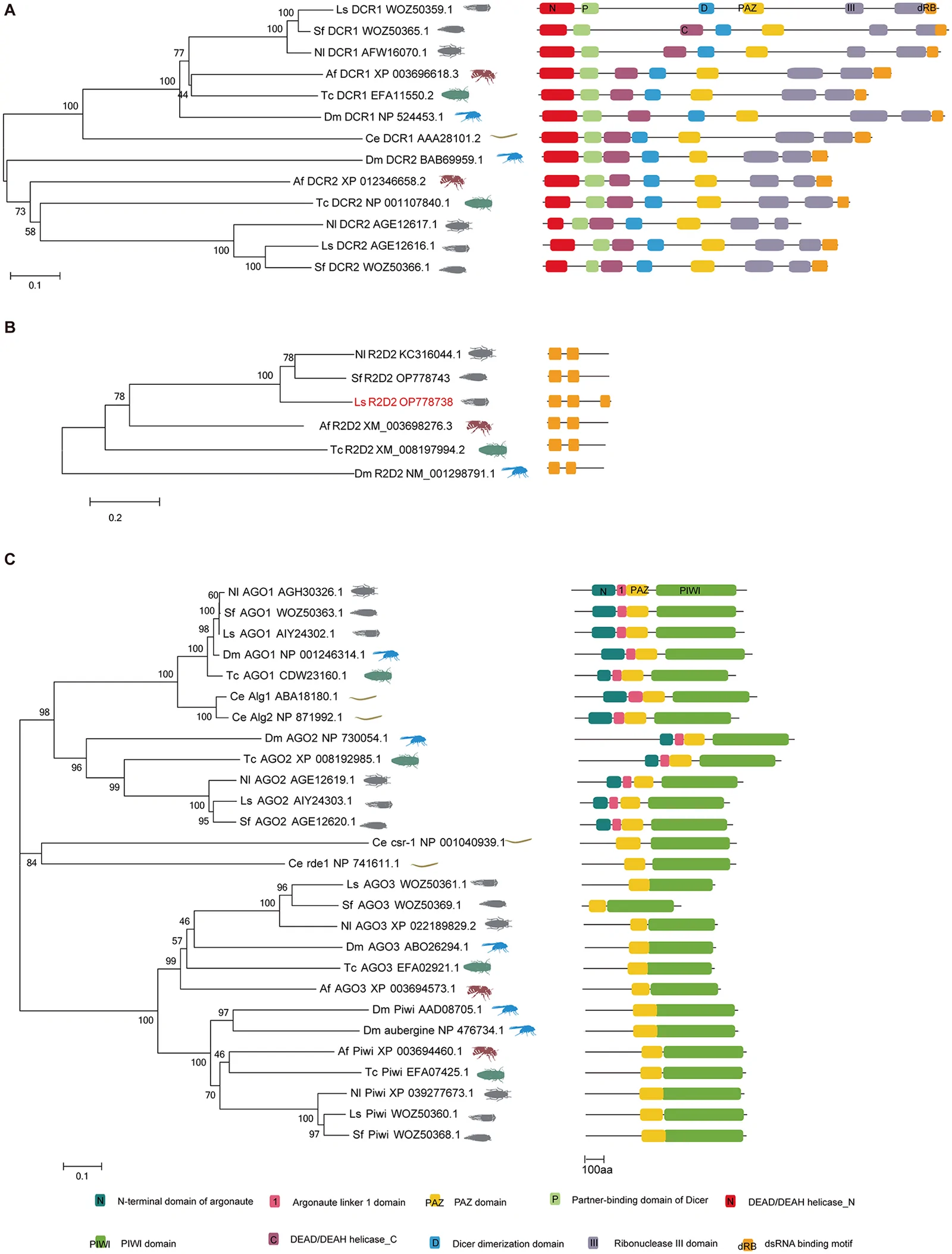
Phylogenetic and schematic domain architecture of the three small silencing RNA pathways key genes of *Laodelphax striatellus.* Phylogenetic trees were constructed using predicted amino acid sequences. **(A)** DCR1 and DCR2. **(B)** R2D2. **(C)** AGO1, AGO2, AGO3 and Piwi. Insect species abbreviations: Ls: *L. striatellus*; Nl: *Nilaparvata lugens*; Sf: *Sogatella furcifera*; Tc: *Tribolium castaneum*; Af: *Apis flor*ea; Dm: *Drosophila melanogaster*; Ce: *Caenorhabditis elegans*.

### Effects of key small silencing RNA pathway genes on host phenotypes of *L. striatellus*

To investigate the roles of the three small silencing RNA pathways on host biology, we performed RNAi-mediated knockdown of key small silencing RNA-related genes in *L. striatellus* and evaluated their effects on host fecundity, survival rate, and developmental duration. For the miRNA pathway, both *Ls_AGO1* and *Ls_DCR1* were expressed across the detected tissues of *L. striatellus*, with *Ls_AGO1* showing the lowest expression in the testis and *Ls_DCR1* showing the highest expression in both reproductive organs (ovary and testis) (Fig 2A-a). Relatively high expression of *Ls_AGO1* was observed in eggs and early nymphal stages (S1A Fig), while extremely high expression of *Ls_DCR1* was only detected in early stage of egg development (12h) (S1B Fig). After knockdown of *Ls_AGO1* and *Ls_DCR1* in third-instar nymphs (silencing efficiency shown in S2A Fig), the survival rate of *L. striatellus* was significantly reduced 12 days post-injection compared to the ds-*GFP* control group (Fig 2A-b). Additionally, treated nymphs exhibited molting defects, whereas most control nymphs successfully molted into adults. The defect was manifested by nymphs remaining in their original molting stage, with the old exoskeleton failing to shed (Fig 2A-b). Female fecundity was significantly reduced in both ds-*Ls_AGO1* and ds-*Ls_DCR1* treated insect groups compared to the ds-*GFP* control. Knockdown of *Ls_DCR1* completely abolished egg production, while *Ls_AGO1* knockdown led to a limited number of eggs, all of which failed to hatch (Fig 2A-c; S3 Fig; S1 Table).

**Fig 2:**
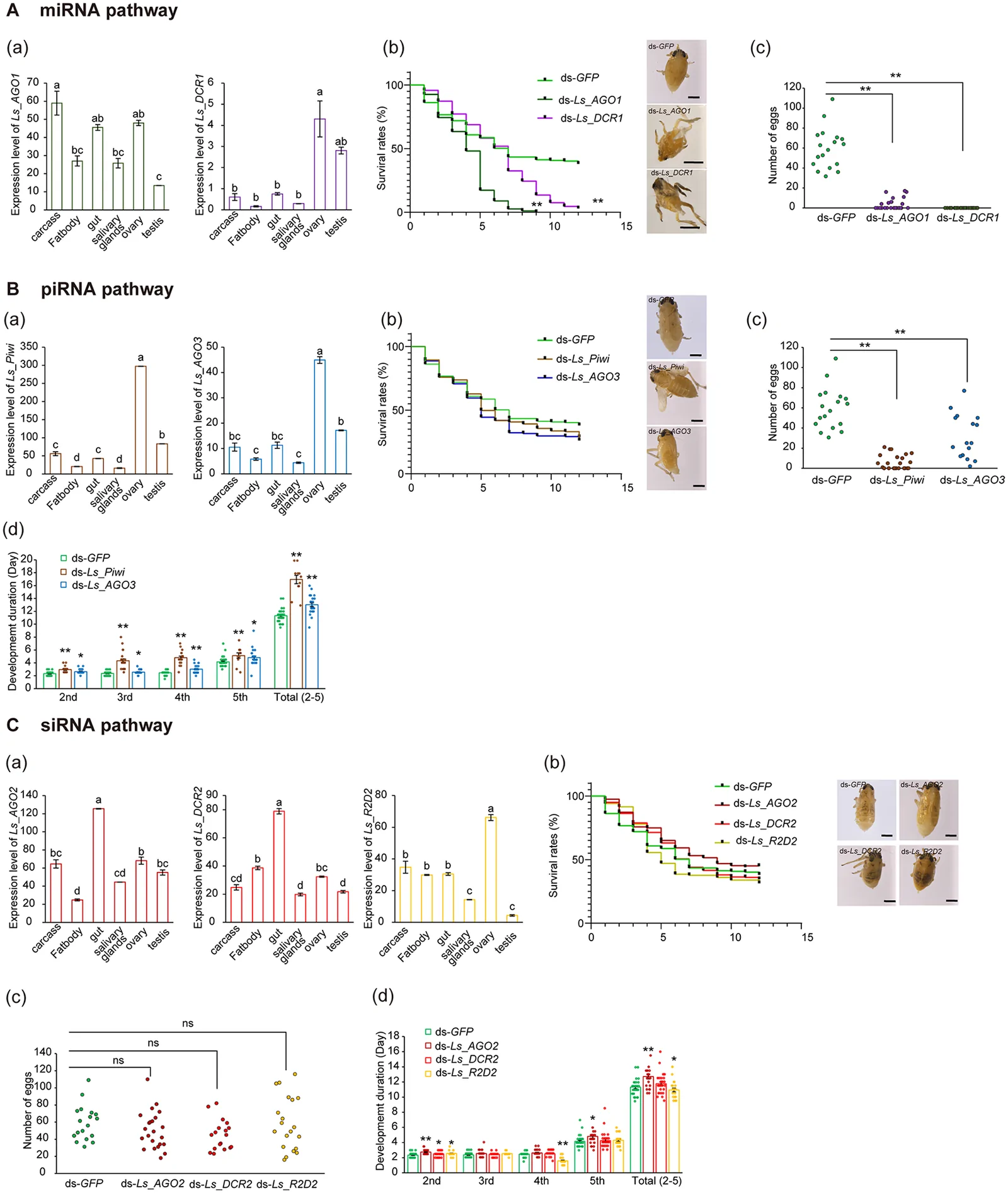
Tissue expression patterns and knockdown effects on biological properties of the three RNAi pathways key genes in *Laodelphax striatellus.* (**A**) miRNA pathway: **(a)** Relative transcript levels of *Ls_AGO1* and *Ls_DCR1* in different tissues of *L. striatellus*. Experiments were conducted with three independent biological replicates, each comprising at least 15 insects. **(b)** Survival rates of ds-*Ls_AGO1* or ds-*Ls_DCR1* treated insects compared to ds-*GFP* control. n indicates the number of insects per group. ds-*GFP*, n = 189. ds-*Ls_AGO1*, n = 189. ds-*Ls_DCR1*, n = 237. **(c)** Fecundity of ds-*Ls_AGO1* or ds-*Ls_DCR1* treated insects compared to ds-*GFP* control. Each dot represents a single female insect. n ≥ 19. (**B**) piRNA pathway: **(a)** Relative transcript levels of *Ls_Piwi* and *Ls_AGO3* in different tissues of *L. striatellus*. Experiments were conducted with three independent biological replicates, each comprising at least 15 insects. **(b)** Survival rates of ds-*Ls_Piwi* or ds-*Ls_AGO3* treated insects compared to ds-*GFP* control. ds-*GFP*, n = 189. ds-*Ls_Piwi*, n = 199. ds-*Ls_AGO3*, n = 205. **(c)** Fecundity of ds-*Ls_Piwi* or ds-*Ls_AGO3* treated insects compared to ds-*GFP* control. n ≥ 19. **(d)** Development duration of ds-*Ls_Piwi* or ds-*Ls_AGO3* treated insects compared to ds-*GFP* control. 2^nd^: second instar nymph; 3^rd^: third instar nymph; 4^th^: fourth instar nymph; 5^th^: fifth instar nymph; Total (2–5): Total development duration from 2^nd^ to 5^th^ instar nymphs. Each dot represents a single insect. n ≥ 21. (**C**) siRNA pathway: **(a)** Relative transcript levels of *Ls_AGO2*, *Ls_DCR2* and *Ls_R2D2* in different tissues of *L. striatellus*. Experiments were conducted with three independent biological replicates, each comprising at least 15 insects. **(b)** Survival rates of ds-*Ls_AGO2*, ds-*Ls_DCR2* or ds-*Ls_R2D2* treated insects compared to ds-*GFP* control. ds-*GFP*, n = 189. ds-*Ls_AGO2*, n = 243. ds-*Ls_DCR2*, n = 255. ds-*Ls_R2D2*, n = 220. **(c)** Fecundity of ds-*Ls_AGO2*, ds-*Ls_DCR2* or ds-*Ls_R2D2* treated insects compared to ds-*GFP* control. n ≥ 19. **(d)** Development duration of ds-*Ls_AGO2*, ds-*Ls_DCR2* or ds-*Ls_R2D2* treated insects compared to ds-*GFP* control. 2^nd^: second instar nymph; 3^rd^: third instar nymph; 4^th^: fourth instar nymph; 5^th^: fifth instar nymph; Total (2–5): Total development duration from 2nd to 5th instar nymphs. Each dot represents a single insect. n ≥ 21. ANOVA test was used to analyze tissues expression. Two-tailed student’s *t*-test was used to analyze the number of eggs and development duration. The Log-rank (Mantel-Cox) test was used to analyze survival curves. Error bars represent the standard error of the mean (SEM) (*, *p* < 0.05 and **, *p* < 0.01).

For the piRNA pathway, both *Ls_Piwi* and *Ls_AGO3* of *L. striatellus* were highly expressed in the ovary, followed by the testis (Fig 2B-a). High expression of *Ls_Piwi* and *Ls_AGO3* were observed in early stages of egg development (S1C-D Fig). The survival rates of the ds-*Ls_Piwi* and ds-*Ls_AGO3* treated insect groups (silencing efficiency shown in S2B Fig) were not significantly different from those of the control group at 12 days post injection. Most of the insect nymphs developed into adults, while two-thirds of the adults in the ds-*Ls_Piwi* treated group displayed abnormal wing curls (Fig 2B-b). Additionally, ds-*Ls_Piwi* injection severely reduced the production of eggs in adult *L. striatellus* (Fig 2B-c) and all of the eggs failed to hatch (S3 Fig and S1 Table). Similarly, decreased production of eggs was also observed for ds-*Ls_AGO3* treated insects compared to control (Fig 2B-c) and only approximately 20% of the eggs hatched into nymph stage (S3 Fig and S1 Table). Additionally, developmental duration of the 2^nd^-, 3^rd^-, 4^th^-, 5^th^-instar and the total development days (2^nd^- to 5^th^-instar nymphs) were significantly extended after knockdown of ds-*Ls_Piwi* and ds-*Ls_AGO3* in *L. striatellus* (Fig 2B-d).

For the siRNA pathway, *Ls_AGO2* and *Ls_DCR2* of *L. striatellus* were highly expressed in the gut compared to other tissues, while *Ls_R2D2* was most abundant in the ovary (Fig 2C-a). Relatively low expression of *Ls_R2D2* was observed in male adults of *L. striatellus* compared to other stages (S1G Fig), whereas no specific developmental stage expression patterns were noticed for *Ls_AGO2* and *Ls_DCR2* (S1E-S1F Fig). When knocking down these three key genes of siRNA pathway (silencing efficiency shown in S2C Fig), no significant effect on survival rates was observed in the ds-*Ls_AGO2*, ds-*Ls_DCR2* and ds-*Ls_R2D2* injection groups compared with the control group at 12 days post-injection (Fig 2C-b). No differences of the adult fecundity (Fig 2C-c) and egg hatchability (S3 Fig and S1 Table) were observed for insects injected with ds-*Ls_AGO2*, ds-*Ls_DCR2* and ds-*Ls_R2D2* compared to the control (ds-*GFP*) (Fig 2C-c). For the effects on the developmental stages, the duration of each 2^nd^- 5^th^-instar and total development days (2^nd^- to 5^th^-instar) were significantly extended in ds-*Ls_AGO2* treated insect group, while the duration of the 4^th^-instar and total development days were significantly shortened in ds-*Ls_R2D2* treated insect group (Fig 2C-d). Furthermore, prolonged development duration was only observed in the 2^nd^-instar for the insect injected with ds-*Ls_DCR2* (Fig 2C-d).

### miRNA and piRNA produced by corresponding pathways in *L. striatellus*

In the miRNA pathway, *DCR1* and *AGO1* play essential roles in producing functional miRNAs (~22 nt long) and executing gene silencing [52]. To explore the functions of miRNA pathway in *L. striatellus*, knockdown of *Ls_AGO1* or *Ls_DCR1* in third-instar nymphs was performed by injecting their corresponding dsRNAs. Compared to the control group (ds-*GFP*), the transcriptional expression of *Ls_AGO1* and *Ls_DCR1* were successfully inhibited with decreased level of 82% and 70%, respectively (Fig 3A-a). Our results showed that miRNA counts of the length 22 nt in the ds-*GFP* group were approximately 2 times and 1.5 times higher than those in the ds-*Ls_AGO1* and ds-*Ls_DCR1* treated groups, respectively (Fig 3A-b), suggesting that both *Ls_AGO1* and *Ls_DCR1* play a crucial role in the production of miRNAs similar to previous report in *D. melanogaster* [52].

**Fig 3:**
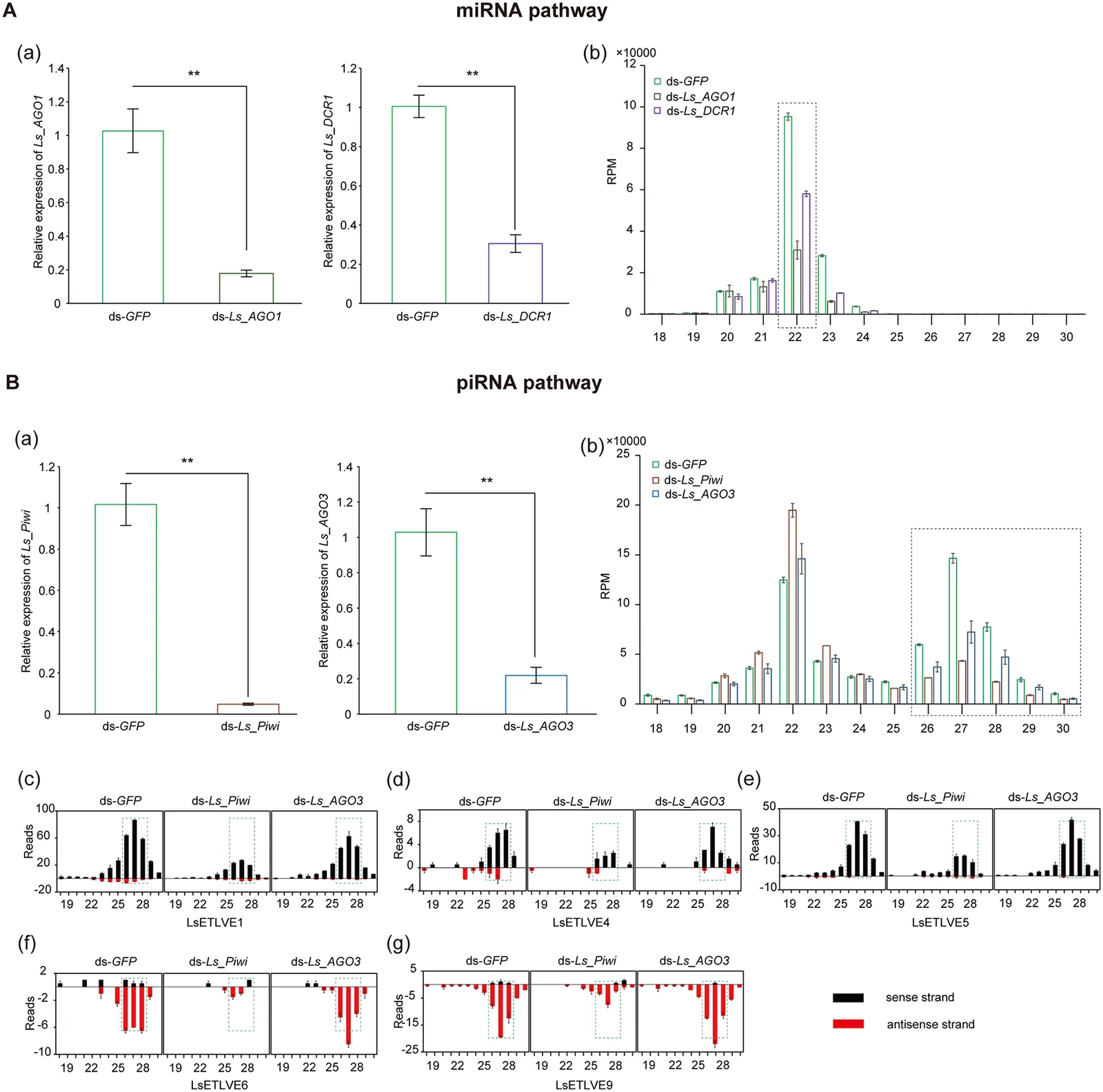
Effects of key gene knockdown on small RNA (sRNA) accumulation of miRNA and piRNA pathways in *Laodelphax striatellus.* **(A) (a)** Knockdown efficiency of *Ls_AGO1* and *Ls_DCR1*. **(b)** Accumulation of miRNA in ds-*Ls_AGO1* or ds-*Ls_DCR1* treated insects compared to ds-*GFP* control. Only annotated and predicted miRNAs are shown. **(B) (a)** Knockdown efficiency of *Ls_Piwi* and *Ls_AGO3*. **(b)** Accumulation of genomic sRNA in ds-*Ls_Piwi* or ds-*Ls_AGO3* treated insects compared to ds-*GFP* control. Accumulation of endogenous toti-like viral elements (LsETLVEs) (c-g) derived sRNA in ds-*Ls_Piwi* or ds-*Ls_AGO3* treated insects compared to ds-*GFP* control. The sense strand is shown in black, and the antisense strand is shown in red. RT-qPCR analyses in panels A(a) and B(a) were conducted with three independent biological replicates, each comprising at least 15 insects. Small-RNA sequencing analyses in panels A(b) and B(b-g) were performed with two independent biological replicates per treatment. Two-tailed student’s *t*-test was used to determine statistical significance for the RT-qPCR analyses. Error bars represent the SEM (**, *p* < 0.01).

The piRNA pathway is essential for the development and differentiation of animal germ cells [7,15,53], and has also been recently implicated in antiviral defense, particularly in mosquito cells [16]. To determine the functional role of piRNA pathway in *L. striatellus*, the core genes of this pathway, *Ls_Piwi* and *Ls_AGO3*, were efficiently inhibited using dsRNA injection approach in third-instar nymphs (Fig 3B-a). sRNA sequencing analysis revealed that the abundance of piRNA-sized reads (26–30 nt) was significantly reduced in insects treated with ds-*Ls_Piwi* or ds-*Ls_AGO3* compared to the ds-*GFP* control group (Fig 3B-b). Since piRNAs of *L. striatellus* are also produced from LsETLVEs as described previously [23], this type of piRNAs were subsequently analyzed. We found that piRNA reads derived from LsETLVEs (LsETLVE1, LsETLVE4, LsETLVE5, LsETLVE6, and LsETLVE9; the names and the sequences are derived from reference [23]) were also dramatically reduced in ds-*Ls_Piwi* treated insect group compared to the control (Fig 3B_c-g). These results demonstrate that *Ls_Piwi* is actively involved in production of piRNA-sized reads in *L. striatellus*. Although knockdown of *Ls_AGO3* did not significantly reduce abundance of EVE-derived piRNA-sized reads under our experimental conditions, we cannot rule out a role for *Ls_AGO3* in the piRNA pathway, as residual protein activity or limited ping-pong amplification in this species may have masked a detectable piRNA phenotype.

### AGO2 depletion, but not DCR2 or R2D2 depletion, enhances viral accumulation in *L. striatellus*

In insects, the siRNA pathway is widely recognized as a primary antiviral defense mechanism against RNA virus infection, and has been comprehensively characterized in model dipteran insects (e.g., *Drosophila* and mosquitoes) [8,33]. To evaluate the antiviral role of siRNA pathway in hemipteran insect, we efficiently knocked down three key genes in third-instar nymphs of *L. striatellus* (*Ls_AGO2*, *Ls_DCR2* and *Ls_R2D2*) (Fig 4A_a-c) and investigated their effects on the viral accumulation, as well as vsiRNA production derived from the four insect-specific RNA viruses (ISVs). Interestingly, our results indicated that significant increase of viral abundance and corresponding vsiRNA reads for the four ISVs (LSSolV2, LSSolV3, LSIfV2, and LSHV1) were observed only in the insect group treated with ds-*Ls_AGO2*, but not in those treated with ds-*Ls_DCR2* or ds-*Ls_R2D2*, compared to the control (ds-*GFP*) (Fig 4B_a-d). Notably, the distribution patterns of vsiRNAs - such as length profiles and strand polarity bias - remained unchanged between the ds-*Ls_AGO2* group and the other groups. Because vsiRNA abundance is influenced by viral RNA levels, we further normalized the viral small RNA read counts to the corresponding viral copy numbers determined by absolute qPCR quantification for each treatment group. Following normalization, the reads/copies ratio was significantly reduced in the *AGO2* knockdown group compared with the control, whereas no significant differences were observed in the *DCR2* or *R2D2* knockdown groups (S4 Fig). Additionally, similar to ISV (LSIfV2), increased viral accumulations of RSV (a plant virus transmitted by *L. striatellus*) were also confirmed when statistical analysis was conducted on individual insect treated with ds-*Ls_AGO2*, whereas no significant difference was observed in ds-*Ls_DCR2*-treated insects compared to GFP control (S5 Fig). Our results demonstrated that the three canonical siRNA-pathway components showed clearly different knockdown phenotypes in *L. striatellus*.

**Fig 4:**
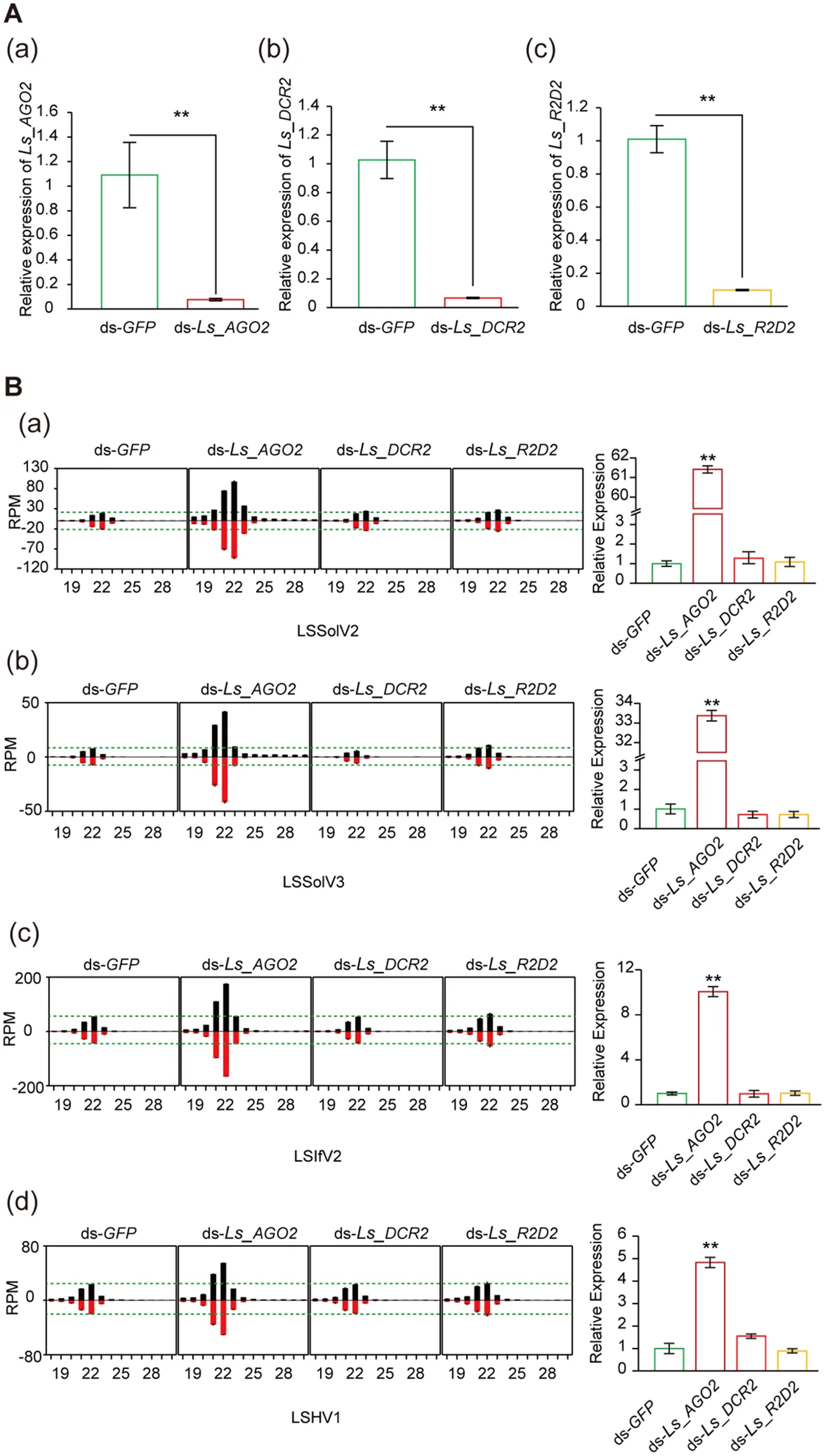
Knockdown of key siRNA pathway genes on viral mRNA and virus-derived small interfering RNAs (vsiRNAs) accumulation in *Laodelphax striatellus.* **(A)** Knockdown efficiency of *Ls_AGO2* **(a)**, *Ls_DCR2* **(b)**, and *Ls_R2D2* **(c)**. **(B)** Accumulation of vsiRNAs and viral abundance (LSSolV2 **(a)**, LSSolV3 **(b)**, *LS*IfV2 **(c)**, and *LS*HV1 **(d)**) in ds-*Ls_AGO2*, ds-*Ls_DCR2*, and ds-*Ls_R2D2* treated insects compared to ds-*GFP* control. The green dashed lines indicate the position of the 22-nt vsiRNA peak, facilitating visual comparison across treatment groups. RT-qPCR analyses of knockdown efficiency and viral abundance were conducted with three independent biological replicates, each comprising at least 15 insects. Small-RNA sequencing was performed with two independent biological replicates per treatment. Two-tailed student’s *t*-test was used to determine statistical significance, error bars represent the SEM (**, *p* < 0.01). Virus abbreviation: LSSolV2, Laodelphax striatellus sobeli-like virus 2; LSSolV3, Laodelphax striatellus sobeli-like virus 3; LSIfV2, Laodelphax striatellus iflavirus 2; LSHV1, Laodelphax striatellus honeydew virus 1.

Furthermore, potential antiviral roles for the conserved key genes of miRNA and piRNA pathway in *L. striatellus* were also investigated. Distribution patterns of four ISVs derived vsiRNA suggested that both the core genes of miRNA pathway (*Ls_AGO1* and *Ls_DCR1*) (S6A Fig) and piRNA pathway (*Ls_Piwi* and *Ls_AGO3*) (S6B Fig) are not involved in siRNA-based antiviral pathway. Additionally, although different levels of viral accumulation were observed for LSIfV2 (S6A Fig -c) and LSHV1 (S6A Fig -d) following knockdown of *Ls_AGO1* and *Ls_DCR1*, respectively, most dsRNA-treated samples showed no significant difference compared to the corresponding GFP controls (S6 Fig).

To test potential functional redundancy, we performed double and triple knockdown experiments by simultaneously injecting dsRNAs targeting *Ls_DCR1*/*Ls_DCR2* (double knockdown) and *Ls_DCR2*/*Ls_AGO2*/*Ls_R2D2* (triple knockdown) in third-instar insects (S7 Fig). In the double knockdown group (ds-*Ls_DCR1*/*DCR2*), no significant increase in viral abundance or vsiRNA reads was observed for any of the three ISVs (LSSolV2, LSIfV2, and LSHV1) compared to the ds-*GFP* control (S7C Fig), providing no detectable evidence for functional compensation between DCR1 and DCR2 under the tested knockdown conditions. In the triple knockdown group (ds-*Ls_DCR2*/*AGO2*/*R2D2*), a significant increase of viral abundance and corresponding vsiRNA reads was observed for the three ISVs (S7 Fig), driven by the inclusion of AGO2. Taken together, these knockdown experiments identified AGO2 as the only tested component whose depletion consistently increased viral accumulation, whereas depletion of DCR1, DCR2, or R2D2 did not produce comparable antiviral phenotypes. In parallel, we examined whether *DCR2* single knockdown or combined *DCR2*/*AGO2*/*R2D2* knockdown broadly altered small-RNA profiles. The size distributions of identified miRNAs were similar to those of the corresponding controls under both knockdown conditions. We further analyzed total clean small RNA reads (18–30 nt), and the overall size-distribution patterns were broadly maintained under both knockdown conditions, with a prominent 22-nt peak and an additional population of reads within the 25–30 nt range (S8 Fig).

To further examine RNAi pathway activity following knockdown of DCR1, DCR2, and AGO2, we performed an RNAi-of-RNAi assay in third-instar *L. striatellus*. In this assay, nymphs were first injected with dsRNA targeting *Ls_DCR1*, *Ls_DCR2*, or *Ls_AGO2*, and then re-injected 48 hours later with ds-*Ls_Tubulin* as a reporter of RNAi pathway activity. Knockdown efficiencies of *Ls_DCR1*, *Ls_DCR2*, and *Ls_AGO2* were confirmed by RT-qPCR (S9A Fig). Pre-knockdown of *Ls_DCR1*, *Ls_DCR2*, and *Ls_AGO2* reduced the cleavage efficiency of ds-*Ls_Tubulin* by 6.2%, 8.8%, and 76.5%, respectively (S9B Fig). Notably, only pre-knockdown of *Ls_AGO2* led to a statistically significant decrease in RNAi efficiency; pre-knockdown of *Ls_DCR1* or *Ls_DCR2* had no significant effect. Pre-knockdown of *Ls_DCR1* or *Ls_DCR2* did not detectably reduce silencing of the subsequently introduced *Ls_Tubulin* reporter, whereas *Ls_AGO2* pre-knockdown markedly impaired reporter silencing. Because residual DCR proteins may have remained after knockdown, this assay does not establish that *Ls_DCR2* is unnecessary for dsRNA processing.

### *DCR2* knockout using CRISPR/Cas9 results in embryonic lethality in *L. striatellus*

Although the dsRNA injection approach can efficiently reduce the expression of siRNA pathway genes by over 90% in *L. striatellus*, a major concern is that RNAi-mediated knockdown relies on these key genes to process the injected long dsRNA into siRNAs that mediate gene silencing. This dependency may result in incomplete silencing or self-limiting knockdown effects [34]. To address these limitations and rigorously validate the function of *Ls_DCR2*, we generated two independent *Ls_DCR2* mutant alleles using CRISPR/Cas9-mediated gene editing: mutation type 1 (a 5 bp deletion and 9 bp insert, *MT1*) (Fig 5A) and mutation type 2 (an 8 bp deletion, *MT2*) (Fig 5B). Since only heterozygous female mutants of *L. striatellus* were obtained in G_0_ generation, they were crossed with wild type (WT) male insects. Our results showed that heterozygous female and male mutants were present in both G_1_ and G_2_ generations; however, we failed to obtain homozygous mutant insects in the nymph and adult insects of the G_2_ generations (All hatched insects were either WT or heterozygous mutants. In *MT1*, the G2 generation consisted of 31 WT and 67 heterozygous mutant nymphs. In *MT2*, the G2 generation consisted of 45 WT and 74 heterozygous mutant nymphs), although they were expected (Fig 5C). Subsequently, we dissected and examined the unhatched eggs on rice seedlings from the G_2_ insects. Our results indicated that 29.41% (5/17) and 17.86% (5/28) of these eggs were homozygous mutants in *MT1* and *MT2*, respectively (Fig 5D, S2 Table), implying embryonic lethality in homozygous mutant insects.

**Fig 5:**
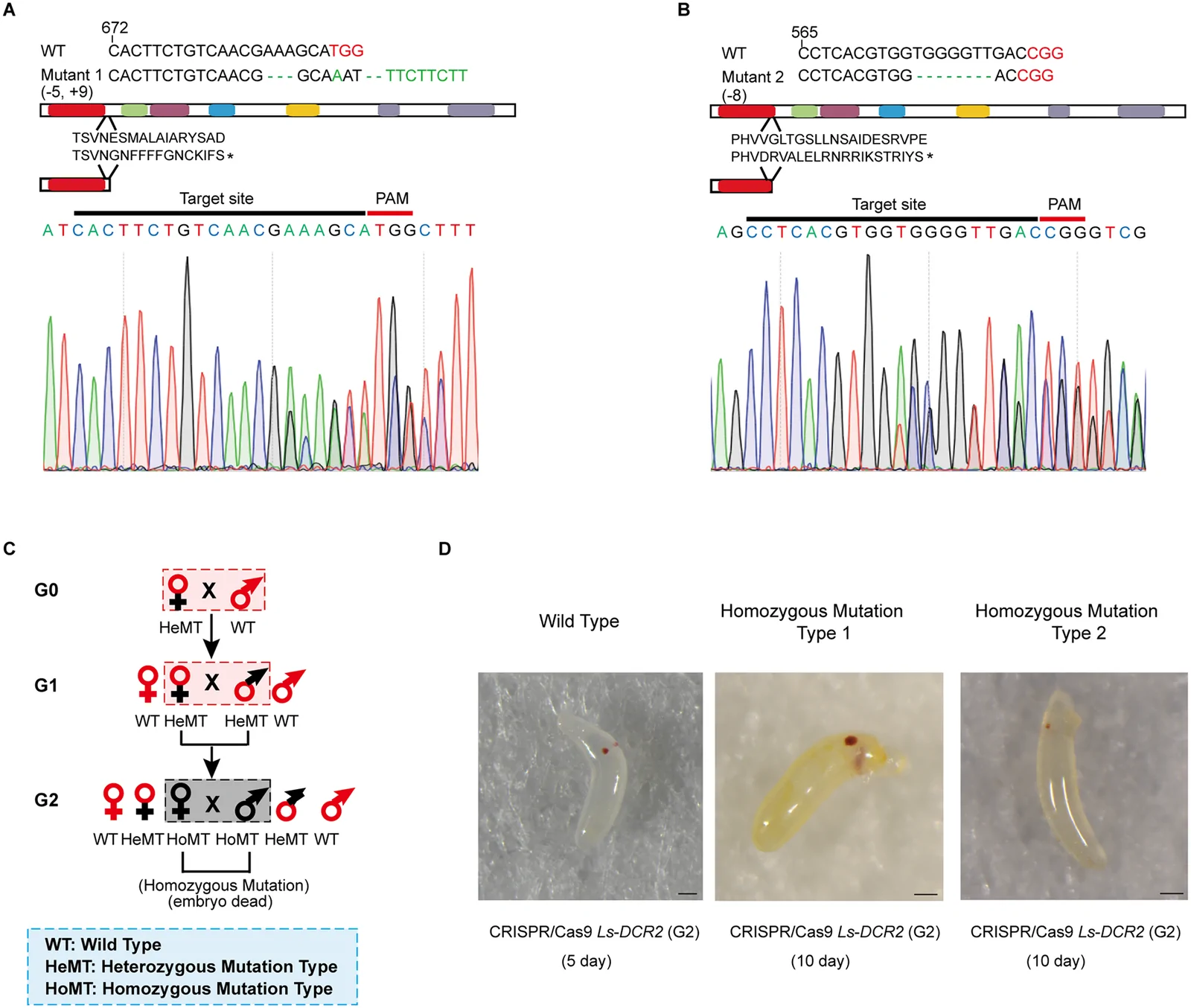
Effects of DCR2 knockout in *Laodelphax striatellus.* Schematic diagram of the single guide RNA (sgRNA)-targeted sites for mutation type 1 (*MT1*) (**A**) and mutation type 2 (*MT2*) (**B**) in G0 mutants. **(C)** Absence of homozygous *DCR2* mutant in *L. striatellus* G2 population. **(D)** Morphology of *MT1* and *MT2* eggs in G2 mutants of *L. striatellus* (Wild type used as control). Heterozygous female and male mutants were crossed and allowed to oviposit on fresh rice seedlings for one day. After removing the adults, unhatched eggs remaining on seedlings after 10 days were dissected and genotyped by Sanger sequencing. As a control, 5-day-old wild-type eggs were included for comparison. The scale bar represents 100 μm.

We further examined DCR2 accumulation in heterozygous mutants. The results showed that the expression levels of DCR2 were significantly reduced in both *MT1* and *MT2* (S10A Fig), compared with wild-type insects, whereas no significant difference in viral accumulation was observed (S10B-S10E Fig). Additionally, the population of heterozygous mutants exhibited a significant extension in the duration of the 1^st^ (*MT1*) and 5^th^ (*MT1* and *MT2*) nymph development stages compared to WT insects (S10F Fig). These results suggest that *Ls_DCR2* may play an essential role in the development, particularly during the early embryonic stage of *L. striatellus*.

### AGO2, but not DCR2, depletion enhances viral accumulation in three additional hemipteran species

To determine whether the contrasting *AGO2*- and *DCR2*-knockdown phenotypes observed in *L. striatellus* were also present in other hemipteran insects, we knocked down *AGO2* and *DCR2* in two other rice planthoppers (*S. furcifera* and *N. lugens*) of the family Delphacidae and in the whitefly *Bemisia tabaci* of another family Aleyrodidae. Firstly, we identified two ISVs of *S. furcifera* (identified by screening for previously reported ISVs and confirming by RT-PCR) and four ISVs of *N. lugens* in our laboratory population: Hangzhou Sogatella furcifera iflavirus 1 (SFIV1, accession no. MZ210055.1), Sogatella furcifera honeydew virus (SFHV, accession no. MG818986.1); Nilaparvata lugens bunya-like virus 1 (NLBV1, L: accession no. PQ433852; M: accession no. PQ433853; S: accession no. PQ433854), Nilaparvata lugens bunya-like virus 2 (NLBV2, L: accession no. PQ433855; M: accession no. PQ433856; S: accession no. PQ433857), Nilaparvata lugens bunya-like virus 3 (NLBV3, L: accession no. PQ433858; M: accession no. PQ433859; S: accession no. PQ433860), Nilaparvata lugens bunya-like virus 4 (NLBV4, L: accession no. OR789635; M: accession no. OR789636; S: accession no. OR789637). After dsRNA injection (third-instar insects), the relative transcript levels of *AGO2* and *DCR2* were significantly decreased in both *S. furcifera* (Fig 6A-a, b) and *N. lugens* (Fig 6B-a, b). As a result, both viral accumulation and the corresponding vsiRNA production were significantly increased only in the *AGO2*-silenced insect groups, but not in the *DCR2*-silenced ones, for *S. furcifera* (Fig 6A-c, d) and *N. lugens* (Fig 6B-c, d, e, f), which is consistent with the results observed in *L. striatellus* (Fig 4).

**Fig 6:**
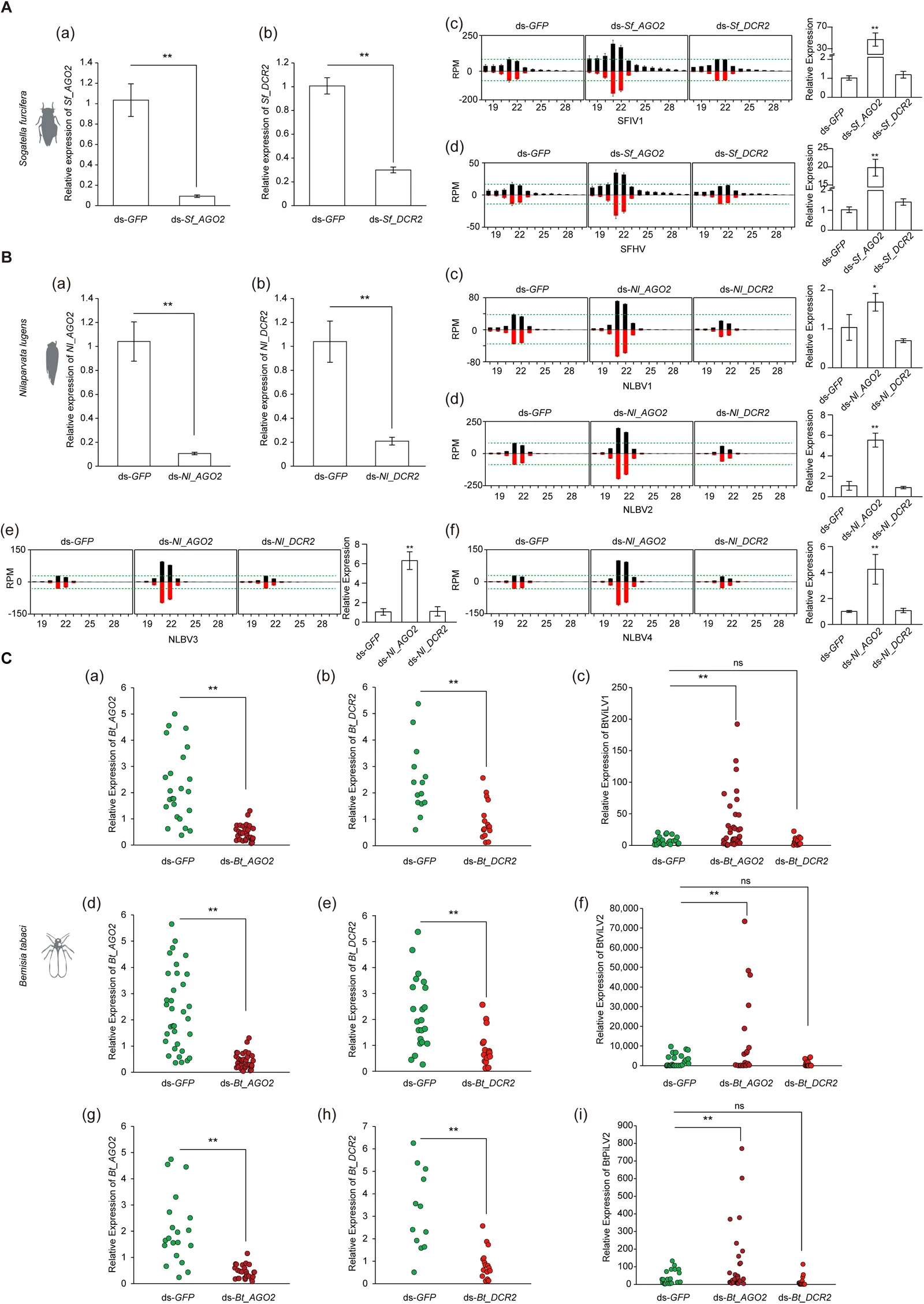
Knockdown of key siRNA pathway genes on viral mRNA and virus-derived small interfering RNAs (vsiRNAs) accumulation in other hemipteran insects. **(A)** Effects in *Sogatella furcifera*. Knockdown efficiency of *Sf*_*AGO2* (a) and *Sf*_*DCR2* **(b)**. Accumulation of vsiRNAs and viral abundance of SFIV1 (c) and SFHV (d) in ds-*Sf*_*AGO2* and ds-*Sf*_*DCR2* treated insects compared to ds-*GFP* control. RT-qPCR analyses were conducted with three independent biological replicates, each comprising at least 15 insects, whereas small-RNA sequencing was performed with two independent biological replicates per treatment. **(B)** Effects in *Nilaparvata lugens*. Knockdown efficiency of *Nl*_*AGO2* (a) and *Nl*_*DCR2* **(b)**. Accumulation of vsiRNAs and viral abundance (NLBV1 **(c)**, NLBV2 **(d)**, NLBV3 **(e)**, and NLBV4 **(f)**) in ds-*Nl*_*AGO2* and ds-*Nl*_*DCR2* treated insects compared to ds-*GFP* control. RT-qPCR analyses were conducted with three independent biological replicates, each comprising at least 15 insects, whereas small-RNA sequencing was performed with two independent biological replicates per treatment. **(C)** Effects in *Bemisia tabaci*. Knockdown efficiency of ds-*Bt*_*AGO2* (a, d, g) and ds-*Bt*_*DCR2* (b, e, h) of individual *B. tabaci*. Accumulation of viral abundance of BtViLV1 **(c)**, BtViLV2 **(f)**, and BtPiLV2 (i) in ds-*Bt*_*AGO2* and ds-*Bt*_*DCR2* treated insects compared to ds-*GFP* control. Each dot represents a single insect. n ≥ 12. Two-tailed student’s *t*-test was used to determine statistical significance, error bars represent the SEM (**, *p* < 0.01). Virus abbreviation: SFIV1, Hangzhou Sogatella furcifera iflavirus 1; SFHV, Sogatella furcifera honeydew virus; NLBV1, Nilaparvata lugens bunya-like virus 1; NLBV2, Nilaparvata lugens bunya-like virus 2; NLBV3, Nilaparvata lugens bunya-like virus 3; NLBV4, Nilaparvata lugens bunya-like virus 4; BtViLV1, Bemisia tabaci virga-like virus 1; BtViLV2, Bemisia tabaci virga-like virus 2; BtPiLV2, Bemisia tabaci picorna-like virus 2.

The whitefly *B. tabaci*, a globally devastating pest belonging to a different family than rice planthoppers [54,55], was subsequently used to investigate the siRNA-mediated antiviral pathway. Based on a previous report of the *B. tabaci* virome [56], we identified three ISVs in our laboratory population using RT-PCR: Bemisia tabaci virga-like virus 1 (BtViLV1, accession no. MW256664.1), Bemisia tabaci virga-like virus 2 (BtViLV2, accession no. MW256665.1), and Bemisia tabaci picorna-like virus 2 (BtPiLV2, accession no. MW256669.1). The effects on the viral accumulation of these three whitefly ISVs were also investigated following knockdown of *Bt-AGO2* (accession no. KF192313.1) and *Bt-DCR2* (accession no. KF740508.1) in one-week-old adults. Similar to the results observed in rice planthoppers, silencing *Bt-AGO2* significantly increased the accumulation of the three RNA viruses in individual insects, whereas no such effect was observed in *Bt-DCR2*-silenced ones (Fig 6C). These findings suggest that the contrasting knockdown phenotypes of AGO2 and DCR2 are shared among the four hemipteran species examined. Whether this reflects incomplete DCR2 depletion, compensatory processing activity, or lineage-specific organization of the antiviral siRNA pathway remains unresolved.

## Discussion

The three conserved small silencing RNA pathways - miRNA, piRNA, and siRNA - are essential for host biology and antiviral defense, as has been comprehensively demonstrated in dipteran insects, particularly in mosquitoes and fruit flies [3]. However, their functions remain largely unexplored in non-model insects. This study functionally compared core components of the three major small-RNA pathways in *L. striatellus*. We found that AGO2 depletion consistently enhanced the accumulation of multiple RNA viruses, whereas comparable antiviral phenotypes were not detected after depletion of DCR2 or R2D2. Similar contrasting knockdown responses were observed in three additional hemipteran species. These findings reveal unexpected functional divergence among conserved siRNA-pathway components in hemipteran insects and highlight the need to determine how antiviral vsiRNAs are generated in this lineage.

In insects, the miRNA pathway plays a crucial role in maintaining physiological homeostasis by regulating processes such as development, metamorphosis, immune responses, and reproduction [11,12]. In this study, knockdown of either *Ls_AGO1* or *Ls_DCR1* of *L. striatellus* resulted in a noticeable reduction of 22-nt miRNAs compared to *GFP* control (Fig 3A). Additionally, suppression of *Ls_AGO1* or *Ls_DCR1* significantly affected the survival rate, ecdysis, and reproduction of *L. striatellus* (Fig 2A). Similarly, recent studies demonstrated that depletion of *AGO1* or *DCR1* disrupted survival, molting, and reproduction in three other hemipteran insects, *N. lugens*, *S. furcifera*, and *B. tabaci* [13,20,21,57], suggesting that the miRNA pathway is conserved in hemipterans during key physiological process.

The piRNA pathway mainly plays a role in germ cells, and Piwi mutations in female and male *Drosophila* cannot renew germline stem cells, leading to germline degeneration and sterility [15,58]. AGO3 mutant female flies have been reported to be sterile, with homozygous AGO3 mutant females laying fewer eggs and failing to hatch compared to wild-type or heterozygous females [14]. In *L. striatellus*, our results showed that adult fecundity and the egg hatchability were severely affected compared to the control when *Ls_Piwi* and *Ls_AGO3* were silenced (Fig 2B-c, S3 Fig, and S1 Table). A recent report also revealed that *Ago3* and *Piwi* of another rice planthopper (*N. lugens*) significantly disrupt female reproductive processes [59], indicating their essential roles in germ cell development of insects. Interestingly, we found that the abundance of EVE piRNA-sized reads (26–29 nt) was significantly reduced in *L. striatellus* only when *Ls_Piwi* was silenced, but not when *Ls_AGO3* was targeted (Fig 3B). In contrast, in *N. lugens*, silencing either of *Piwi* or *Ago3* completely eliminated the piRNA peak [59], highlighting the divergent roles of these proteins across hemipteran insects. The lack of a detectable piRNA-sized reads reduction upon *Ls_AGO3* knockdown should be interpreted cautiously because it may reflect incomplete protein depletion or low baseline ping-pong amplification activity in *L. striatellus* rather than a genuine functional absence of AGO3 in the piRNA pathway.

Although all the three major small silencing RNA pathways (miRNA, piRNA, and siRNA) contribute to combating RNA virus infections, the siRNA pathway is generally considered as the primary one in insects, with three key genes - *AGO2*, *DCR2*, and *R2D2* - playing critical roles in this process [16,17,44,60]. Unexpectedly, knockdown experiments revealed that only repression of *Ls_AGO2*, but not *Ls_DCR2* or *Ls_R2D2*, resulted in a significant increase for the accumulation of both viral mRNA and vsiRNA compared to control in *L. striatellus* infected with four ISVs (LSSolV2, LSSolV3, LSIfV2, and LSHV1) (Fig 4B) and a plant virus (RSV) (S5C Fig). It should also be noted that the peak length of vsiRNAs (21- and 22-nt) was maintained in ds-*Ls_DCR2* and ds-*Ls_R2D2* treated insects compared to control. In dipteran insects, DCR2 is an RNase III enzyme that recognizes and cleaves dsRNA of viral origin into 21-nt siRNAs [29,34]. In *DCR2*-null sibling mosquitoes (*A. aegypti*), the abundance of 21-nt vsiRNA was significantly reduced and the 21-nt length peak also disappeared compared to the wild mosquitoes. Moreover, the insect was acutely susceptible to both arbovirus and the insect-specific alphavirus in *DCR2* mutant mosquitoes, suggesting the critical roles of DCR2 in siRNA-mediated antiviral defense [61–63]. To account for differences in viral RNA abundance among treatment groups, we further normalized the raw vsiRNA read counts to the corresponding viral RNA copy numbers determined by absolute qPCR quantification. Following normalization, the reads/copies ratio was significantly reduced in *Ls_AGO2*-knockdown insects, whereas no significant difference was observed following *Ls_DCR2* or *Ls_R2D2* knockdown compared with the control (S4 Fig). Together with the preservation of canonical vsiRNA size profiles and strand polarity following *Ls_DCR2* knockdown, these results indicate that *Ls_DCR2* depletion did not detectably alter vsiRNA accumulation relative to viral RNA abundance under the tested conditions. Moreover, the RNAi-of-RNAi assay using a non-replicating reporter substrate provided complementary evidence that *Ls_DCR2* knockdown did not detectably impair RNAi activity.

“RNAiof RNAi” assays indicated that pre-knockdown of *Ls_AGO2* significantly impaired cleavage of subsequently injected ds-*Ls_Tubulin*, whereas pre-knockdown of *Ls_DCR1* or *Ls_DCR2* had no significant effect, demonstrating that DCR2 pre-knockdown did not detectably alter reporter silencing, but residual DCR2 activity could not be excluded in *L. striatellus* (S9 Fig). On the contrary, in *Locusta migratoria* (Orthoptera), silencing of *Lm-DCR1*, *Lm-DCR2a* and *Lm-DCR2b* prior to injection of ds-*Lm-Tubulin* diminished RNAi efficiency for *Lm-Tubulin*, suggesting that all the three *Lm-Dicers* contribute to RNAi [64]. Similarly, RNAi-related factor in *Schistocerca gregaria* (Orthoptera), *Tribolium* (Coleoptera), *Leptinotarsa decemlineata* (Coleoptera), and *Chilo suppressalis* (Lepidoptera) can also affect RNAi efficiency [65–68]. These findings differ from our knockdown observations in *L. striatellus*, raising the possibility that antiviral siRNA pathway organization may vary among insect lineages. However, broader taxonomic sampling and direct genetic analyses will be required to determine whether the observed phenotype is widespread within Hemiptera. Additionally, we observed typical vsiRNA profiles in wild-type planthoppers that are consistent with processing by a Dicer-family enzyme, raising the possibility that another nuclease, residual DCR2 activity, or an alternative processing mechanism may contribute to vsiRNA production in *L. striatellus*. Identifying a putative alternative nuclease would represent an important direction for future research. To further confirm the function of *Ls_DCR2*, CRISPR/Cas9 was performed to knockout *DCR2* in *L. striatellus*. Unexpectedly, we failed to obtain homozygous mutant insects in the G2 generations because the loss of *DCR2* caused insect embryonic lethality (Fig 5). Furthermore, a significant extension in the duration of nymph development stages (1^st^ and 5^th^) was observed for the two heterozygous mutants compared to WT insects (S10 Fig), demonstrating an essential developmental role for DCR2, while its antiviral contribution could not be directly assessed in homozygous mutants of *L. striatellus*. Similarly, a previous report suggested that *AGO2* and *DCR2* of *L. striatellus* do not actively respond to RSV infection although *AGO2* knockdown resulted in increased viral RNA levels [69]. Additionally, interference of *DCR2* resulted in the disrupting of the molting and wing expansion in *S. furcifera* [50]. Furthermore, to elucidate the antiviral roles of *AGO2* and *DCR2* in hemipteran insects beyond *L. striatellus*, we investigated species from two different genera (*Nilaparvata* and *Sogatella*) of the family Delphacidae, as well as from another family, Aleyrodidae. Consistent with the results in *L. striatellus*, AGO2 depletion, but not DCR2 depletion, produced a detectable increase in viral accumulation in all three additional species examined (Fig 6). Given the high abundance of vsiRNAs generated in hemipteran insects, their biogenesis may involve residual DCR2 activity, another nuclease, or an alternative processing mechanism that remains to be identified. Collectively, our findings reveal a reproducible contrast between AGO2- and DCR2-knockdown phenotypes in the four hemipteran species examined. These observations suggest that the canonical DCR2-centered model derived from dipteran insects may not fully explain the measured antiviral RNAi responses in these species. Future studies aimed at identifying the nuclease responsible for antiviral vsiRNA biogenesis, or conditionally eliminating DCR2 activity, will be essential for resolving the organization of antiviral RNAi pathways in hemipteran insects.

## Materials and methods

### Insect cultures

The three rice planthopper species, *L. striatellus*, *N. lugens* and *S. furcifera*, were reared in the laboratory, separately on seedlings of rice (*Oryza sativa* Xiushui) in a growth chamber at 26 ± 1°C, 80% relative humidity, and a 14 h light/10 h dark photoperiod. The whitefly *B. tabaci* (MED) was originally collected from Ningbo, Zhejiang province and maintained on cotton plants in the laboratory at Ningbo University.

### RNA extraction and quantitative real-time PCR

Total RNA was isolated from insects (approximately 15 planthoppers or a single whitefly) using the RNAiso Plus (Takara, Japan) according to the manufacturer’s instructions. The quality of RNA was detected using a NanoDrop 2000 spectrophotometer (Thermo Fisher Scientific, USA). Total RNA was subjected to reverse transcription to complementary DNA (cDNA) based on HiScript II Q RT SuperMix for qPCR (+gDNA wiper) (Vazyme, China). Quantitative real-time PCR (RT-qPCR) was performed on a LightCycler Real-Time PCR System (Roche, Switzerland) with Hieff qPCR SYBR Green Master Mix (Low Rox Plus) (Yeasen, China). The expression level of each gene in *L. striatellus* was normalized to the housekeeping gene *ACTIN* and was calculated with the 2^-∆∆Ct^ method. Each experiment was performed with three biological replicates, and two technical replicates were set up for each biological replicate.

### Phylogenetic analysis

The predicted protein sequences of key small silencing RNA pathway genes of *L. striatellus* were aligned with those of other insect species (*D. melanogaster*, *Tribolium castaneum*, *Caenorhabditis elegans*, *Apis florea*, *N. lugens*, and *S. furcifera*) which were retrieved from NCBI protein database. The conserved domains of these genes were determined with NCBI CD-Search tools (https://www.ncbi.nlm.nih.gov/Structure/bwrpsb/bwrpsb.cgi) and their genome structures were then visualized by TBtools [70]. Phylogenetic analysis was constructed based on their full-length protein sequences using MEGA 6 software with maximum likelihood method.

### Double-stranded RNA synthesis and injection

dsRNAs of the key RNAi-related genes (*L. striatellus*: *AGO1*, *AGO2*, *DCR1*, *DCR2*, *R2D2*, *Piwi*, *AGO3*; *N. lugens*: *AGO2*, *DCR2*; *S. furcifera*: *AGO2*, *DCR2*; *B. tabaci*: *AGO2*, *DCR2*) and *GFP* were synthesized with T7 High Yield Transcription Kit (Vazyme, China) in line with the manufacturer’s protocol. Primers (with T7 promoter sequences) for these genes are shown in S3 Table. The quality of synthesized dsRNA was determined using agarose gel electrophoresis. The volumes of dsRNA (500 ng/μL) injected into 1^st^-, 3^rd^-instar nymphs and one-day-old adults were approximately 10 nL, 25 nL, 50 nL, respectively. dsRNA was injected into hemolymph through ventral thorax of the insect using a glass needle which was operated with a TransferMan 4r micromanipulator (Eppendorf, Germany). After injection, the nymphs were placed on fresh rice seedlings grown in glass tubes 5 cm in diameter.

### Injection of virus crude extracts

Approximately 10 RSV-infected adult insects were homogenized with toothpicks in 100 μL of 10 mM PBS (pH 7.4) in a 1.5 mL EP tube. After centrifugation at 8,000 rpm at 4°C for 5 min, the supernatant was retained. The process was repeated three times and the supernatant of the last centrifugation was used as the viral crude extracts for injection. A total of 25 nL of viral crude extracts was injected into the hemolymph of 3^rd^-instar insect nymphs and then transferred to fresh rice seedlings for rearing.

### Fecundity, survival and developmental duration

To investigate the functions of key small silencing RNA-related genes in planthopper, corresponding dsRNAs were injected into one-day-old (newly emerged) females and males of *L. striatellus*. Then, one female and two male insects were transferred into a glass tube with a rice seedling for three days. Subsequently, the males were removed and the female was maintained in the tube for another ten days before removal. To determine hatch ratio of the planthopper eggs, rice seedlings were dissected ten days after removal of the females, and the number of unhatched eggs was recorded. The number of effective groups for each gene is at least 19 for this experiment. Moreover, to investigate the effects of these genes on the insect survival, 3^rd^-instar nymphs of *L. striatellus* were used for dsRNA injection. Three groups were set up for each gene, with approximately 100 insects per group. Two days after injection, the surviving insects were recorded daily for next 12 days. Additionally, to evaluate the effects of the genes on insect developmental stages, the 1^st^-instar nymphs were injected with corresponding dsRNA. Each nymph was individually maintained in a glass tube and the developmental stage was recorded at 12-hour intervals until natural death. The number of effective groups for each gene is at least 20 for this experiment. Gene silencing efficiency was assessed in approximately 30 insects at two days post dsRNA injection.

### Tissue- and developmental stage-specific expression patterns of key RNAi genes in *L. striatellus*

To investigate tissue-specific expression patterns of small silencing RNA-related genes in *L. striatellus*, different tissues were dissected from 3-day-old females and males, including carcass, fat body, gut, salivary glands, ovary and testis. For the developmental expression patterns, egg (12h, 24h, 48h, 96h, 144h, 168h), 1^st^-instar nymphs (0h, 12h, 24h), 2^nd^-instar nymphs (0h, 12h, 24h, 36h), 3^rd^-instar nymphs (0h, 12h, 24h, 36h, 48h, 60h), 4^th^-instar nymphs (0h, 12h, 24h, 36h,48h, 60h), 5^th^-instar nymphs (0h, 12h, 24h, 36h, 48h, 60h, 72h), female and male adults (0h, 12h, 24h, 48h, 96h, 144h, 192h) were used. Then the RNA was extracted by RNAiso Plus and the expressions were determined by qRT-PCR as described above.

### Insect-specific RNA viruses identified in the lab population of *L. striatellus*

Besides rice viruses, *L. striatellus* also harbors a large number of different ISVs [49]. To further explore the antiviral roles of the three small silencing RNA pathways, viruses presented in the lab population of *L. striatellus* were investigated. Virus screening indicated that, in addition to RSV, seven ISVs were identified in the lab population of *L. striatellus* through RT-PCR detection and Sanger sequencing confirmation (S4 Table). Moreover, infection rates of these ISVs in individual insects were evaluated. Our results demonstrated that the infection rates of these ISVs varied from 28.9% to 97.8% in the insect population (S11 Fig). Considering that Himetobi P virus derived vsiRNAs exhibit non-canonical pattern [71], four ISVs with infection rate higher than 60% were selected for subsequent antiviral response analysis in *L. striatellus*, including Laodelphax striatellus sobeli-like virus 2 (LSSolV2, accession no. BK068174), Laodelphax striatellus sobeli-like virus 3 (LSSolV3, accession no. BK068175), Laodelphax striatellus iflavirus 2 (LSIfV2, accession no. PP781946), and Laodelphax striatellus honeydew virus 1 (LSHV1, accession no. NC_023627.1).

### RNA-seq analysis

Total RNA was extracted from *L. striatellus* for transcriptome sequencing and small RNA (sRNA) sequencing. Specifically, transcriptome sequencing was performed on the Illumina HiSeq 4000 platform and sRNA was sequenced by Illumina HiSeq 2500 platform (Novogene, Tianjin, China). For sRNA sequencing, two independent biological replicates were analyzed for each treatment. *De novo* assembly of transcriptome was accomplished based on clean reads from all libraries using Trinity with default parameters [72]. Adapter, low quality and junk sequences were removed from the raw reads of sequenced sRNAs, as described previously [48]. For the total small RNA size-distribution analysis shown in S8 Fig, all quality-controlled 18–30 nt reads were grouped according to read length and normalized as reads per million (RPM). Afterwards, sRNAs with the length of 18–30 nt were fetched and mapped into the genome sequences of the identified viruses using Bowtie software (allowing zero mismatch). Virus-derived small RNAs (vsiRNAs) were extracted for downstream analyses, including size distribution and genomic mapping profiles, using Linux bash scripts. The abundance of vsiRNAs was calculated as RPM, defined as the number of vsiRNA reads × 10⁶ divided by the total number of reads in each sample. For the analysis shown in S4 Fig, the raw vsiRNA read count for each virus was further normalized to the corresponding viral RNA abundance by dividing the vsiRNA read count by the viral RNA copy number determined by absolute qPCR quantification. The resulting reads/copies ratio was used to represent vsiRNA abundance relative to viral RNA abundance. To analyze piRNA-sized reads abundance, quality-controlled sRNA reads were mapped to the *L. striatellus* genome or to endogenous viral element (EVE) sequences. To analyze miRNA abundance, quality-controlled sRNAs mapped to the *L. striatellus* genome were sequentially aligned to the miRBase microRNA database (allowing zero mismatches), followed by genomic repeat sequences, mRNA sequences, intronic regions, and ncRNA databases (allowing up to two mismatches). Small RNAs were subsequently classified and annotated using custom Python scripts, which are publicly available in a GitHub repository (https://github.com/Zhuangxinye/vsirna-pipeline).

### RNAi of RNAi assay

An RNAi of RNAi assay was conducted to assess the effect of *Ls_DCR1*, *Ls_DCR2* and *Ls_AGO2* on RNAi efficiency in *L. striatellus*. Third instar nymphs were injected with dsRNA into the hemocoel. After 48 h, nymphs of the ds-*Ls_DCR1*, ds-*Ls_DCR2* and ds-*Ls_AGO2* or ds-*GFP*-injected groups were re-injected with equal doses of ds-*Ls_Tubulin*, whereas nymphs from an additional ds-*GFP*-injected group were reinjected with ds-*GFP* as a double-injection negative control. 24 hours after the second injection, total RNA was extracted from single *L. striatellus* individuals by using RNAiso Plus (Takara, Japan) as described above, and the expression level of the *Ls_Tubulin* gene was analyzed.

### CRISPR/Cas9-mediated knockout of *Ls*_*DCR2*

The potential target sites for synthesizing sgRNA for *Ls*_*DCR2* were predicted using the sgRNAcas9 algorithm 3.0.5 [73]. The search parameters were set as follows: sgRNA length of 20 nucleotides, GC content between 20–80%, and NGG as the PAM sequence. As a result, two sgRNAs with lowest off-target possibility were selected (5’-CCTCACGTGGTGGGGTTGAC-3’; 5’-CACTTCTGTCAACGAAAGCA-3’). PCR was performed using a forward primer containing the T7 sequence and a reverse primer containing the partial sgRNA sequence (S3 Table). The sgRNA was prepared using a T7 High Yield RNA Transcription Kit (Vazyme, China) according to the manufacturer’s instructions. The Cas9 protein (GenScript, China) and sgRNA were mixed and then microinjected using the TransferMan 4r micromanipulator (Eppendorf, Germany). In brief, a mixture of sgRNA (300 ng/μl) and Cas9 protein (8 μg) were injected into newly deposited eggs. The injected eggs were then transferred carefully to filter papers that were rinsed with sterilized water (containing tebuconazole (20 ng/ml) and kanamycin (50 ng/ml)) and placed in an incubator at 26 ± 0.5 °C with a humidity level of 50 ± 5%. At 48 hours after injection, genomic DNA was extracted from injected eggs, and the mutation of the sgRNA target sites was confirmed by PCR followed by Sanger sequencing. The wings of newly emerged adults (G0) were carefully detached and used for mutation detection. Then the mutated individuals were mated with wild-type insects to produce G1. In G1, the mutated females were mated with mutated males to produce G2 to obtain purified mutant insect strain.

### Statistical analysis

All the data were analyzed using the IBM SPSS Statistics (ver. 23.0) Software. Data were shown as means ± SEM. For pairwise comparisons, statistical significance was determined using Student’s *t* test. Significance was reported as *P < 0.05, **P < 0.01. For multiple comparisons, statistical significance was determined using one-way analysis of variance (ANOVA) with Tukey’s test. For survival curves, statistical significance was determined using the Log-rank (Mantel-Cox) test.

## Supporting information

S1 Fig Relative expression of *Ls*_*AGO1* (A), *Ls*_*DCR1* (B), *Ls*_*Piwi* (C), *Ls*_*AGO3* (D), *Ls*_*AGO2* (E), *Ls*_*DCR2* (F), and *Ls*_*R2D2* (G) genes at different developmental stages of *Laodelphax striatellus.* Error bars represent the SEM.
(TIF)

S2 Fig (A) Knockdown efficiency of *Ls*_*AGO1* and *Ls*_*DCR1.* (**B**) Knockdown efficiency of *Ls*_*Piwi* and *Ls*_*AGO3*. (**C**) Knockdown efficiency of *Ls*_*AGO2*, *Ls*_*DCR2* and *Ls*_*R2D2*. Experiments were conducted with three independent biological replicates, each comprising at least 15 insects. Two-tailed student’s *t*-test was used to determine statistical significance, error bars represent the SEM (**, *p* < 0.01).
(TIF)

S3 Fig Analysis of egg hatch rates after knockdown of *Ls*_*AGO1*, *Ls*_*Piwi*, *Ls*_*AGO3*, *Ls*_*AGO2*, *Ls*_*DCR2*, and *Ls*_*R2D2* compared to the *Ls* *GFP* control. Two-tailed student’s *t*-test was used to determine statistical significance (**, *p* < 0.01).
(TIF)

S4 Fig Effects of knockdown of key siRNA pathway genes on vsiRNAs abundance normalized to viral RNA copy number in *Laodelphax striatellus.* The raw vsiRNA read count for each virus was divided by the corresponding viral RNA copy number determined by absolute qPCR quantification, and the resulting reads/copies ratio was used to represent vsiRNA abundance relative to viral RNA abundance. (**A**) Normalized vsiRNA abundance of LSSolV2 in ds-*GFP* control (a), ds-*Ls_AGO2* (b), ds-*Ls_DCR2* (c), and ds-*Ls_R2D2* (d) treated insects. (**B**) Normalized vsiRNA abundance of LSSolV3 in ds-*GFP* control (a), ds-*Ls_AGO2* (b), ds-*Ls_DCR2* (c), and ds-*Ls_R2D2* (d) treated insects. (**C**) Normalized vsiRNA abundance of LSIfV2 in ds-*GFP* control (a), ds-*Ls_AGO2* (b), ds-*Ls_DCR2* (c), and ds-*Ls_R2D2* (d) treated insects. (**D**) Normalized vsiRNA abundance of LSHV1 in ds-*GFP* control (a), ds-*Ls_AGO2* (b), ds-*Ls_DCR2* (c), and ds-*Ls_R2D2* (d) treated insects.
(TIF)

S5 Fig Knockdown effects of *Ls_AGO2* and *Ls_DCR2* on virus accumulation in *Laodelphax striatellus.* Knockdown efficiency of *Ls_AGO2* (**A**) and *Ls_DCR2* (**B**). (**C**) Accumulation of RSV abundance in ds-*Ls_AGO2* and ds-*Ls_DCR2* treated insects compared to ds-*GFP* control. (**D**) Accumulation of LSIfV2 abundance in ds-*Ls_AGO2* and ds-*Ls_DCR2* treated insects compared to ds-*GFP* control. Each dot represents a single insect. n ≥ 21. One-way ANOVA was used to determine statistical significance, error bars represent the SEM (**, *p* < 0.01). Virus abbreviation: RSV, Rice stripe virus; LSIfV2, Laodelphax striatellus iflavirus 2.
(TIF)

S6 Fig Effects of key gene knockdown of miRNA and piRNA pathways on viral mRNA and virus-derived small interfering RNAs (vsiRNAs) accumulation in *Laodelphax striatellus.* (**A**) miRNA pathway: Accumulation of vsiRNAs and viral abundance (LSSolV2 (a), LSSolV3 (b), LSIfV2 (c), and LSHV1 (d)) in ds-*Ls_AGO1* and ds-*Ls_DCR1* treated insects compared to ds-*GFP* control. (**B**) piRNA pathway: Accumulation of vsiRNAs and viral abundance (LSSolV2 (a), LSSolV3 (b), LSIfV2 (c), and LSHV1 (d)) in ds-*Ls_Piwi* and ds-*Ls_AGO3* treated insects compared to ds-*GFP* control. RT-qPCR analyses of viral abundance were conducted with three independent biological replicates, each comprising at least 15 insects, whereas small-RNA sequencing was performed with two independent biological replicates per treatment. Two-tailed student’s *t*-test was used to determine statistical significance, error bars represent the SEM (*, 0.01 < *p* < 0.05). Virus abbreviation: LSSolV2, Laodelphax striatellus sobeli-like virus 2; LSSolV3, Laodelphax striatellus sobeli-like virus 3; LSIfV2, Laodelphax striatellus iflavirus 2; LSHV1, Laodelphax striatellus honeydew virus 1.
(TIF)

S7 Fig Effect of double (DCR1 and DCR2) and triple (DCR2, AGO2, and R2D2) knockdown on viral mRNA and vsiRNAs accumulation in *Laodelphax striatellus.* (**A**) Knockdown efficiency of *Ls_DCR1* (a), *Ls_DCR2* (b). (**B**) Knockdown efficiency of *Ls_DCR2* (a), *Ls_AGO2* (b) and *Ls_R2D2* (c). (**C**) Accumulation of vsiRNAs and viral abundance (LSSolV2 (a), *LS*IfV2 (b), and *LS*HV1 (c)) in ds-*Ls_DCR1/DCR2* and ds-*Ls_DCR2/AGO2/R2D2* treated insects compared to ds-*GFP* control. RT-qPCR analyses of knockdown efficiency and viral abundance were conducted with three independent biological replicates, each comprising at least 15 insects, whereas small-RNA sequencing was performed with two independent biological replicates per treatment. Two-tailed student’s *t*-test was used to determine statistical significance, error bars represent the SEM (**, *p* < 0.01). Virus abbreviation: LSSolV2, Laodelphax striatellus sobeli-like virus 2; LSIfV2, Laodelphax striatellus iflavirus 2; LSHV1, Laodelphax striatellus honeydew virus 1.
(TIF)

S8 Fig Small RNA profiles following knockdown of RNAi pathway components in *L. striatellus.* (A) Size distribution of identified miRNAs following *Ls_DCR2* knockdown. (B) Size distribution of identified miRNAs following combined *Ls_DCR2/AGO2/R2D2* knockdown. (C) Size distribution of total clean small RNA reads (18–30 nt) following *Ls_DCR2* knockdown. (D) Size distribution of total clean small RNA reads (18–30 nt) following combined *Ls_DCR2/AGO2/R2D2* knockdown.
(TIF)

S9 Fig Effects of RNAi-mediated suppression on RNAi efficiency in third-instar nymphs of *Laodelphax striatellus.* (**A**) Knockdown efficiency of *Ls_AGO2* (a), *Ls_DCR1* (b) and *Ls_DCR2* (c). (**B**) RNAi efficiency against the reporter gene *Ls_Tubulin* determined after suppression of *Ls_AGO2*, *Ls_DCR1*, or *Ls_DCR2* expression by injection of its respective dsRNA. Each dot represents a single female insect. n ≥ 29. Two-tailed student’s *t*-test was used to determine statistical significance, error bars represent the SEM (**, *p* < 0.01).
(TIF)

S10 Fig Virus expression and development duration in *Laodelphax striatellus* of heterozygous mutation type 1 and heterozygous mutation type 2 compared to wild type control. (**A**) Expression of *Ls_DCR2* in He*MT1* and He*MT2* insects. (**B**) Accumulation of viral (LSHV1) abundance. (**C**) Accumulation of viral (LSIfV2) abundance. (**D**) Accumulation of viral (LSSolV2) abundance. (**E**) Accumulation of viral (LSSolV3) abundance. Each dot represents a single insect. n ≥ 20. (**F**) Development duration in *Laodelphax striatellus* of He*MT1* and He*MT2* compared to wild type control. Two-tailed student’s *t*-test was used to determine statistical significance, error bars represent the SEM (**, *p* < 0.01). Virus abbreviation: LSSolV2, Laodelphax striatellus sobeli-like virus 2; LSIfV2, Laodelphax striatellus iflavirus 2; LSHV1, Laodelphax striatellus honeydew virus 1.
(TIF)

S11 Fig Analysis of insect-specific viruses infection rates in *Laodelphax striatellus* population. A total of 45 insect samples were analyzed. Virus abbreviation: LSSolV2, Laodelphax striatellus sobeli-like virus 2; LSSolV3, Laodelphax striatellus sobeli-like virus 3; HiPV, Himetobi P virus; LSIfV2, Laodelphax striatellus iflavirus 2; LSOlV1, Laodelphax striatellus ollusvirus 1; LSHV1, Laodelphax striatellus honeydew virus 1; LSRV, Laodelphax striatellus reovirus.
(TIF)

S1 Table Statistics of hatched and unhatched eggs laid by female *Laodelphax striatellus* after knockdown of key RNAi pathway genes. (DOCX)

S2 Table Detection of eggs with different genotypes (unhatched eggs) from the G2 population of *Laodelphax striatellus.* (DOCX)

S3 Table Primers used in this study. (DOCX)

S4 Table Insect-specific viruses identified in the lab population of *Laodelphax striatellus.* (DOCX)

S1 Data Numerical data underlying the figures and supporting figures in this study. (XLSX)
