# Supplementary material for: Functional dissection of conserved RNAi pathways in antiviral immunity of several hemipteran insects
Source: PLoS Pathog. 2026 Oct 30;22(10):e1014753. doi: 10.1371/journal.ppat.1014753 (PMC13734579; doi:10.1371/journal.ppat.1014753)
Supplement: Supplementary file 1 [file ppat.1014753.s012.docx]

**Supplemental Table S1. Statistics of hatched and unhatched eggs laid by female *Laodelphax striatellus* after knockdown of key RNAi pathway genes.**

|  | Egg numbers | 1 | 2 | 3 | 4 | 5 | 6 | 7 | 8 | 9 | 10 | 11 | 12 | 13 | 14 | 15 | 16 | 17 | 18 | 19 | 20 | 21 | 22 | 23 | 24 |
| --- | --- | --- | --- | --- | --- | --- | --- | --- | --- | --- | --- | --- | --- | --- | --- | --- | --- | --- | --- | --- | --- | --- | --- | --- | --- |
| ds-*GFP* | Hatched eggs | 20 | 68 | 90 | 36 | 41 | 60 | 107 | 44 | 36 | 67 | 62 | 66 | 45 | 53 | 32 | 59 | 37 | 62 | 66 |  |  |  |  |  |
|  | Unhatched eggs | 2 | 7 | 2 | 4 | 3 | 13 | 2 | 0 | 0 | 2 | 2 | 0 | 2 | 0 | 1 | 0 | 1 | 0 | 4 |  |  |  |  |  |
|  | Total eggs | 22 | 75 | 92 | 40 | 44 | 73 | 109 | 44 | 36 | 69 | 64 | 66 | 47 | 53 | 33 | 59 | 38 | 62 | 70 |  |  |  |  |  |
| ds-*Ls_DCR2* | Hatched eggs | 48 | 82 | 34 | 78 | 45 | 62 | 45 | 59 | 28 | 31 | 28 | 24 | 23 | 52 | 33 | 50 | 19 | 52 | 29 |  |  |  |  |  |
|  | Unhatched eggs | 0 | 0 | 1 | 0 | 0 | 1 | 0 | 0 | 2 | 0 | 0 | 0 | 0 | 1 | 1 | 0 | 0 | 1 | 0 |  |  |  |  |  |
|  | Total eggs | 48 | 82 | 35 | 78 | 45 | 63 | 45 | 59 | 30 | 31 | 28 | 24 | 23 | 53 | 34 | 50 | 19 | 53 | 29 |  |  |  |  |  |
| ds-*Ls_AGO2* | Hatched eggs | 59 | 56 | 29 | 38 | 51 | 81 | 34 | 18 | 34 | 21 | 30 | 68 | 28 | 110 | 76 | 38 | 49 | 64 | 72 | 42 | 54 | 44 | 61 |  |
|  | Unhatched eggs | 0 | 4 | 0 | 3 | 1 | 0 | 0 | 0 | 0 | 2 | 1 | 0 | 0 | 0 | 0 | 0 | 0 | 1 | 0 | 0 | 0 | 0 | 0 |  |
|  | Total eggs | 59 | 60 | 29 | 41 | 52 | 81 | 34 | 18 | 34 | 23 | 31 | 68 | 28 | 110 | 76 | 38 | 49 | 65 | 72 | 42 | 54 | 44 | 61 |  |
| ds-*Ls_R2D2* | Hatched eggs | 30 | 19 | 88 | 103 | 99 | 48 | 53 | 115 | 88 | 29 | 7 | 86 | 49 | 71 | 58 | 19 | 40 | 22 | 63 |  |  |  |  |  |
|  | Unhatched eggs | 1 | 7 | 1 | 2 | 7 | 1 | 1 | 1 | 0 | 0 | 36 | 0 | 0 | 0 | 1 | 0 | 4 | 2 | 1 |  |  |  |  |  |
|  | Total eggs | 31 | 26 | 89 | 105 | 106 | 49 | 54 | 116 | 88 | 29 | 43 | 86 | 49 | 71 | 59 | 19 | 44 | 24 | 64 |  |  |  |  |  |
| ds-*Ls_AGO1* | Hatched eggs | 0 | 0 | 0 | 0 | 0 | 0 | 0 | 0 | 0 | 0 | 0 | 0 | 0 | 0 | 0 | 0 | 0 | 0 | 0 | 0 | 0 | 0 | 0 | 0 |
|  | Unhatched eggs | 0 | 5 | 0 | 0 | 0 | 0 | 0 | 4 | 16 | 0 | 0 | 0 | 0 | 0 | 0 | 6 | 10 | 0 | 0 | 11 | 17 | 16 | 6 | 4 |
|  | Total eggs | 0 | 5 | 0 | 0 | 0 | 0 | 0 | 4 | 16 | 0 | 0 | 0 | 0 | 0 | 0 | 6 | 10 | 0 | 0 | 11 | 17 | 16 | 6 | 4 |
| ds-*Ls_DCR1* | Hatched eggs | 0 | 0 | 0 | 0 | 0 | 0 | 0 | 0 | 0 | 0 | 0 | 0 | 0 | 0 | 0 | 0 | 0 | 0 | 0 | 0 | 0 | 0 | 0 | 0 |
|  | Unhatched eggs | 0 | 0 | 0 | 0 | 0 | 0 | 0 | 0 | 0 | 0 | 0 | 0 | 0 | 0 | 0 | 0 | 0 | 0 | 0 | 0 | 0 | 0 | 0 | 0 |
|  | Total eggs | 0 | 0 | 0 | 0 | 0 | 0 | 0 | 0 | 0 | 0 | 0 | 0 | 0 | 0 | 0 | 0 | 0 | 0 | 0 | 0 | 0 | 0 | 0 | 0 |
| ds-*Ls_Piwi* | Hatched eggs | 0 | 0 | 0 | 0 | 0 | 0 | 0 | 0 | 0 | 0 | 0 | 0 | 0 | 0 | 0 | 0 | 0 | 0 | 0 | 0 | 0 | 0 | 0 | 0 |
|  | Unhatched eggs | 19 | 19 | 11 | 13 | 21 | 5 | 0 | 14 | 6 | 15 | 10 | 5 | 1 | 5 | 0 | 0 | 0 | 0 | 0 | 0 | 0 | 0 | 0 | 0 |
|  | Total eggs | 19 | 19 | 11 | 13 | 21 | 5 | 0 | 14 | 6 | 15 | 10 | 5 | 1 | 5 | 0 | 0 | 0 | 0 | 0 | 0 | 0 | 0 | 0 | 0 |
| ds-*Ls_*AGO3 | Hatched eggs | 11 | 0 | 33 | 0 | 0 | 2 | 1 | 5 | 23 | 0 | 0 | 0 | 44 | 2 | 0 | 12 | 0 | 0 | 0 | 0 | 0 |  |  |  |
|  | Unhatched eggs | 2 | 23 | 44 | 41 | 2 | 19 | 42 | 47 | 21 | 50 | 12 | 3 | 0 | 5 | 60 | 38 | 12 | 25 | 9 | 20 | 25 |  |  |  |
|  | Total eggs | 13 | 23 | 77 | 41 | 2 | 21 | 43 | 52 | 44 | 50 | 12 | 3 | 44 | 7 | 60 | 50 | 12 | 25 | 9 | 20 | 25 |  |  |  |
