# Supplementary material for: Functional dissection of conserved RNAi pathways in antiviral immunity of several hemipteran insects
Source: PLoS Pathog. 2026 Oct 30;22(10):e1014753. doi: 10.1371/journal.ppat.1014753 (PMC13734579; doi:10.1371/journal.ppat.1014753)
Supplement: Supplementary file 2 [file ppat.1014753.s013.docx]

**Supplemental Table S2. Detection of eggs with different genotypes (unhatched eggs) from the G2 population of *Laodelphax striatellus*.**

| Mutation type | Homozygous mutants | Heterozygous mutants | Wild type |
| --- | --- | --- | --- |
| Mutation type 1 | 5/17 | 9/17 | 3/17 |
| Mutation type 2 | 5/28 | 17/28 | 6/28 |
