# Supplementary material for: Functional dissection of conserved RNAi pathways in antiviral immunity of several hemipteran insects
Source: PLoS Pathog. 2026 Oct 30;22(10):e1014753. doi: 10.1371/journal.ppat.1014753 (PMC13734579; doi:10.1371/journal.ppat.1014753)
Supplement: Supplementary file 3 [file ppat.1014753.s014.docx]

**Supplemental Table S3. Primers used in this study.**

| **Primer name** | **Sequence (5' to 3')** |
| --- | --- |
| RNAi-*GFP*-F | 5'-TAATACGACTCACTATAGGGAGTGGAGAGGGTGAAGG-3' |
| RNAi-*GFP*-R | 5'-TAATACGACTCACTATAGGGAGGGCAGATTGTGTGGAC-3' |
| RNAi-*Ls_AGO1*-F | 5'-TAATACGACTCACTATAGGGAGAATTGACTGACTCCCAACGGG-3' |
| RNAi-*Ls_AGO1*-R | 5'-TAATACGACTCACTATAGGGAGATGTCCGACCTGAAGACAAGG-3' |
| RNAi-*Ls_DCR1*-F | 5'-TAATACGACTCACTATAGGGAGAACCAATCGGCTCACTGACTG-3' |
| RNAi-*Ls_DCR1*-R | 5'-TAATACGACTCACTATAGGGAGACCGAATTTGGCGGTTTCTGG-3' |
| RNAi-*Ls_Piwi*-F | 5'-TAATACGACTCACTATAGGGAGATCGCACACGAATGGACTCAA-3' |
| RNAi-*Ls_Piwi*-R | 5'-TAATACGACTCACTATAGGGAGATCACCACGTTAGGGTGTTCG-3' |
| RNAi-*Ls_AGO3*-F | 5'-TAATACGACTCACTATAGGGAGAACATCGCTGATTGGAAGCGA-3' |
| RNAi-*Ls_AGO3*-R | 5'-TAATACGACTCACTATAGGGAGACCGCGAACGGAATTTTGACA-3' |
| RNAi-*Ls_AGO2*-F | 5'-TAATACGACTCACTATAGGGAGAACCGAACTGATACCAAACGTACT-3' |
| RNAi-*Ls_AGO2*-R | 5'-TAATACGACTCACTATAGGGAGATTCTGTGGGCCAATCCAGAC-3' |
| RNAi-*Ls_DCR2*-F | 5'-TAATACGACTCACTATAGGGAGATTCTCGGATGTCTTGCAGTG-3' |
| RNAi-*Ls_DCR2*-R | 5'-TAATACGACTCACTATAGGGAGATCGTCGGCTAGAAGCAGATA-3' |
| RNAi-*Ls_R2D2*-F | 5'-TAATACGACTCACTATAGGGAGAATTGGCAGTCACAACCCTGT-3' |
| RNAi-*Ls_R2D2*-R | 5'-TAATACGACTCACTATAGGGAGAGATTTGTGCTTGGCCTGCTT-3' |
| RNAi-*Ls_Tubulin*-F | 5'-TAATACGACTCACTATAGGGAGAGCGCGAATGTATCTCAGTGC-3' |
| RNAi-*Ls_Tubulin*-R | 5'-TAATACGACTCACTATAGGGAGATCCAGTACCAGGTCGACGAT-3' |
| RNAi-*Sf_AGO2*-F | 5'-TAATACGACTCACTATAGGGAGATTGTTGGCGTGGGAAGATCA-3' |
| RNAi-*Sf_AGO2*-R | 5'-TAATACGACTCACTATAGGGAGACGGAGTCAACTTCCGGTTCA-3' |
| RNAi-*Sf_DCR2*-F | 5'-TAATACGACTCACTATAGGGAGATCAAGTACGCGGTGTCACTC-3' |
| RNAi-*Sf_DCR2*-R | 5'-TAATACGACTCACTATAGGGAGATCTCCATGTAGACGTCGGGT-3' |
| RNAi-*Nl_AGO2*-F | 5'-TAATACGACTCACTATAGGGAGATCCCACCCAACAAACCGAAT-3' |
| RNAi-*Nl_AGO2*-R | 5'-TAATACGACTCACTATAGGGAGAGATGGTTTCGGGCTTGCATC-3' |
| RNAi-*Nl_DCR2*-F | 5'-TAATACGACTCACTATAGGGAGAGTGGATCGTACGGTGCAGAA-3' |
| RNAi-*Nl_DCR2*-R | 5'-TAATACGACTCACTATAGGGAGAGGTGTCAGGTTGTCGCAGTA-3' |
| RNAi-*Bt_AGO2*-F | 5'-TAATACGACTCACTATAGGGAGAGTCGACGTTGCTCACAAAGG-3' |
| RNAi-*Bt_AGO2*-R | 5'-TAATACGACTCACTATAGGGAGACTGTGGCCTCGGATTGGATT-3' |
| RNAi-*Bt_AGO2*-2F | 5'-TAATACGACTCACTATAGGGAGATTGTCATACCTGGTCACGGC-3' |
| RNAi-*Bt_AGO2*-2R | 5'-TAATACGACTCACTATAGGGAGAGCATTCAATTCCGCAGCGAG-3' |
| RNAi-*Bt_DCR2*-F | 5'-TAATACGACTCACTATAGGGAGAGTCCGATCAGCCTGTGTGAA-3' |
| RNAi-*Bt_DCR2*-R | 5'-TAATACGACTCACTATAGGGAGAATTTGTTGCGCCCAAAACCA-3' |
| RNAi-*Bt_DCR2*-2F | 5'-TAATACGACTCACTATAGGGAGATGTCCCGAAATGGGACCAAG-3' |
| RNAi-*Bt_DCR2*-2R | 5'-TAATACGACTCACTATAGGGAGAGAGCTAGAGCCTCTTGGCAG-3' |
| sgRNA-Ls_DCR2-1 | 5'- TAATACGACTCACTATAGCCTCACGTGGTGGGGTTGACGTTTTAGAGCTAGAAATAGC -3' |
| sgRNA-Ls_DCR2-2 | 5'-TAATACGACTCACTATAGCACTTCTGTCAACGAAAGCAGTTTTAGAGCTAGAAATAGC-3' |
| sgRNA-R | 5'-AAAAAAAGCACCGACTCGGTGCCAC-3' |
| LSSolV2-F | 5'-CGACATGACAGCTTGGGACT-3' |
| LSSolV2-R | 5'-TAACCAACGCAGGACTCACC-3' |
| LSSolV3-F | 5'-TCGAGGAGTTTTCGGGGTTG-3' |
| LSSolV3-R | 5'-CGACTCTACGACTGACTCGC-3' |
| HiPV-F | 5'-TGTTGCTTTAGCAGGCCGTA-3' |
| HiPV-R | 5'-ACTCTGGCCCTTTTCCGAAG-3' |
| LSIfV2-F | 5'-TTATGCGTCCATGCCAGGTT-3' |
| LSIfV2-R | 5'-GCAAGCTGTATTTCCGTCGC-3' |
| LSOlV1-F | 5'-TGAGGGCCATGCAAGGTATG-3' |
| LSOlV1-R | 5'-CCCCCGCACTTAAAGATGGT-3' |
| LSHV1-F | 5'-GCCCGCTTAGATCTTGGACA-3' |
| LSHV1-R | 5'-AATGATGCGTACTCGGTGCC-3' |
| LSRV-F | 5'-AACACAGTCTGCTTGGTGGT-3' |
| LSRV-R | 5'-TCGCGTCATCAGTTCGACAT-3' |
| q-*Ls_actin*-F | 5'-AGTGCCCATCTACGAAGGTTACG-3' |
| q-*Ls_actin*-R | 5'-CGGCGGTGGTGGTGAAGC-3' |
| q-*Ls_Tubulin*-F | 5'-GGATTCCTCATCTTCCACTC-3' |
| q-*Ls_Tubulin*-R | 5'-GATAGCCTCATTGTCTACCAT-3' |
| q-RSV-CP-F | 5'-TGCAGAAGGCAATCAATGACAT-3' |
| q-RSV-CP-R | 5'-TGTCACCACCTTTGTCCTTCAA-3' |
| q-*Ls_AGO1*-F | 5'-CCTAGAAGGCCAAACCTCGG-3' |
| q-*Ls_AGO1*-R | 5'-ATCTCCCGGTTGACTTTGCG-3' |
| q-*Ls_DCR1*-F | 5'-GGGGAGCTTTCATGCTGGAT-3' |
| q-*Ls_DCR1*-R | 5'-GACGCATAGCTGTCCCCTTC-3' |
| q-*Ls_*Piwi-F | 5'-CTTGTGAGGACCAAGCCTGA-3' |
| q-*Ls_*Piwi-R | 5'-AACAGTCCGCGCTTTATTGC-3' |
| q-*Ls_AGO3*-F | 5'-TTGGATGCAGTCAGTAGGCT-3' |
| q-*Ls_AGO3*-R | 5'-CCGGGATCGATACAGCTTCC-3' |
| q-*Ls_AGO2*-F | 5'-TAAACCGGACAAGCCTCGAC-3' |
| q-*Ls_AGO2*-R | 5'-CTGCCGTTCGCTGTTGAAAA-3' |
| q-*Ls_DCR2*-F | 5'-CTGAAAGCGTGCAAACTGCT-3' |
| q-*Ls_DCR2*-R | 5'-CACTTTTTGGTGCCGACAGG-3' |
| q-*Ls_R2D2*-F | 5'-ATCGAAGAAGAGCTCGACGG-3' |
| q-*Ls_R2D2*-R | 5'-ACTCGGAAATGTTCACTCCAT-3' |
| q-LSSolV2-F | 5'-AGGATATGGAGTACGGAACA-3' |
| q-LSSolV2-R | 5'-AAGGCCCTATAATACGCTTG-3' |
| q-LSSolV3-F | 5'-GGGTGGAGGTCAGGTCAG-3' |
| q-LSSolV3-R | 5'-CCGCTCATCGTCGTCAAG-3' |
| q-LSIfV2-F | 5'-ATTCGTGTCGCGTGGTTAGA-3' |
| q-LSIfV2-R | 5'-GTGGCAGTACTAAGAGAGCGA-3' |
| q-LSHV1-F | 5'-AATACTGCTTGCTTGCGTGC-3' |
| q-LSHV1-R | 5'-AGGGAGACCTGCTAGGACAG-3' |
| q-LSRV-F | 5'-GCACTCTTACCCACCCTGTC-3' |
| q-LSRV-R | 5'-TCGACCACGCACGAAGTAAT-3' |
| q-*Sf_actin*-F | 5'-CACACCTTCTACAACGAGTT-3' |
| q-*Sf_actin*-R | 5'-GAGCATAACCTTCATAGATGG-3' |
| q-*Sf_AGO2*-F | 5'-AGTGACGGTGAAGTTCGCTT-3' |
| q-*Sf_AGO2*-R | 5'-TGATCTTCCCACGCCAACAA-3' |
| q-*Sf_DCR2*-F | 5'-TTGTCCCACCAACGGTTGAA-3' |
| q-*Sf_DCR2*-R | 5'-CTTCCAGCAAGGGCTTTTCG-3' |
| q-SfIfV1-F | 5'-TGGCATGAGCGTGTACCTTT-3' |
| q-SfIfV1-R | 5'-CCTCGACACTGTCCAAACGA-3' |
| q-SfHV-F | 5'-CTTGGCGCTGGCGATATAGA-3' |
| q-SfHV-R | 5'-ACACGCACTTGCAAATCACC-3' |
| q-*Nl_actin*-F | 5'-GTGCGTGACATCAAGGAGAAGC-3' |
| q-*Nl_actin*-R | 5'-GGAAGGAAGGCTGGAACAGAG-3' |
| q-*Nl_AGO2*-F | 5'-ATGGTGAGGAATGCAGCCAA-3' |
| q-*Nl_AGO2*-R | 5'-CGTCGGTTTGATCTGTTGCC-3' |
| q-*Nl_DCR2*-F | 5'-GCACTGGTGAACAACGTGAC-3' |
| q-*Nl_DCR2*-R | 5'-GAACCTCAATGGACTCGCCA-3' |
| q-NlBunya1-F | 5'-AAGCTATGGGGCTCGAGGTA-3' |
| q-NlBunya1-R | 5'-CCCCATTCCGGTATGCTTGT-3' |
| q-NlBunya2-F | 5'-GGCTCGTCACAGGAGTTCAA-3' |
| q-NlBunya2-R | 5'-TTCACCTTTTGGTGGGCAGT-3' |
| q-NlBunya3-F | 5'-GACGCACACAACCAAGTGTC-3' |
| q-NlBunya3-R | 5'-TTACTGCCAGCTCGAAAGCA-3' |
| q-NlBunya4-F | 5'-CACGGTGTGTCACCATGAGA-3' |
| q-NlBunya4-R | 5'-GACTGTTGAAACCACGGCAC-3' |
| q-*Bt_actin*-F  q-*Bt_actin*-R | 5'-TCTTCCAGCCATCCTTCTTG-3'  5'-CGGTGATTTCCTTCTGCATT-3' |
| q-*Bt_AGO2*-F | 5'-CGCCGTTAGTTTCTGGTCCT-3' |
| q-*Bt_AGO2*-R | 5'-GGAAGCCTTTGTGAGCAACG-3' |
| q-*Bt_DCR2*-F | 5'-TCTGCGAGTGGGAGAGTACA-3' |
| q-*Bt_DCR2*-R | 5'-CTGAACAGCTCGGTGACACT-3' |
| q-BtViLV1-F | 5'-ATGGACCGCTCAAGAAGTGG-3' |
| q-BtViLV1-R | 5'-ATTCGAACGTCTCTCGGTCG-3' |
| q-BtViLV2-F | 5'-ACGAGGCCCGAAATAAGCTC-3' |
| q-BtViLV2-R | 5'-AAGGCGACTTTGGAGTCGTT-3' |
| q-BtPiLV2-F | 5'-AGGTGAGTATTGCACGCGAA-3' |
| q-BtPiLV2-R | 5'-GTCGACGACAACAGGGTCTT-3' |
