# Supplementary material for: Functional dissection of conserved RNAi pathways in antiviral immunity of several hemipteran insects
Source: PLoS Pathog. 2026 Oct 30;22(10):e1014753. doi: 10.1371/journal.ppat.1014753 (PMC13734579; doi:10.1371/journal.ppat.1014753)
Supplement: Supplementary file 4 [file ppat.1014753.s015.docx]

**Supplemental Table S4. Insect-specific viruses identified in the lab population of *Laodelphax striatellus.***

| Abbreviation | Full name | Accession | Family |
| --- | --- | --- | --- |
| HiPV | Himetobi P virus | KF280585.1 | *Dicistroviridae* |
| LSIfV2 | Laodelphax striatellus iflavirus 2 | PP781946 | *Iflaviridae* |
| LSHV1 | Laodelphax striatellus honeydew virus 1 | NC_023627.1 | *Iflaviridae* |
| LSRV | Laodelphax striatellus reovirus | S1: OM249649 | *Spinareoviridae* |
|  |  | S2: OM249650 |  |
|  |  | S3: OM249651 |  |
|  |  | S4: OM249652 |  |
|  |  | S5: OM249653 |  |
|  |  | S6: OM249654 |  |
|  |  | S7: OM249655 |  |
|  |  | S8: OM249656 |  |
|  |  | S9: OM249657 |  |
|  |  | S10: OM249658 |  |
| LSSolV2 | Laodelphax striatellus sobeli-like virus 2 | BK068174 | Unassigned |
| LSSolV3 | Laodelphax striatellus sobeli-like virus 3 | BK068175 | Unassigned |
| LsOlV1 | Laodelphax striatellus ollusvirus 1 | BK068182 | *Aliusviridae* |
