# Supplementary material for: Functional dissection of conserved RNAi pathways in antiviral immunity of several hemipteran insects
Source: PLoS Pathog. 2026 Oct 30;22(10):e1014753. doi: 10.1371/journal.ppat.1014753 (PMC13734579; doi:10.1371/journal.ppat.1014753)
Supplement: Supplementary file 5 [file ppat.1014753.s017.docx]

**Response to Editor and Reviewers**

**Manuscript (PPATHOGENS-D-25-02414)**

***“Functional dissection of conserved RNAi pathways reveals Dicer2-independent antiviral defense against RNA viruses in*** ***several hemipteran insects”***

We sincerely thank the three Reviewers for their thorough evaluation of our manuscript and their constructive comments. These comments have substantially improved both the quality and clarity of the current work. In the revised manuscript, all the major and minor concerns have been carefully addressed with newly performed experiments. Below, we have responded to each comment raised by reviewers and editor. Our responses are shown in blue. Changes in the revised manuscript are indicated by line numbers.

**Reviewers' Comments:**

**Part I - Summary**

Please use this section to discuss strengths/weaknesses of study, novelty/significance, general execution and scholarship.

**Reviewer #1:**

This manuscript investigates the three canonical insect RNAi pathways in the hemipteran *L. striatellus* and related species. The authors report that antiviral control of several insect-specific RNA viruses depends on AGO2 but is independent of DCL2. CRISPR/Cas9 knockout of DCL2 results in embryonic lethality, while heterozygous mutants exhibit delayed development. These findings lead the authors to conclude that in Hemiptera, DCL2 is essential for development but dispensable for antiviral RNAi. The study is conceptually significant because it challenges the canonical model of DCL2 mediated siRNA biogenesis established in Diptera. However, several mechanistic and data transparency issues must be addressed before the manuscript can be considered for publication.

**Response:** We thank Reviewer #1 for the positive assessment of the conceptual significance of this work and for the constructive and detailed suggestions. In response, we have performed additional experiments - including an RNAi-of-RNAi assay, double/triple knockdown experiments, and biochemical protein expression and cleavage assays - to further elucidate the mechanism underlying DCR2-independent antiviral siRNA production. We have also substantially improved data transparency throughout, as described in detail in response to each specific comment below. All raw sequencing data have been deposited in NCBI (BioProject: PRJNA1405795).

**Reviewer #2:**

The research manuscript ‘Functional dissection of conserved RNAi pathways reveals Dicer2-independent antiviral defense against RNA viruses in hemipteran insects’ by Zhang et al. characterizes small silencing RNA pathways in the hemipteran insect Laodelphax striatellus. The authors identify orthologous genes at the heart of the microRNA, piRNA and siRNA pathways and, upon depletion of these core factors, they analyze physiological traits including fecundity and fertility, small RNA populations, and virus replication. In line with literature from other insect models, they demonstrate the microRNA pathway to be important for survival and developmental phenotypes. Likewise, knockdown of Piwi, a central piRNA binding protein in the piRNA pathway results in reduced fertility. Knockdown of several, but not all, small silencing RNA pathway-associated proteins results in decrease of the corresponding class of small RNA. Antiviral activity was demonstrated to be associated with the siRNA pathway only. Strikingly, the authors propose that the antiviral siRNA pathway in Laodelphax striatellus and two additional non-model insect species acts independently of Dicer-2, a core component of the siRNA machinery. An attempt to validate this significant finding using a full genetic knockout unfortunately results in lethality of homozygous mutants.

The descriptive part of the study is mostly solid and the data on fecundity and fertility upon knockdown of individual proteins form the distinct small silencing RNA pathway knockdown is largely coherent with findings in other insect models. Mechanistically, the study remains superficial, especially on the characterization of microRNA and piRNA populations. These small RNA classes could have been analyzed in greater depth for example by quantifying individual microRNAs or by checking the piRNA pool for the commonly seen nucleotide biases.

**Response:** We thank the reviewer for this suggestion. We respectfully note, however, that in-depth characterization of miRNA and piRNA population dynamics falls outside the primary scope of the present study. Our central objective was to functionally determine whether perturbation of each of the three small silencing RNA pathways affects antiviral defense against RNA viruses in *L. striatellus*. Total miRNA and piRNA abundance were therefore used as functional readouts to confirm effective pathway disruption upon gene knockdown to address our core question. We fully agree that quantification of individual miRNA species and analysis of characteristic piRNA nucleotide biases (such as 1U and 10A signatures) would be highly informative and represent important directions for future dedicated studies. An ongoing study focusing on the population dynamics of miRNAs and piRNAs in hemipteran insects is currently being planned in our laboratory, and the data generated in the present study will serve as a valuable foundation for those analyses.

The observation of Dicer-2 independent siRNA production is a significant claim and the burden of proof for this observation is high. In its current state, the study does not provide convincingly firm evidence to support this conclusion. As the authors have noted, RNAi-mediated knockdown of the RNAi machinery is inherently tricky and has resulted in sub-functional protein depletion in other models. With the data presented, the authors cannot exclude that residual Dicer-2 protein activity is able to generate siRNAs in sufficient numbers to guide Ago2 to silence viral transcripts. In fact, siRNA populations in wildtype planthoppers do present the well-described features of siRNA populations in terms of size and similar number of reads from positive and negative strands. To me this strongly suggests that a Dicer protein is responsible for the siRNA production. I appreciate the attempt of the authors to address this important aspect with a genetic knockout model, but as these data are still inconclusive due to lethality, the manuscript lacks the final proof for Dicer-2 independent siRNA biogenesis.

**Response:** We fully appreciate that this is the most critical concern in this manuscript, and we have undertaken three complementary experimental approaches in the revised manuscript to address it.

First, to address the concern that residual DCR2 protein following knockdown may sustain siRNA production, we performed an RNAi-of-RNAi assay in *L. striatellus*. Third-instar nymphs were pre-injected with dsRNA targeting *Ls_DCR1*, *Ls_DCR2*, or *Ls_AGO2*, followed 48 hours later by injection of *ds-Ls_Tubulin* as a reporter of RNAi pathway activity. Only pre-knockdown of *Ls_AGO2* significantly impaired reporter cleavage efficiency; pre-knockdown of neither *Ls_DCR1* nor *Ls_DCR2* had any detectable effect (Supplementary Figure S7; revised lines 303-314 and 446-449). While pre-knockdown of DCR2 significantly affects RNAi efficiency in various insects of the orders Orthoptera, Coleoptera, and Lepidoptera (refer to the newly added references of number 69-73, lines 844-859 and 449-458), this result demonstrates that DCR2 enzymatic activity is not required for dsRNA processing in *L. striatellus*.

Second, to exclude functional redundancy, we performed double and triple knockdown experiments by simultaneously injecting dsRNAs targeting *Ls_DCR1*/*Ls_DCR2* and *Ls_DCR2*/*Ls_AGO2*/*Ls_R2D2* in third-instar insects. The results demonstrated that there is no functional redundancy between DCR1 and DCR2 in *L. striatellus*. (Supplementary Figure S6; revised lines 290-302).

Third, CRISPR/Cas9-generated heterozygous mutants carrying significantly reduced DCR2 expression showed no significant change in viral accumulation relative to wild-type insects (Supplementary Figure S8; revised lines 338-341), providing genetically independent supporting evidence.

We acknowledge, as the reviewer rightly observes, that the well-defined size profiles and balanced strand representation of vsiRNAs in wild-type planthoppers strongly suggest involvement of a Dicer-family enzyme in their biogenesis. Our data point to the existence of a currently unidentified alternative nuclease in hemipteran insects responsible for vsiRNA biogenesis. This interpretation and its implications have been discussed in the revised Discussion section (revised lines 436-445).

N.B. The same argument can be made for the absence of a fertility/piRNA phenotype upon Ago3 knockdown which can still be attributed to residual protein activity. Therefore, the claim that ‘Ls_Piwi, but not Ls_AGO3, is actively involved in the piRNA pathway of L. striatellus’ (lines 246-7) still requires more rigorous experimental validation.

**Response:** We thank the reviewer for this important note. We fully agree that the absence of a detectable piRNA reduction or fertility phenotype upon *Ls_AGO3* knockdown cannot by itself exclude a role for AGO3 in the piRNA pathway, which might be due to incomplete protein depletion. Furthermore, if ping-pong amplification activity is limited in *L. striatellus*, a strong AGO3-dependent piRNA phenotype would not necessarily be expected even with efficient knockdown. We have therefore revised the relevant statements to present this conclusion more cautiously, removing the claim that AGO3 is not involved in the piRNA pathway and replacing it with a more measured interpretation acknowledging this limitation (revised lines 252-256 and 416-420).

**Reviewer #3:**

In this manuscript, the authors analyze the role of small RNA pathways in antiviral defense in hemipteran insects. Through a knockdown approach, they find that knockdown of the miRNA (Ago1 and Dcl1) and piRNA (Piwi and Ago3) pathway genes affect fecundity and development rates. Knockdown of Ago2 led to an increase in viral replication and consequently an increase in viral siRNA numbers. Surprisingly, knockdown of Dcl2 did not affect siRNA levels and viral replication. This observation is the basis for the main conclusion of the title of Dicer 2 (DCL2) independent antiviral defense. Next, the authors generated a Dicer2 knockout using CRISPR/Cas9 and found that this led to a developmental arrest and no adult homozygotes were obtained. Finally, the authors performed Ago2 and DCL2 knockdowns in other insects and again find that Ago2 affects viral replication whereas DCL2 does not.

Overall, the manuscript supports the critical role of RNAi in antiviral defense and suggests a role of siRNA pathway in development in Laodelphax striatellus – the small brown planthopper, a vector for several plant viruses. These findings are in line with findings in other organisms and previous reports of in this species (https://doi.org/10.1186/s12870-018-1438-7; https://doi.org/10.3390/v13081591), and therefore not unexpected. The title suggests that the authors consider the conclusion that antiviral RNAi is independent of DCL2 the main novelty of the manuscript, but in my opinion this conclusion is not warranted based on the provided data (see below).

**Response:** We thank Reviewer #3 for the summary of our work and for the constructive comments. We respectfully note that while antiviral RNAi activity in *L. striatellus* has been reported previously, the DCR2-independent nature of this activity has not been demonstrated before and represents the central novelty of the present study. Regarding the concern that the conclusion is not fully warranted by the data, in the revised manuscript, we have addressed these concerns through multiple complementary experimental approaches. In particular, the newly performed RNAi-of-RNAi assay provides functional evidence that DCR2 does not participate in dsRNA processing in *L. striatellus*. Combined with the additional double/triple knockdown experiment and the CRISPR heterozygous mutant data, we believe the revised manuscript now presents a substantially strengthened case for DCR2-independent antiviral immunity in hemipteran insect (*L. striatellus*). We have also carefully qualified our conclusions where appropriate throughout the text in the revised manuscript and address this in detail in response to the specific major issues raised below.

**Part II – Major Issues: Key Experiments Required for Acceptance**

Please use this section to detail the key new experiments or modifications of existing experiments that should be absolutely required to validate study conclusions.

**Reviewer #1:**

1. The mechanistic evidence supporting DCL2 independent vsiRNA biogenesis remains incomplete. The authors may consider expressing L. striatellus DCL2 and DCL1 in vitro, purifying the recombinant proteins, and performing dsRNA cleavage assays. Comparing the enzymatic activities of these RNase III–like proteins would provide direct biochemical evidence of their functions and clarify whether DCL2 is indeed dispensable for vsiRNA production in this species.

**Response:** We thank the reviewer for this constructive suggestion and for encouraging a direct biochemical approach. We have attempted *in vitro* dsRNA cleavage assays using recombinant proteins of Ls-DCR1, Ls-DCR2, and Ls-AGO2 (expressed in *E. coli*), following the protocol described by Gao et al. (2022) for *Locusta migratoria* Dicer proteins (newly added reference 69 in the revised manuscript, doi: 10.1016/j.ibmb.2022.103865). Specifically, we expressed the RNase IIIb domain and dsRNA-binding domain (dsRBD) of Ls-DCR1 and Ls-DCR2 as recombinant fusion proteins (designated LsDCR1-RR and LsDCR2-RR, respectively), as well as Ls-AGO2. Reaction mixtures (20 μL total volume) contained 4 μg *dsLs-Tubulin* as substrate and 2 μL of 10× cleavage buffer [0.2 M Tris-HCl, 5 mM EDTA, 50 mM MgCl₂, 10 mM DTT, 1.4 M NaCl, 27 mM KCl (pH 7.9)]. As shown in the figure below, assays were performed across a range of protein concentrations (2.5-15 μM, Panel A) and incubation times (2.5-30 min at 37°C, Panel B), and reaction products were resolved on 2% (w/v) agarose gels. The results indicated that, under all tested conditions (across both concentration gradients and time courses), none of the three recombinant proteins produced detectable sRNA cleavage products from the *dsLs-Tubulin* substrate. The full-length substrate band remained intact, and no signal at approximately 50 bp (indicative of siRNA-sized products) was observed for LsDCR1-RR, LsDCR2-RR, or LsAGO2.

Unfortunately, the result indicated that all of the three proteins (LsDCR1-RR, LsDCR2-RR, and Ls-AGO2) cannot produce detectable siRNA-sized cleavage products under any tested condition. The full-length substrate band remained intact throughout, with no signal at approximately 50 bp observable in either case (figure below). We note that this outcome, while inconclusive, is not unexpected: reconstituting Dicer enzymatic activity from truncated bacterial-expressed domains is technically demanding, and the absence of the full-length protein architecture, accessory domains, and in vivo cofactors likely prevents catalytic activity in this system. Critically, because neither protein produced cleavage products, we therefore do not interpret it as evidence for DCR2-independent siRNA biogenesis and these data was not included in the revised manuscript.

Instead, we have pursued three complementary functional approaches that provide more reliable and directly interpretable evidence: (1) an RNAi-of-RNAi assay in which pre-knockdown of *Ls_DCR2* had no significant effect on the cleavage efficiency of a subsequently injected dsRNA reporter substrate, demonstrating that DCR2 enzymatic activity is not required for dsRNA processing in *L. striatellus* under functional in vivo conditions (Supplementary Figure S7; lines 303-314 and 446-458); (2) double and triple knockdown experiments showing that the antiviral phenotype is attributable specifically to *AGO2* depletion, not to *DCR2* (Supplementary Figure S6; lines 290-302); and (3) CRISPR/Cas9-generated heterozygous *Ls_DCR2* mutants carrying significantly reduced *DCR2* expression show no change in viral accumulation relative to wild-type controls (Supplementary Figure S8; lines 338-341). Taken together, these independent and convergent lines of functional evidence support the conclusion that DCR2 is not the primary driver of antiviral siRNA production in *L. striatellus*.

2. The observation that AGO2 knockdown increases both viral RNA and vsiRNA counts is expected if higher viral replication produces more precursor dsRNA. This phenomenon by itself does not reveal the nuclease responsible for vsiRNA generation. The authors should normalize vsiRNA abundance to viral genome copy numbers and analyze vsiRNA mapping profiles to distinguish genuine Dicer processed products from nonspecific degradation fragments, thereby providing stronger mechanistic support for their conclusion.

**Response:** We sincerely thank the reviewer for this important point, and we apologize for not addressing it adequately. Firstly, we apologize that we have omitted the detailed method of vsiRNA analysis from the original submission. We have added to the revised Methods section the description of RPM (reads per million total mapped reads) normalization which is a standard and widely used approach for quantifying vsiRNA abundance in insect antiviral RNAi studies (lines 586-598). We fully agree that an increase in both viral RNA and vsiRNA abundance following AGO2 knockdown could simply reflect greater availability of viral dsRNA precursor due to enhanced viral replication. We recognize, however, that RPM normalization corrects for sequencing depth between libraries rather than for differences in viral RNA abundance between treatment groups. Nevertheless, we have two lines of solid evidence to support our conclusion. First, the vsiRNA size distribution profiles and strand polarity patterns in DCR2-knockdown insects are indistinguishable from those of ds-*GFP* controls (Figure 4), with canonical 21/22-nt peaks and balanced strand representation fully maintained. The preservation of these hallmark features of Dicer-processed siRNAs, even after DCR2 knockdown, suggests that the responsible processing enzyme remains active irrespective of DCR2 levels. Second, the RNAi-of-RNAi assay (Supplementary Figure S7) uses a non-replicating reporter dsRNA (*Ls_Tubulin*) injected at a defined dose, entirely bypassing the confound of replication-driven precursor accumulation. The finding that DCR2 pre-knockdown does not impair reporter cleavage efficiency provides functional evidence for DCR2 dispensability that is independent of viral RNA abundance. We acknowledge that formal normalization of vsiRNA to viral genome copy number would strengthen the mechanistic interpretation and have noted this as an important direction for future work in the revised Discussion (lines 436-445).

3. The manuscript currently lacks compliant data deposition information. According to PLOS Pathogens policy, all sequencing data supporting the findings must be deposited in an appropriate public repository, and accession numbers must be clearly provided in the data availability statement. The present statement that all data are available in the main text or Supplementary Materials does not meet this requirement. The authors should ensure that all raw and processed datasets are publicly accessible and that repository accession numbers are included before resubmission.

**Response:** We thank the reviewer for pointing out this important requirement. All raw and processed sequencing datasets generated in this study have now been deposited in the NCBI Sequence Read Archive (BioProject Accession: PRJNA1405795) (lines 637-640).

4. Several figures appear to rely on unadjusted two-tailed Student’s t-tests across multiple comparisons. The authors should specify whether each dataset represents biological or technical replicates, provide the exact sample size (n), test assumptions of normality and variance, and apply appropriate multiple comparison corrections where necessary.

**Response:** We thank the reviewer for this important concern regarding statistical issues. In the revised manuscript, we have taken the following steps throughout: (1) all figure legends now explicitly state whether data points represent independent biological replicates or individual insects, and include exact sample size (n) values; (2) normality was assessed for all datasets, and where data were not normally distributed (such as Figure S4), analyses were repeated with non-parametric tests or one-way ANOVA with Tukey’s correction as appropriate; (3) survival curves are now analyzed using the Log-rank (Mantel-Cox) test in place of t-tests (lines 912-914); and (4) error bars are explicitly defined as SEM in all figure legends.

**Reviewer #2**

The central claim, included in the title, that (selected) hemipteran insects produce siRNAs in a Dicer-2 independent fashion requires firm experimental proof. For this purpose, a model with validated functional knockout of dicer-2 activity must be achieved. While I recognize that this will be difficult to obtain, given the data presented here, this remains the ultimate experimental evidence to support this remarkable conclusion. If these data cannot be generated, the conclusions should be toned down significantly, in which case much of the general interest and novelty of this manuscript is reduced.

Another way forward would be identifying the proposed unknown nuclease, other than Dicer-2, that the authors suspect to be involved in siRNA biogenesis. Given the siRNA characteristics, Dicer-1 might be a logical candidate. In this line of reasoning, I was wondering if the authors have considered the possibility of redundant functions of Dicer 1 and Dicer 2 that could mask the siRNA phenotype of Dicer-2 knockdown. This could for example be tested by co-injecting dsRNA targeting both Dicer proteins at the same time.

**Response:** We deeply appreciate Reviewer #2’s concern of the evidential standard required for this claim. A validated functional knockout of DCR2 would indeed represent the most definitive proof. We genuinely attempted to generate this model: our CRISPR/Cas9-mediated knockout of Ls_DCR2 produced two independent mutant lines, but homozygous mutants exhibit embryonic lethality, preventing direct assessment of antiviral immunity in adult insects. This outcome itself reveals an important and unexpected biological finding that DCR2 plays an essential developmental role in *L. striatellus*. However, it regrettably precludes the use of homozygous knockout insects for antiviral assays.

In the absence of a viable homozygous knockout, we have pursued every currently feasible alternative approach to provide additional evidence. We respectfully submit that the following four independent and complementary lines of evidence, taken together, also provide compelling support for the conclusion that antiviral siRNA production in *L. striatellus* operates independently of DCR2.

(1) Double knockdown of *Ls_DCR1* and *Ls_DCR2*.

To test the possibility of functional redundancy between the two Dicer proteins as suggested by Reviewer #2, we co-injected dsRNAs targeting both *Ls_DCR1* and *Ls_DCR2* simultaneously. Even under dual knockdown conditions, viral RNA accumulation and vsiRNA profiles remained indistinguishable from *ds-GFP* controls (Supplementary Figure S6). This result rules out the possibility of DCR1/DCR2 redundancy.

(2) Triple knockdown of *Ls_DCR2*, *Ls_AGO2*, and *Ls_R2D2*.

Simultaneous knockdown of all three canonical siRNA pathway components revealed that only the AGO2-containing condition produced a significant increase in viral abundance and vsiRNA accumulation. Neither DCR2 nor R2D2 knockdown recapitulated the AGO2 phenotype (Supplementary Figure S6). This further supports the conclusion that DCR2 is dispensable for antiviral siRNA function in this species.

(3) RNAi-of-RNAi assay.

To address the concern that residual DCR2 protein following knockdown may be sufficient to sustain siRNA production, we employed a functional assay that measures RNAi pathway output rather than transcript depletion alone. Third-instar nymphs were pre-injected with dsRNAs targeting *Ls_DCR1*, *Ls_DCR2*, or *Ls_AGO2*, followed by injection of *ds-Ls_Tubulin* as a pathway reporter. Pre-knockdown of *Ls_AGO2* caused a 76.5% reduction in reporter cleavage efficiency, while pre-knockdown of *Ls_DCR1* or *Ls_DCR2* produced no significant effect (Supplementary Figure S7; lines 298-309 and 441-444). Importantly, this assay is independent of viral replication dynamics and uses a defined-dose non-replicating substrate, eliminating the confound of precursor accumulation. Moreover, the same RNAi-of-RNAi approach has previously been applied in insects from multiple other orders, including *Tribolium castaneum* (Coleoptera), *Leptinotarsa decemlineata* (Coleoptera), *Schistocerca gregaria* (Orthoptera), *Locusta migratoria* (Orthoptera), and *Chilo suppressalis* (Lepidoptera), and consistently identifies DCR2 as a functional contributor to the siRNA pathway (newly added references from 69-73).

(4) CRISPR/Cas9 heterozygous mutant analysis.

Although homozygous DCR2 knockout insects are not viable, DCR2-heterozygous mutants (both MT1 and MT2 lines) carry one disrupted allele and show significantly reduced DCR2 expression relative to wild-type insects. Critically, viral accumulation in these heterozygotes was not significantly different from wild-type controls across all four tested ISVs (Supplementary Figure S8). This genetically independent result is consistent with the knockdown data and further supports DCR2 dispensability for antiviral immunity in *L. striatellus*, even under conditions of reduced DCR2 activity.

Regarding the identification of the nuclease responsible for DCR2-independent siRNA biogenesis, we fully agree that identifying this alternative nuclease represents a compelling priority for future work and we have expanded the Discussion to address this. Moreover, we have also moderated the language in the title and throughout the manuscript to use “several hemipteran insects” instead of “hemipteran insects”, ensuring that our conclusions accurately reflect the species scope of the current evidence.

**Reviewer #3:**

- The conclusion that antiviral RNAi is independent of DCL2 is based on the observation that viral small RNAs are not reduced upon DCL2 knockdown. This conclusion is fully based on a knockdown approach (using RNAi to knockdown RNAi genes), which inevitably is incomplete: Even if RNA levels are strongly reduced (as shown by the authors), residual levels of DCL2 protein may be sufficient to process viral dsRNA. Another explanation for the lack of a phenotype may be based on the use of persistent virus infections. In such a setting, viral siRNAs are already preexisting and loaded in Argonaute 2 before knockdown and it is well-established that Argonaute-loaded small RNAs can have long half-lives. It is therefore possible that a knockdown of Dicer may therefore not significantly affect siRNA profiles.

**Response:** We thank the reviewer for raising these two distinct and important issues. We have addressed them separately:

Regarding residual DCR2 protein activity. To address this concern without relying on knockdown completeness, we performed an RNAi-of-RNAi assay that measures the functional output of the RNAi pathway (dsRNA cleavage efficiency). Pre-knockdown of *Ls_DCR1* or *Ls_DCR2* had no significant effect on the cleavage of subsequently injected *ds-Ls_Tubulin*, whereas pre-knockdown of *Ls_AGO2* caused a pronounced reduction in cleavage efficiency. This functional result is independent of the question of how much residual DCR2 protein remains, because it shows that even when DCR2 is present at its normal concentration (in control insects), it does not drive the RNAi response. If DCR2 were the key dsRNA-processing enzyme, its knockdown would have impaired reporter cleavage regardless of whether the reduction was partial or complete (Supplementary Figure S7). Please see lines 303-314 and 446-458.

Regarding preexisting siRNAs from persistent infections. To overcome it, we utilized an acute infection model by injecting *L. striatellus* with crude extracts of Rice stripe virus (RSV). Because RSV is acquired through injection rather than natural persistent infection, the insects do not carry preloaded RSV-derived siRNAs at the time of dsRNA injection. Knockdown of *Ls_DCR2* in these acutely infected insects also failed to increase RSV accumulation, whereas *Ls_AGO2* knockdown did (Supplementary Figure S4C). This result demonstrates that the DCR2-independent phenotype is not an artifact of preexisting persistent infection.

- To more comprehensively analyze the DCL2 knockdown data, please show siRNA size profiles upon DCL2/AGO2/R2D2 knockdown in Figure 4 (akin to Figure 3A-b). Also, it would be useful to use the data to identify sources of endo-siRNAs and thus validate that DCL2 knockdown functionally affects siRNA levels.

**Response:** We thank the reviewer for this constructive suggestion. To further address potential functional redundant of DCL2/AGO2/R2D2, we performed a triple knockdown experiment simultaneously targeting *Ls_DCR2*, *Ls_AGO2*, and *Ls_R2D2*. The results showed that significant increases in viral abundance and vsiRNA reads were observed in the triple knockdown group, driven primarily by AGO2 depletion. These data further confirm that DCR2 is not essential component of the antiviral siRNA response in *L. striatellus* (Supplementary Figure S6B-C, lines 290-302).

- I am not aware of any other experimental systems in which viral siRNAs are produced in a Dicer-independent manner. The authors suggest that there may be another, yet unidentified, DCL2 like enzyme that produces siRNAs. In that case, the lack of a phenotype for DCL2 knockdown may be a simple, unfortunate consequence of an incomplete reference genome/genome annotation (and the finding would be less remarkable). The authors may want to comment on the completeness of the reference genome.

**Response:** We thank the reviewer for raising this important point. Our study relies on the chromosome-level reference genome of *L. striatellus* published by Wang et al. (2021; GenBank: GCA_017141395.1; *Genome Biology and Evolution*, doi: 10.1111/1755-0998.13242). This assembly is a high-quality completeness genome which was confirmed using BUSCO assessments against multiple lineage-specific databases (Eukaryota, Arthropoda, Hemiptera, and Insecta). The genome annotation was used to conduct systematic homology-based searches for all known RNase III domain-containing proteins. No additional Dicer-like genes beyond DCR1 and DCR2 were identified. We therefore consider it unlikely that the observed DCR2-independent siRNA production reflects a missing or misannotated DCR2-like gene in this genome.

- Has potential redundancy between DCL1 and DCL2 been assessed via a double knockdown? This would still be an important experiment to perform.

**Response:** We thank the reviewer for this important suggestion. We have now performed this experiment. Third-instar nymphs of *L. striatellus* were co-injected with dsRNAs targeting both *Ls_DCR1* and *Ls_DCR2* simultaneously. The knockdown efficiency of both genes was confirmed by RT-qPCR. Importantly, even under these double knockdown conditions, neither viral RNA accumulation nor vsiRNA profiles differed significantly from those in ds-*GFP* controls. These results rule out functional redundancy between DCR1 and DCR2, and are consistent with the conclusion that antiviral siRNA production in *L. striatellus* is independent of DCR2 proteins (Supplementary Figure S6A, C; lines 290-302).

- I applaud the authors efforts to produce a DCL2 knockout using CRISPR. Unfortunately, the animals did not survive into adulthood to assess antiviral activity at this stage. Yet, some early stage homozygous nymphs were recovered. Are the persistent infections already detectable at this stage? If so, the authors should analyze viral RNA levels and viral small RNA profiles to solidify their conclusion.

**Response:** We sincerely thank the reviewer for this constructive suggestion and for acknowledging our efforts in generating a DCR2 knockout line. We agree that analyzing viral RNA levels and vsiRNA profiles in homozygous DCR2 mutant embryos would be of great value. However, this analysis is currently not technically feasible in our experimental system. Genotyping of homozygous mutant embryos requires DNA extraction from individual, unhatched eggs. To simultaneously obtain RNA from the same individual for gene expression analysis, DNA and RNA would need to be co-extracted from a single egg. The total amount of RNA recoverable from a single egg at this developmental stage is insufficient to meet the quantity and quality requirements for reliable RT-qPCR or small RNA library construction.

As an alternative, we analyzed viral accumulation in DCR2-heterozygous mutants, which carry one functional and one disrupted Ls_DCR2 allele and can survive to adulthood. Both heterozygous mutant lines (MT1 and MT2) showed significantly reduced DCR2 mRNA levels compared to wild-type insects, yet viral accumulation across four tested ISVs was not significantly different from that of wild-type controls (Supplementary Figure S8). These results are consistent with the knockdown data and support the conclusion that even partial reduction of DCR2 activity does not affect antiviral immunity. We have clearly described these technical limitations and the alternative approach in the revised manuscript (lines 338-341).

- The authors report a role for DCL2/RNAi in development based on the observation that no homozygote adults were found. The mechanism by which RNAi affects development has not been studied. I would encourage the authors to at least perform small RNA profiles in early stage animals to study whether endogenous siRNAs are lost in these animals to provide a first glimpse on a mechanism by which RNAi controls development.

**Response:** We thank the reviewer for this insightful suggestion. We agree that understanding the mechanistic basis by which DCR2 controls development in hemipteran insects is an important and compelling question. As described above, the technical challenge of co-extracting sufficient quantities of DNA and RNA from single homozygous mutant eggs currently precludes sRNA profiling from confirmed homozygous DCR2 knockout individuals. The embryonic lethality of homozygous mutants and the extended developmental duration in heterozygotes, collectively point to an essential developmental function of DCR2 in *L. striatellus*. Dissecting the mechanistic basis of this developmental role, including whether it involves endogenous siRNA pathways, represents an important direction for future research.

- Small RNA read counts are very low in Figure 3B compared to figure 3A. Moreover, miRNAs are notably low compared to figure 3A. It seems that something went wrong in the sequencing or data analyses. Please double check and/or repeat the sequence/analyses. Also, reads are plotted as negatives. Am I correct in assuming that this reflects the “sense” and “antisense” strands of the genome? If so, this would not be meaningful as genes and transposable elements can be in both orientations on the genome. I suggest plotting similarly to Figure 3A (i.e., only total counts).

**Response:** We thank the reviewer for this careful observation and for the constructive suggestion. We have carefully re-examined the sequencing data and bioinformatic analyses and confirmed that the read counts in both panels are correct. The apparent difference in read counts between Figure 3A and B may reflect the genuinely distinct abundances of these two small RNA classes in *L. striatellus*. Previous study has reported that piRNAs are present at substantially lower levels than miRNAs in somatic tissues under normal conditions (Nucleic Acids Res, 2011, doi: 10.1093/nar/gkr298). Regarding the negative values in Figure 3B(b), we fully agree with the reviewer that this representation is not meaningful in this context. Following the reviewer’s suggestion, we have replotted Figure 3B(b) to display only total counts, in the same format as Figure 3A. Please see the revised Figure 3.

- Effect of miRNA pathway genes are only analyzed for their effects on total small RNA populations (Figure 3A). I would encourage to also analyze small RNA reads mapping to miRNAs directly. Should miRNAs not be annotated in the genome, it would be worthwhile using their data to annotate miRNAs.

Response: We thank the reviewer for this technically specific and constructive suggestion. We fully agree that mapping sRNA reads directly to annotated miRNA loci, rather than relying solely on size-distribution profiling, provides a more rigorous and interpretable measure of miRNA pathway disruption.

However, we respectfully note that the primary purpose of the sRNA-seq experiment in Figure 3A was not to characterize miRNA populations in depth, but to serve as a functional readout confirming that knockdown of *Ls_AGO1* and *Ls_DCR1* effectively disrupts the miRNA pathway. The observed reduction in total 22-nt reads is consistent with established roles of these factors in miRNA biogenesis and is sufficient to confirm effective pathway disruption for the purposes of our antiviral assays. Crucially, despite this clear reduction in miRNA abundance, knockdown of neither *Ls_AGO1* nor *Ls_DCR1* led to any detectable change in viral RNA accumulation or vsiRNA profiles (Supplemental Figure S5A), which is the central conclusion we draw from this part of the study.

We recognize that a locus-level miRNA analysis and de novo annotation would add value to the field and represent an important direction for future work dedicated specifically to miRNA biology in hemipteran insects. An in-depth characterization of *L. striatellus* miRNA populations, including differential expression upon pathway knockdown, is currently being planned in our laboratory as a dedicated follow-up study.

**Part III – Minor Issues: Editorial and Data Presentation Modifications**

Please use this section for editorial suggestions as well as relatively minor modifications of existing data that would enhance clarity.

**Reviewer #1**

The figure legends should indicate sample sizes, exact n values, and clarify whether each dot represents an independent biological replicate in Figures 2–6. Providing these details will improve transparency and allow readers to better evaluate data variability and robustness.

**Response:** We thank the reviewer for this helpful suggestion. All figure legends for Figures 2-6 have been revised to explicitly state the exact n values, the number of independent biological replicates, and whether each dot in scatter plots represents an independent biological replicate or an individual insect.

The Methods section should be expanded to include essential experimental parameters such as the concentration of dsRNA used, the developmental stages at which injections were performed, and the precise sampling time points after injection.

**Response:** We thank the reviewer for this valuable suggestion. The Materials and Methods section has been comprehensively expanded to include dsRNA concentrations, the specific developmental stages at which injections were performed, and the precise time points at which samples were collected following injection. Please refer to the revised Materials and Methods section.

**Reviewer #2:**

Minor re-analysis:

The phylogenetic analysis should be revisited. In its current state, the protein-by-protein analysis is geared towards replicating the evolutionary distance of the organisms included. The analysis would become more informative if the clustering would be less supervised a priori, for instance by making a phylogenetic tree for the Argonaute proteins and separate trees for PIWI proteins and Dicer proteins, similar to the study ‘Comparative genomics of small RNA regulatory pathway components in vector mosquitoes’ (doi: 10.1186/1471-2164-9-425). For completeness, the PIWI protein Aubergine from Drosophila should be included in the analysis.

**Response:** We thank the reviewer for this constructive suggestion. The phylogenetic analysis has been revised to produce separate, unsupervised trees for the Argonaute (AGO clade), PIWI, and Dicer protein families. *Drosophila* Aubergine has been added to the PIWI tree. Please see the revised Figure 1.

**Reviewer #3:**

- Number of observations and replicates should be reported in the legends for all figures. Error bars need to be defined (SEM or STD).

**Response:** We thank the reviewer for this important suggestion. All figure legends have been revised to clearly state the number of biological replicates and individual observations (n values), and to specify that error bars represent the standard error of the mean (SEM). These revisions have been applied consistently across all figures in the manuscript.

- Fig. 2: Pictures of the molting defects require higher resolution figures and quantification. Also, for the reader without experience with this animal, it may be useful to briefly describe how this is visible from the figure.

**Response:** We thank the reviewer for this helpful suggestion. Higher-resolution images of the molting defects have been included in the revised Figure 2. In addition, a brief description of the characteristic visible features of molting defects in *L. striatellus* has been added to the main text (lines 183-184).

- Fig. 2: extremely minor differences which large overlaps between the range of data are noted as statistically significant. Please verify that this is correct and that the correct statistical test has been used.

**Response:** We thank the reviewer for this concern. We have carefully re-examined all statistical analyses in Figure 2. Survival curves are now analyzed using the Log-rank (Mantel-Cox) test, which is the appropriate method for comparing survival distributions (lines 634-635, 912-913).

- For consistency with piRNA and siRNA data, please also show the development duration of nymphs for Ago1 and DCL1.

**Response:** We thank the reviewer for this suggestion. Unfortunately, due to the severe developmental defects and early mortality caused by knockdown of *Ls_AGO1* and *Ls_DCR1*, it was not possible to reliably quantify developmental duration for these conditions. When *Ls_AGO1* was silenced at the first-instar stage, nearly all nymphs died at the second instar. Following *Ls_DCR1* knockdown, approximately 57% of nymphs survived to the third instar and ~30% to the fourth instar, but none reached the fifth instar. As a result, developmental duration statistics for these treatment groups cannot be reliably calculated or compared.

- Fig. 2, the legend reports that ANOVA and T tests were used in this figure. However, this is not appropriate for survival curves. Please use an appropriate test to analyze the survivals.

**Response:** We thank the reviewer for this correction. Survival curves in Figure 2 are now analyzed using the Log-rank (Mantel-Cox) test, and the figure legend has been updated accordingly. Please see the revised Figure 2 and lines 634-635, 912-913.

- Figure 3. Define the red and black bars in the legends.

**Response:** We thank the reviewer for this comment. The figure legend for Figure 3 has been updated to specify that black bars represent the sense strand and red bars represent the antisense strand (revised legend for Figure 3, lines 923-924).

- Fig. 3C-G: How similar are the EVE sequences? Can the small RNAs be unambiguously mapped to the individual elements?

**Response:** We performed pairwise sequence alignments among the five EVE sequences. The results confirmed that there is no sequence similarity among them. Therefore, small RNAs can be unambiguously and uniquely assigned to each individual EVE element.

- T test is not appropriate to analyze the data in Figure S4 as it assumes a normal distribution, and the data do not seem to be normally distributed. I would also advice the authors to verify normal distributions for other figures in which T tests and ANOVA’s were used.

**Response:** We thank the reviewer for this observation. We have re-evaluated the data distribution in Figure S4 and re-analyzed the data using ANOVA test.

- Please, consistently show individual data points in all figures showing bar graphs. Also, ensure consistency in plotting (e.g., in Fig. 5C dot plots are shown whereas in Fig. 5A/B bargraphs were used for the same data types).

**Response:** We thank the reviewer for this comment. We have ensured consistency in data presentation across all figures. For sRNA sequencing data (Figures 6A and 6B), three biological replicates were used, with each replicate comprising a pool of 15-20 insects. For Figure 6C (whitefly data), individual insects were used due to the inherent variability in virus prevalence, and each data point represents a single confirmed viruliferous insect. The distinction between pooled and individual samples has been clearly stated in all revised figure legends.

- Line 245-247: The conclusion that Ago3 is not involved in the piRNA pathway cannot be simply drawn from the number of piRNAs. If there are only few ping-pong derived piRNAs in this species, no strong phenotype is expected for Ago3 knockdown. Indeed, Ago3 knockdown shows a phenotype in hatch rates in Fig S3, suggesting an essential role of Ago3. Rephrase the conclusion. Likewise, line 410 is not supported by the data.

**Response:** We thank the reviewer for this important point. We agree that the number of piRNAs alone is not sufficient to conclude that Ago3 is not involved in the piRNA pathway. As shown in Figure S3, Ago3 knockdown affects hatch rates, indicating that Ago3 plays an essential role. We have revised the manuscript to clarify this point and have rephrased the statements in lines 251-256 and 416-420 to more accurately reflect the data.

Suggestions for data and text presentation:

- Fig 2B, d: define stage of the animals in the legend.

**Response:** We thank the reviewer for the suggestion. The developmental stage of the insects has been clearly defined in the revised figure legend for Figures 2B-d and 2C-d (lines 897-899, 907-909).

- Define the green dashed lines in Figure 4

**Response:** We thank the reviewer for pointing this out. The green dashed lines in Figure 4 have been defined in the revised figure legend (lines 934-935). They indicate the position of the 22-nt vsiRNA peak, which facilitates visual comparison across treatment groups.

- Fig. 1. The Drosophila PIWI gene Aubergine is lacking from the phylogenetic tree. I suggest adding them. Also, Apis Ago1 and Ago2 seem to be lacking from the trees. While not essential, it may be useful to add for consistency.

**Response:** We thank the reviewer for this suggestion. *Drosophila* Aubergine has been added to the revised phylogenetic tree. Regarding *Apis mellifera* AGO1 and AGO2: these sequences were not found in the NCBI protein database and therefore could not be included.

- Fig. 1. Define the scale bar

**Response:** The scale bar has been added and defined in the revised Figure 1.

- Fig. 5C: the figure is a bit unclear to me. What are the 6 dots. I would advise the authors to also report the number of offspring for the different genotypes to make the figure more informative.

**Response:** We thank the reviewer for this comment. The dot plot in Figure 5C has been removed. The number of offspring for each genotype has been added in the main text (lines 329-333).

- Fig. 5D and 5E: The figure would benefit from a description for what is shown in the figure for people not working with these insects. Also, a wildtype control should be provided as comparison.

**Response:** We thank the reviewer for this suggestion. Detailed descriptions of the experimental procedure and what is shown in revised Figures 5D (5E was merged to 5D) have been added to the figure legends. Briefly, heterozygous female and male mutants were crossed and allowed to oviposit on fresh rice seedlings for one day. After removing the adults, unhatched eggs remaining on seedlings after 10 days were dissected and genotyped by Sanger sequencing. As a control, 5-day-old wild-type eggs were included for comparison. Please see revised lines 946-951.

- Fig. 5D and 5E: the electropherogram of the homozygotes adds little information and could be deleted.

**Response:** We thank the reviewer for this suggestion. The electropherograms of the homozygotes in Figures 5D and 5E have been removed.

- Table S2: as I understand the table, it seems that the numbers report the number of animals with the indicated genotypes and not the “failure to hatch” as the title suggests. Please correct.

**Response:** We thank the reviewer for this correction. The data in Table S2 report the genotypes of individual unhatched eggs dissected from rice seedlings on which G2 heterozygous mutant pairs had oviposited. The title has been revised to accurately reflect that the table describes genotype composition among unhatched eggs. Please see the revised Table S2.

- Fig. S7: please report how many animals have been analyzed. Also, in my opinion, the symbols in the insect and under the graph add little information.

**Response:** We thank the reviewer for this suggestion. The exact number of insects analyzed (n=45) has been added to the figure legend of the revised Figure S9 (previous S7). Please refer to line 1032. Following the reviewer’s advice, the schematic insect symbols and icons below the graph have been removed to simplify the figure.

- Line 51: “it is triggered by dsRNA” is not entirely correct for piRNA pathway. Please correct.

**Response:** We thank the reviewer for this correction. This sentence has been removed from the revised manuscript.

- Line 63: “siRNAs are dsRNAs around 20-25 nt in length, targeting either exogenous or endogenous dsRNA”. I suggest rephrasing as siRNAs are not targeting dsRNA, rather they are derived from dsRNA.

**Response:** We thank the reviewer for the clarification. The sentence has been corrected to: "siRNAs are approximately 20-25 nt in length, derived from double-stranded RNA, and mediate gene silencing by guiding the degradation of complementary single-stranded transcripts” (lines 78-80).

- Line 192: please check the grammar.

**Response:** Done. The grammar has been corrected at lines 210-216 of the revised manuscript.

- Line 267-268: a brief introduction of these viruses may be useful.

**Response:** We thank the reviewer for this helpful suggestion. Brief introductory information for the ISVs used in this study has been provided in the Materials and Methods section (lines 563-576), which is cross-referenced in the Results text.

- Line 333 onwards: from the text it is unclear which virus was found in which species. Also, it is unclear how these viruses were identified in the first place. Please provide this information.

**Response:** We thank the reviewer for this suggestion. The relevant text has been revised to clearly specify which viruses were identified in which insect species and how they were identified. For *S. furcifera*, viruses were identified by screening NCBI databases for previously reported ISVs and confirming their presence in our laboratory population by RT-PCR. For *N. lugens*, four novel bunya-like viruses were identified through virome characterization conducted in our laboratory. Please see lines 353-354 and 373.
