# Supplementary material for: Functional dissection of conserved RNAi pathways in antiviral immunity of several hemipteran insects
Source: PLoS Pathog. 2026 Oct 30;22(10):e1014753. doi: 10.1371/journal.ppat.1014753 (PMC13734579; doi:10.1371/journal.ppat.1014753)
Supplement: Supplementary file 6 [file ppat.1014753.s018.docx]

**Response to Reviewers-Revision2**

**Manuscript (PPATHOGENS-D-25-02414R2)**

***“Functional dissection of conserved RNAi pathways*** ***in antiviral immunity of several hemipteran insects”***

**Reviewers' Comments:**
Reviewer's Responses to Questions

**Part I - Summary**
Please use this section to discuss strengths/weaknesses of study, novelty/significance, general execution and scholarship.

Reviewer #1: I appreciate the substantial effort made in revising the manuscript. The additional experiments have strengthened the study and address several of my initial concerns.
However, I remain concerned that the central claim is still overstated. The manuscript repeatedly refers to DCR2-independent vsiRNA biogenesis. Based on the current data, this conclusion is stronger than what can be directly supported. The results demonstrate that DCR2 is not required or not limiting in the tested system, but they do not definitively exclude a contribution of DCR2 to vsiRNA production at the mechanistic level. In particular, residual DCR2 activity, partial redundancy, or the presence of additional nucleases cannot be ruled out. The current experiments do not directly identify the enzyme responsible for vsiRNA generation.
I therefore recommend that the authors moderate the wording throughout the manuscript. More cautious phrasing such as DCR2 is dispensable under the tested conditions, or vsiRNA production is not detectably affected by DCR2 depletion, would better reflect the data.

**Response:** We sincerely thank you for the positive assessment of our revision and for this thoughtful and constructive recommendation. We fully agree that our previous interpretation extended beyond what can be directly supported by the current data. Although our additional experiments consistently showed that DCR2 depletion did not produce detectable antiviral phenotypes under the tested conditions, they do not definitively exclude the possibility that residual DCR2 activity, partial functional redundancy, or other unidentified mechanisms may contribute to vsiRNA biogenesis. Likewise, the current study does not directly identify the nuclease responsible for vsiRNA generation.

Following your recommendation, we have comprehensively revised the manuscript to ensure that our conclusions are fully aligned with the available evidence. Rather than making mechanistic claims regarding DCR2 independence, the revised manuscript now focused on the observed experimental phenotypes while explicitly acknowledges the limitations of the current study. Specifically, we have:

(1) Revised the title to remove the central claim of “Dicer2-independent antiviral defense”, and now the title is “**Functional dissection of conserved RNAi pathways in antiviral immunity of several hemipteran insects**” (Lines 1-3).

(2) Systematically revised the Abstract, Author Summary, Introduction, Results, and Discussion to replace definitive mechanistic statements with more cautious, observation-based descriptions, such as “DCR2 depletion did not produce detectable antiviral phenotypes under the tested conditions,” while explicitly stating that residual DCR2 activity or alternative mechanisms cannot be excluded (Abstract: Lines 27-35, 38-49; Author Summary: 59-69; Introduction: 142-152; Results: 285-289, 331-337, 369-370, 402-407; Discussion: 413-423, 473-480, 483-486, 491-502, 509-512, 518-534).

We appreciate your guidance, which has helped us substantially improve the scientific rigor and balance of the manuscript. We believe the revised version now more accurately reflects the scope and limitations of our findings.

Reviewer #2: In the revised manuscript “Functional dissection of conserved RNAi pathways reveals Dicer2-independent antiviral defense against RNA viruses in several hemipteran insects” by Zhang et al. the authors have performed additional experiments to support their central claim that RNAi operates independently of Dicer2 in four hemipteran species. The two critical additions are an RNAi-of-RNAi assay and analysis of virus replication in heterozygous mutants. However, both of these assays do not address the major concern of residual protein activity. I disagree with the reviewers that the tubulin RNAi reporter assay firmly proofs Dicer2 independent RNAi ‘regardless of residual protein levels’ (line 313). Incomplete DCR2 depletion may still generate sufficient levels of tub siRNAs which may explain silencing of tubulin RNA as observed by qPCR in supplemental figure 7B. The conceptual difference compared to assays reported in figure 4 is that the target in this assay is not a replicating viral RNA but a cellular transcript. Yet, this change in experimental setup does not address the concern of residual protein activity. Likewise, heterozygous DCR2 mutants intrinsically have remaining DCR2 protein expression. Therefore, my initial concern remains, that the high burden of proof for DCR2-independent RNAi has not been met by the revised manuscript.

**Response**: We sincerely thank you for this rigorous and insightful evaluation. We fully agree that neither the RNAi-of-RNAi assay nor the analysis of heterozygous DCR2 mutants can definitively exclude the possibility that residual DCR2 protein remains sufficient to support baseline vsiRNA biogenesis. We also agree that these experiments do not directly identify the nuclease responsible for vsiRNA generation. Therefore, we recognize that our previous interpretation exceeded the level of evidence provided by the current study.

After carefully considering the reviewers’ comments together with the Editor's recommendation, we chose not to further strengthen our original interpretation using additional indirect evidence. Instead, we comprehensively revised the manuscript to ensure that all conclusions are now strictly limited to the experimental observations supported by the available data.

Specifically, we have revised the title and systematically rewritten the Abstract, Author Summary, Introduction, Results, and Discussion. The revised manuscript no longer concludes that antiviral RNAi in hemipteran insects is DCR2-independent or that DCR2 is dispensable for vsiRNA biogenesis. Instead, it consistently states that DCR2 depletion did not produce detectable antiviral phenotypes under the tested experimental conditions, while explicitly acknowledging throughout the manuscript that residual DCR2 activity, partial functional redundancy, or alternative mechanisms cannot be excluded, and that the potential nuclease responsible for vsiRNA biogenesis remains to be identified (Title: Lines 1-3; Abstract: Lines 27-35, 38-49; Author Summary: 59-69; Introduction: 142-152; Results: 285-289, 331-337, 369-370, 402-407; Discussion: 413-423, 473-480, 483-486, 491-502, 509-512, 518-534).

We sincerely appreciate your careful evaluation. We believe these extensive revisions substantially improve the accuracy and scientific balance of the manuscript, and that the revised conclusions now appropriately reflect the scope and limitations of the available evidence.

Reviewer #3: This is a revision of a manuscript addressing the role of RNAi in antiviral defense in hemipteran insects. My main concern on the first version of the manuscript (and that of the two other reviewers) was that the conclusion of Dicer-2 independent antiviral defense was not supported by the data. The authors have performed additional experiments to try to provide stronger support.

**Response**: We sincerely thank you for carefully re-evaluating our revised manuscript and for recognizing our efforts to strengthen the study through additional experiments. We fully agree that our previous conclusion regarding DCR2-independent antiviral defense was stronger than could be directly supported by the available evidence.

After carefully considering the comments from all reviewers and the Editor, we have comprehensively revised the manuscript to ensure that the conclusions are fully aligned with the current experimental evidence. Rather than making definitive mechanistic claims regarding DCR2 independence, the revised manuscript now consistently describes the observed experimental phenotypes and explicitly acknowledges the limitations of the present study. Specifically, we have revised the title and systematically updated the Abstract, Author Summary, Introduction, Results, and Discussion to remove overinterpretation and to emphasize that DCR2 depletion did not produce detectable antiviral phenotypes under the tested conditions, while recognizing that residual DCR2 activity, alternative nucleases, or other mechanisms cannot be excluded (Title: Lines 1-3; Abstract: Lines 27-35, 38-49; Author Summary: 59-69; Introduction: 142-152; Results: 285-289, 331-337, 369-370, 402-407; Discussion: 413-423, 473-480, 483-486, 491-502, 509-512, 518-534).

We appreciate the reviewer's constructive comments, which have helped us improve the scientific accuracy and balance of the manuscript. Our point-by-point responses to the remaining comments are provided below.

**Part II – Major Issues: Key Experiments Required for Acceptance**

Reviewer #1: (No Response)

Reviewer #2: A genetically clean assay that genuinely addresses the concern of residual protein activity is needed to convincingly show DCR2-independent RNAi.

**Response**: We fully agree with the reviewer that a genetically clean assay capable of completely eliminating residual DCR2 activity would be required to definitively demonstrate DCR2-independent antiviral RNAi. As discussed in our response above, the current study does not provide such evidence. Accordingly, rather than continuing to support this mechanistic claim with additional indirect experiments, we have comprehensively revised the manuscript to remove all conclusions regarding DCR2-independent antiviral RNAi. The revised manuscript now consistently states that DCR2 depletion did not produce detectable antiviral phenotypes under the tested experimental conditions, while explicitly acknowledging that residual DCR2 activity or alternative mechanisms cannot be excluded (see our response to Part I, Reviewer #2, and the corresponding revisions in the Abstract, Results, and Discussion). We believe this revision more accurately reflects the scope and limitations of the current study.

Reviewer #3: Unfortunately, I remain unconvinced of the main conclusion of Dicer indendence:
1) The main argument remains centered on the use of an RNAi approach to knock down RNAi genes. The extra experiment provided in Figure S7 actually strengthens my concern: prior knockdown of Dicer 1 and Dicer 2 do not affect silencing of tubulin by dsRNA. The authors are not correct in their rebuttal with their statement in the text “This functional result is independent of the question of how much residual DCR2 protein remains”. RNAi of Dicer2 is critically dependent on the amount of Dicer2 protein remaining at the time of the second (tubulin specific) dsRNA. Although the authors refer to work that RNAi-of-RNAi worked in other insects, it is likely that there are gene specific differences in RNAi-of-RNAi efficiency, which may be related to differences in protein stability and turnover. The experiment of Figure S7 indicates that it had not been possible to sufficiently knock down Dicer2 to affect RNAi, and it is therefore not surprising that no viral phenotypes have been obtained.

**Response**: We sincerely thank the reviewer for this insightful comment. We fully agree that the RNAi-of-RNAi assay cannot exclude the possibility that residual DCR2 protein remained sufficient to support silencing of the subsequently introduced Tubulin dsRNA. Therefore, we agree that this experiment cannot provide definitive mechanistic evidence for DCR2-independent RNAi, and our previous interpretation overstated what could be concluded from these data.

Accordingly, we have revised both the manuscript and our interpretation of this experiment. Specifically, we have removed the previous statement suggesting that the RNAi-of-RNAi assay was independent of residual DCR2 protein levels. In the revised manuscript, this experiment (now presented as **Figure S9**) is described only as a complementary functional observation and is no longer used to support a mechanistic conclusion regarding DCR2 function. Instead, we explicitly acknowledge that residual DCR2 activity may have been sufficient to sustain RNAi activity and that this possibility cannot be excluded.

As described in our response to Part I (Reviewer #3), we have also comprehensively revised the manuscript to remove all mechanistic claims regarding DCR2-independent antiviral RNAi. The revised manuscript now consistently limits its conclusions to the observed knockdown phenotypes and explicitly discusses the limitations of the current evidence.

2) While the combined knockdown does address potential redundancy between Dicer1 and Dicer2, but the experiment suffers from the limitation of using RNAi to knock down RNAi genes.

**Response**: We thank you for this important comment. We agree that the combined DCR1/DCR2 knockdown has an inherent methodological limitation because it relies on RNAi to deplete components of the RNAi pathway itself. Therefore, this approach cannot exclude residual DCR2 activity or provide definitive mechanistic evidence regarding DCR2 function.

The experiment was intended only to test whether simultaneous depletion of DCR1 and DCR2 would reveal detectable functional redundancy under the conditions examined. As described in our response to Part I (Reviewer #3), we have revised the manuscript so that this result is no longer interpreted as evidence for DCR2-independent antiviral RNAi.

3) The experiment with heterozygous Dicer mutants (no antiviral phenotype despite reduced Dicer2 expression) is interesting, but not conclusive: Both Dicer and Argonaute are multi-turnover enzymes and reduced siRNA levels in heterozygous Dicer2 mutants may already suffice to mediate effective antiviral RNAi. Hence, there is unlikely to be a linear relation between Dicer expression and RNAi efficiency.

The most parsimonous conclusion of these experiments is that RNAi of RNAi simply does not work for Dicer2 in this species and that results from such experiments cannot be used to conclude that antiviral defense is Dicer2 independent. An alternative conclusion would be that there is another, yet unknown, nuclease generating the viral siRNAs that are loaded in Argonaute 2. However, in their rebuttal the authors indicate that this is not likely as the reference genome is high-quality.

Overall, I appreciate the authors efforts to convince the reviewers of their conclusions, but I remain unconvinced of their main conclusion. To move this manuscript forward, I would propose that the authors tone down their conclusion of Dicer2 indepence throughout the manuscript.

**Response**: We sincerely thank the reviewer for this thoughtful summary and constructive recommendation. We agree that, although our additional experiments provide useful observations, they do not constitute definitive mechanistic evidence for DCR2-independent antiviral RNAi. We therefore fully accept the reviewer's recommendation that our previous conclusion exceeded the level of evidence provided by the current study.

Accordingly, as described in our response to Part I (Reviewer #3), we have comprehensively revised the manuscript to remove all claims of DCR2-independent antiviral RNAi. The revised manuscript now consistently limits its conclusions to the observed experimental phenotypes under the tested conditions and explicitly acknowledges that residual DCR2 activity, an unidentified nuclease, or other alternative mechanisms cannot be excluded. We believe these revisions more accurately reflect both the strengths and the limitations of the current study.

Other comments:
- My original suggestion to use small RNA data to identify sources of endo-siRNAs and validate that DCR2 knockdown functionally affects siRNA levels has not been addressed, nor rebutted. This should still be done. In this regard, the authors should also provide the total size distribution of the Dicer2/Ago2 knockdown, akin to those provided for miRNA and piRNA genes (Fig. 3A(b) and 3B(b)).

**Response**: We thank the reviewer for this helpful suggestion. As suggested, we analyzed the size distributions of total miRNAs following *DCR2* knockdown and total piRNAs following combined *DCR2*/*AGO2*/*R2D2* knockdown. DCR2 knockdown did not detectably alter the overall miRNA size profile, while the triple knockdown similarly had no significant effect on the overall piRNA size profile. These data are now presented in the **newly added Figure S8** and described in the revised Results (Lines 317-321). We have limited our interpretation to these observed global small-RNA profiles and do not consider this analysis evidence for DCR2-independent siRNA biogenesis.

- The author’s response to my earlier comment that “Small RNA read counts are very low in Figure 3B compared to figure 3A.” is not entirely convincing. In Panel 3A(b) the number of 22-nt small RNAs in dsGFP control is approximately 120,000 RPM whereas this was only ~1500 RPM in panel 3B(b). This discrepancy requires an explanation. Were reads normalized to total library size or to total genome mapped reads? Are there many non-mapping reads in the libraries of panel 3B(b)? It seems that there is a large proportion of reads not accounted for.

**Response**: We sincerely thank the reviewer for pointing out this issue and apologize for the confusion caused by our previous presentation. Following the reviewer’s suggestion, we have re-analyzed the small RNA datasets and revised Figure 3 accordingly. In the updated Figure 3A(b), only annotated and predicted miRNAs were included in the analysis, whereas Figure 3B(b) presents the piRNA population. Thus, the two panels now represent distinct classes of small RNAs rather than total 22-nt and 27-28-nt reads, and their RPM values are not intended for direct quantitative comparison. We believe that this revision provides a clearer and more accurate representation of the miRNA and piRNA datasets and eliminates the ambiguity present in the previous version.

- Figure 1A: R2D2 and Dicer enzymes have a vastly different domain structure and the sequences are unlikely to align (or only by introducing may/large gaps in the sequence). The trees should be made separately for the R2D2 and Dicer genes.

**Response**: We sincerely thank the reviewer for this helpful suggestion. We fully agree that Dicer and R2D2 proteins differ substantially in their domain architectures and should not be analyzed in a single phylogenetic tree. Accordingly, we have reconstructed the phylogenetic analyses by separating the Dicer and R2D2 proteins into independent trees. The revised Figure 1 now presents separate evolutionary analyses for the Dicer, R2D2, and Argonaute protein families.

- Figure 4: I agree with Reviewer 1 that the number of viral siRNAs in Ago2 knockdown should be normalized to the viral RNA levels. The authors indicate that RPM is a standard approach to normalize the data (which is correct), but the authors can divide these numbers by the relative viral RNA levels from the qPCR assay shown in the right panel of figure 4B. It is not sufficient to merely suggest normalization to viral levels in the discussion.

**Response**: We thank the reviewer for this valuable suggestion. As recommended, we normalized the virus-derived small RNA read counts to the corresponding viral copy numbers for each treatment group to account for differences in viral RNA abundance. These analyses are now presented in the newly added Supplemental Figure S4 and described in the revised Results (Lines 276-281). After normalization, the virus-derived small RNA reads/copies ratio was significantly reduced in the AGO2 knockdown group compared with the control, whereas no significant differences were observed following DCR2 or R2D2 knockdown. We believe this normalization provides a more appropriate comparison of virus-derived small RNA production among the different RNAi pathway knockdown treatments.

- The authors refer to custom Python script. For reproducibility, these script should be deposited, e.g. in GitHub.

**Response**: We thank the reviewer for this helpful suggestion. Following the reviewer's suggestion, we have deposited the custom Python scripts used for small-RNA classification and annotation in a publicly accessible GitHub repository: **https://github.com/Zhuangxinye/vsirna-pipeline**. The repository link has also been added to the Materials and Methods section of the revised manuscript (Lines 645–647).

**Part III – Minor Issues: Editorial and Data Presentation Modifications**

Reviewer #1: (No Response)

Reviewer #2: My previous minor issues have been addressed appropriately.

**Response**: We sincerely thank the reviewer for the positive assessment and are pleased that the previous minor comments have been satisfactorily addressed.

Reviewer #3: - Page 32: typo: Eepression

**Response**: We thank the reviewer for identifying this typographical error. “Eepression” has been corrected to “Expression” (Line 1080). We have also carefully proofread the revised manuscript to correct any remaining typographical errors.

- Fig S9: y axis: I would suggest replacing ‘infective ratio’ with ‘infection rates’ (as also used in the legend)

**Response**: We thank the reviewer for this helpful suggestion. The y-axis label in the revised Figure S11 (previous Figure S9) has been changed from “infective ratio” to “infection rate” to ensure consistency with the terminology used in the figure legend.
