# Supplementary material for: Functional dissection of conserved RNAi pathways in antiviral immunity of several hemipteran insects
Source: PLoS Pathog. 2026 Oct 30;22(10):e1014753. doi: 10.1371/journal.ppat.1014753 (PMC13734579; doi:10.1371/journal.ppat.1014753)
Supplement: Supplementary file 7 [file ppat.1014753.s019.docx]

**Response to Editor and Reviewers (R3)**

**Manuscript (PPATHOGENS-D-25-02414.R3)**

***“Functional dissection of conserved RNAi pathways*** ***in antiviral immunity of several hemipteran insects”***

**Additional Editor Comments (if provided):**
Dear authors, we appreciate all the revisions you have performed to improve the manuscript. Overall the reviewers are positive, however they have identified a few minor comments to be addressed.

**Response:** We sincerely thank the Editor for the positive assessment of our revised manuscript. We have carefully addressed all the remaining comments and journal requirements raised by the Editor and the reviewers and have revised the manuscript accordingly. During our final data verification, we also corrected minor labeling and data-presentation inconsistencies in Figures 2 and 3 and Supplemental Figure S2 to ensure consistency with the underlying data, and provided an S1 Data file containing the underlying numerical data supporting the figures. Detailed responses are provided below.

**Journal Requirements:**
1) We noticed that you used "unpublished data" in the manuscript. We do not allow these references, as the PLOS data access policy requires that all data be either published with the manuscript or made available in a publicly accessible database. Please amend the supplementary material to include the referenced data or remove the references.

If the reviewer comments include a recommendation to cite specific previously published works, please review and evaluate these publications to determine whether they are relevant and should be cited. There is no requirement to cite these works unless the editor has indicated otherwise.

**Response**: We thank the Editor for pointing this out. We have removed “unpublished data” from the revised manuscript (Line 365). The corresponding GenBank accession numbers for the four insect-specific viruses (ISVs) identified in our laboratory population of *Nilaparvata lugens* are provided in the manuscript.

**Reviewers' Comments:**
Reviewer's Responses to Questions

**Part I - Summary**
Please use this section to discuss strengths/weaknesses of study, novelty/significance, general execution and scholarship.

Reviewer #2: I appreciate that the authors have decided to tone down the claim about DCR2-independent processing of siRNAs throughout the manuscript. In line with these changes, I encourage the authors to change the statement ‘implying that siRNA-mediated antiviral pathway in hemipteran insects might be distinct to that of Drosophila and mosquitoes.’ (line 270), which is still an overstatement, despite the careful phrasing. In my opinion, there is no need to make a direct comparison to other Dipteran insects, and I recommend keeping this statement descriptive focusing on the data obtained in this study.

**Response:** We thank the reviewer for this helpful suggestion. We have removed the comparative statement and revised the sentence to focus solely on our experimental observations. Please refer to Lines 274-276 of the revised manuscript (tracked version).

Reviewer #3: This is a second revision of a manuscript analyzing the role of RNAi genes in antiviral defense in hemipteran insects. The authors have adjusted their initial conclusion of Dicer2 independent viral siRNA production, as requested by all reviewers. Current conclusions are now supported by the presented data.

**Response:** We thank the reviewer for the positive assessment of our revised manuscript. We greatly appreciate the reviewer’s constructive comments throughout the review process, which have helped us refine our conclusions and improve the clarity and rigor of the manuscript.

**Part II – Major Issues: Key Experiments Required for Acceptance**

Reviewer #2: (No Response)

Reviewer #3: none

**Part III – Minor Issues: Editorial and Data Presentation Modifications**

Please use this section for editorial suggestions as well as relatively minor modifications of existing data that would enhance clarity.

Reviewer #2: Line 262, Figure Legend S4. It is unclear what reads/copies refers to. Based on the response to reviewer 3, I assume that this number is a read counts normalized to viral levels based on qPCR but could not find these details. The authors should therefore specifically mentioned which approach was used for normalizing the small RNA data.

**Response:** We thank the reviewer for pointing out this lack of clarity. We agree that the normalization procedure used for Figure S4 was not sufficiently described in the previous version. We have now explicitly described the normalization approach in the Methods and revised the corresponding description in the Results accordingly. Specifically, the raw vsiRNA read count for each virus was divided by the corresponding viral RNA copy number determined by absolute qPCR quantification for each treatment group. The resulting reads/copies ratio was used to represent vsiRNA abundance relative to viral RNA abundance. Please refer to the revised Result, Line 265, and Methods, Lines 625-629.

Line 294-299. It is unclear to me why DCR2 single knockdown was tested for miRNAs and the triple knockdown for piRNAs. There appears no rational choice, why miRNA profiles for example are not analyzed in triple knockdown conditions. Moreover, the piRNA profile appears to include all types of genomic small RNAs, including miRNAs. The materials and method section, indeed indicates that small RNAs were mapped to genome and EVEs, an approach that is expected to include non-piRNA reads. The authors should state the purpose of this analysis and I would recommend them to show the total size profile in both the DCR2 and triple knockdown condition. The 21-23 peak in these plots will be dominated by microRNAs and the 25-30nt peak represent piRNA-sized reads.

**Response:** We thank the reviewer for this helpful comment. We agree that the rationale and presentation of the previous analysis were not sufficiently clear or consistent. We first clarify that the miRNA size profiles in our previous analysis were generated specifically from reads assigned to identified miRNAs, rather than by classifying all 21-23 nt small RNAs as miRNAs. To enable a direct comparison between knockdown conditions, we have now analyzed the size distribution of identified miRNAs under both the *DCR2* single-knockdown and *DCR2*/*AGO2*/*R2D2* triple-knockdown conditions.

We also agree that the previous “piRNA” profile was not sufficiently specific, because small RNAs mapped to the genome or endogenous viral element sequences may include RNA species other than bona fide piRNAs. We have therefore removed this ambiguous presentation and replaced it with an analysis of the total clean small RNA population. The purpose of this analysis was to determine whether *DCR2* single knockdown or *DCR2*/*AGO2*/*R2D2* triple knockdown broadly altered the overall size distribution of small RNAs. Following the reviewer’s recommendation, we analyzed all quality-controlled small RNA reads of 18-30 nt under both knockdown conditions. In both cases, the overall size-distribution patterns were broadly maintained relative to the corresponding controls, with a prominent 22-nt peak and an additional population of reads in the 25-30 nt range. These analyses are now presented in the revised Supplemental Figure S8, and the corresponding descriptions in the Results (Lines 299-309), Methods (Lines 617-619) and figure legend (Lines 1086-1093) have been revised accordingly.

Line 300 – 301. The statement ‘To directly assess the functional contributions of DCR1, DCR2, and AGO2 to RNAi 301 pathway activity’ implies that the previous knockdown experiments were inferior and that the RNAi-of-RNAi setup provides additional functional insights into the role of DCR1, DCR2, and AGO2. This is not supported and this introductory statement should be changed.

**Response:** We thank the reviewer for this helpful comment. We agree that the previous wording could imply that the RNAi-of-RNAi assay provides a more direct functional assessment than the preceding knockdown experiments. We have therefore revised this introductory statement to avoid such an implication. The revised text now reads: “To further examine RNAi pathway activity following knockdown of DCR1, DCR2, and AGO2, we performed an RNAi-of-RNAi assay in third-instar *L. striatellus*.” Please refer to Lines 310-311 of the revised manuscript.

Line 438-448. This section should be revisited, as the paper now includes an analysis of vsiRNAs normalized to viral RNA abundance.

**Response:** We thank the reviewer for pointing this out. We have revised the Discussion to incorporate the vsiRNA analysis normalized to viral RNA abundance. Specifically, we now note that the vsiRNA reads/copies ratio was significantly reduced following *Ls_AGO2* knockdown, whereas no significant difference was observed following *Ls_DCR2* or *Ls_R2D2* knockdown compared with the control. The corresponding interpretation has been revised accordingly. Please refer to Lines 451-471 in the revised manuscript.

Line 466-467. Rephrase: “Identifying this alternative nuclease represents an important direction for future research.” > Identifying a putative alternative nuclease would represent an important direction for future research.

**Response:** We thank the reviewer for this suggestion. The text has been revised accordingly. Please refer to Line 491 in the revised manuscript.

Textual recommendations

Line 14-17: I recommend referring to multiple small RNA silencing pathways rather than suggesting that there is a single silencing pathway that operates through multiple branches.

**Response:** We thank the reviewer for this helpful suggestion. We agree that miRNA, siRNA, and piRNA are more appropriately described as distinct small RNA silencing pathways rather than as branches of a single pathway. We have therefore revised the text to state: “The small silencing RNA pathways are crucial post-transcriptional gene silencing mechanisms involved in antiviral defense, gene regulation, and transposon suppression. In insects, the three main pathways are the microRNA (miRNA), Piwi-interacting RNA (piRNA), and small interfering RNA (siRNA) pathways.” Please refer to Lines 14-17 in the revised manuscript.

Line 92, 112: Diptera insects > Dipteran insects

**Response:** Done (Lines 82 and 92).

Line 99-101: Grammar flaws. Please revise sentence structure.

**Response:** We thank the reviewer for pointing out this grammatical issue. We have revised the sentence structure for clarity. The revised text now reads: “The DCR2–R2D2 heterodimer plays critical roles in both siRNA production and the loading of siRNAs onto AGO2. R2D2 contains two dsRNA-binding domains (RBD1 and RBD2). In *Drosophila*, dsRNA binds to the helicase domain of DCR2, whereas siRNA binds to R2D2 and is subsequently loaded onto AGO2 with the aid of the Hsc70/Hsp90 chaperone machinery (39).” Please refer to Lines 97-102 in the revised manuscript.

Line 119: besides RNAi > besides mediating antiviral RNAi

**Response:** Done (Line 120).

Line 133: it should be mentioned which gene was edited.

**Response:** We thank the reviewer for this comment. We have revised the text to explicitly state that DCR2 was the gene targeted by CRISPR/Cas9-mediated gene editing in *L. striatellus*. Please refer to Lines 134-135 in the revised manuscript.

In general, the manuscript still contains a number of language-related issues. I trust that these will be identified and corrected as the paper is moved forward.

**Response:** We thank the reviewer for this comment. We have carefully proofread the entire manuscript and corrected language-related issues throughout, including subject-verb agreement, singular/plural usage, verb tense, articles and prepositions, sentence structure, and terminology. We also revised several awkward expressions in the Results and Methods and standardized wording and formatting in the figure legends. For example, we corrected expressions such as “genes expression” to “gene expression”, “in animal” to “in animals”, “small silencing RNA pathways mechanisms” to “mechanisms of small silencing RNA pathways”, “at 12 days post injected” to “at 12 days post-injection”, “the 21-nt length peak was also disappeared” to “the 21-nt peak also disappeared”, and “our results shown” to “our results showed”. The Data Availability and Competing Interests statements were also corrected for grammar and clarity.

Reviewer #3: none
