# Supplementary material for: Functional dissection of conserved RNAi pathways in antiviral immunity of several hemipteran insects
Source: PLoS Pathog. 2026 Oct 30;22(10):e1014753. doi: 10.1371/journal.ppat.1014753 (PMC13734579; doi:10.1371/journal.ppat.1014753)
Supplement: Peer review history

# Peer review: Decision Letter 0

**DOI:** 10.1371/journal.ppat.1014753.r001

**Authors:** Alexander E. Gorbalenya, Barry Rockx

28 Nov 2025

Functional dissection of conserved RNAi pathways reveals Dicer2-independent antiviral defense against RNA viruses in hemipteran insects

PLOS Pathogens

Dear Dr. Li,

Thank you for submitting your manuscript to PLOS Pathogens. After careful consideration, we feel that it has merit but does not fully meet PLOS Pathogens's publication criteria as it currently stands. Therefore, we invite you to submit a revised version of the manuscript that addresses the points raised during the review process.

Please submit your revised manuscript by Jan 27 2026 11:59PM. If you will need more time than this to complete your revisions, please reply to this message or contact the journal office at plospathogens@plos.org. When you're ready to submit your revision, log on to https://www.editorialmanager.com/ppathogens/ and select the 'Submissions Needing Revision' folder to locate your manuscript file.

Please include the following items when submitting your revised manuscript:

* A rebuttal letter that responds to each point raised by the editor and reviewer(s). You should upload this letter as a separate file labeled 'Response to Reviewers'. This file does not need to include responses to any formatting updates and technical items listed in the 'Journal Requirements' section below.

* A marked-up copy of your manuscript that highlights changes made to the original version. You should upload this as a separate file labeled 'Revised Manuscript with Track Changes'.

* An unmarked version of your revised paper without tracked changes. You should upload this as a separate file labeled 'Manuscript'.

If you would like to make changes to your financial disclosure, competing interests statement, or data availability statement, please make these updates within the submission form at the time of resubmission. Guidelines for resubmitting your figure files are available below the reviewer comments at the end of this letter.

We look forward to receiving your revised manuscript.

Kind regards,

Barry Rockx

Academic Editor

PLOS Pathogens

Alexander Gorbalenya

Section Editor

PLOS Pathogens

Editor-in-Chief

PLOS Pathogens

orcid.org/0000-0003-2946-9497

Editor-in-Chief

PLOS Pathogens

orcid.org/0000-0002-7699-2064

**Additional Editor Comments:**

This manuscript confirms an antiviral function of RNAi in Laodelphax striatellus. The reviewers state that while the manuscript is interesting, this concept is not novel as this has been shown in many other insect species, and also in this species there is already published evidence for an antiviral function of RNAi. The novelty comes from the claim of DCL2 independent viral siRNA production. However, reviewers are not convinced that your results definitively show this. Several suggestions for additional experiments to support this hypothesis, have been made and should be considered. As it stands now, the conclusions are not (fully) supported by the results.

**Journal Requirements:**

1) We do not publish any copyright or trademark symbols that usually accompany proprietary names, eg ©,  ®, or TM  (e.g. next to drug or reagent names). Therefore please remove all instances of trademark/copyright symbols throughout the text, including:

- ® on page: 28.

- TM on page: 28..

2) Please upload all main figures as separate Figure files in .tif or .eps format. For more information about how to convert and format your figure files please see our guidelines:

https://journals.plos.org/plospathogens/s/figures

3) Some material included in your submission may be copyrighted. According to PLOSu2019s copyright policy, authors who use figures or other material (e.g., graphics, clipart, maps) from another author or copyright holder must demonstrate or obtain permission to publish this material under the Creative Commons Attribution 4.0 International (CC BY 4.0) License used by PLOS journals. Please closely review the details of PLOSu2019s copyright requirements here: PLOS Licenses and Copyright. If you need to request permissions from a copyright holder, you may use PLOS's Copyright Content Permission form.

Please respond directly to this email and provide any known details concerning your material's license terms and permissions required for reuse, even if you have not yet obtained copyright permissions or are unsure of your material's copyright compatibility. Once you have responded and addressed all other outstanding technical requirements, you may resubmit your manuscript within Editorial Manager.

Potential Copyright Issues:

- Please confirm (a) that you are the photographer of Figures 2 and 5., or (b) provide written permission from the photographer to publish the photo(s) under our CC BY 4.0 license.

- Figures 1, 6, and S7.. Please confirm whether you drew the images / clip-art within the figure panels by hand. If you did not draw the images, please provide (a) a link to the source of the images or icons and their license / terms of use; or (b) written permission from the copyright holder to publish the images or icons under our CC BY 4.0 license. Alternatively, you may replace the images with open source alternatives. See these open source resources you may use to replace images / clip-art:

- https://commons.wikimedia.org

- https://openclipart.org/.

4) Please amend your detailed Financial Disclosure statement. This is published with the article. It must therefore be completed in full sentences and contain the exact wording you wish to be published.

- State the initials, alongside each funding source, of each author to receive each grant. For example: "This work was supported by the National Institutes of Health (####### to AM; ###### to CJ) and the National Science Foundation (###### to AM)."

- State what role the funders took in the study. If the funders had no role in your study, please state: "The funders had no role in study design, data collection and analysis, decision to publish, or preparation of the manuscript.".

If you did not receive any funding for this study, please simply state: u201cThe authors received no specific funding for this work.u201d

**Reviewers' Comments:**

Reviewer's Responses to Questions

**Part I - Summary**

Please use this section to discuss strengths/weaknesses of study, novelty/significance, general execution and scholarship.

Reviewer #1: This manuscript investigates the three canonical insect RNAi pathways in the hemipteran L. striatellus and related species. The authors report that antiviral control of several insect-specific RNA viruses depends on AGO2 but is independent of DCL2. CRISPR/Cas9 knockout of DCL2 results in embryonic lethality, while heterozygous mutants exhibit delayed development. These findings lead the authors to conclude that in Hemiptera, DCL2 is essential for development but dispensable for antiviral RNAi. The study is conceptually significant because it challenges the canonical model of DCL2 mediated siRNA biogenesis established in Diptera. However, several mechanistic and data transparency issues must be addressed before the manuscript can be considered for publication.

Reviewer #2: The research manuscript ‘Functional dissection of conserved RNAi pathways reveals Dicer2-independent antiviral defense against RNA viruses in hemipteran insects’ by Zhang et al. characterizes small silencing RNA pathways in the hemipteran insect Laodelphax striatellus. The authors identify orthologous genes at the heart of the microRNA, piRNA and siRNA pathways and, upon depletion of these core factors, they analyze physiological traits including fecundity and fertility, small RNA populations, and virus replication. In line with literature from other insect models, they demonstrate the microRNA pathway to be important for survival and developmental phenotypes. Likewise, knockdown of Piwi, a central piRNA binding protein in the piRNA pathway results in reduced fertility. Knockdown of several, but not all, small silencing RNA pathway-associated proteins results in decrease of the corresponding class of small RNA. Antiviral activity was demonstrated to be associated with the siRNA pathway only. Strikingly, the authors propose that the antiviral siRNA pathway in Laodelphax striatellus and two additional non-model insect species acts independently of Dicer-2, a core component of the siRNA machinery. An attempt to validate this significant finding using a full genetic knockout unfortunately results in lethality of homozygous mutants.

The descriptive part of the study is mostly solid and the data on fecundity and fertility upon knockdown of individual proteins form the distinct small silencing RNA pathway knockdown is largely coherent with findings in other insect models. Mechanistically, the study remains superficial, especially on the characterization of microRNA and piRNA populations. These small RNA classes could have been analyzed in greater depth for example by quantifying individual microRNAs or by checking the piRNA pool for the commonly seen nucleotide biases.

The observation of Dicer-2 independent siRNA production is a significant claim and the burden of proof for this observation is high. In its current state, the study does not provide convincingly firm evidence to support this conclusion. As the authors have noted, RNAi-mediated knockdown of the RNAi machinery is inherently tricky and has resulted in sub-functional protein depletion in other models. With the data presented, the authors cannot exclude that residual Dicer-2 protein activity is able to generate siRNAs in sufficient numbers to guide Ago2 to silence viral transcripts. In fact, siRNA populations in wildtype planthoppers do present the well-described features of siRNA populations in terms of size and similar number of reads from positive and negative strands. To me this strongly suggests that a Dicer protein is responsible for the siRNA production. I appreciate the attempt of the authors to address this important aspect with a genetic knockout model, but as these data are still inconclusive due to lethality, the manuscript lacks the final proof for Dicer-2 independent siRNA biogenesis.

N.B. The same argument can be made for the absence of a fertility/piRNA phenotype upon Ago3 knockdown which can still be attributed to residual protein activity. Therefore, the claim that ‘Ls_Piwi, but not Ls_AGO3, is actively involved in the piRNA pathway of L. striatellus’ (lines 246-7) still requires more rigorous experimental validation.

Reviewer #3: In this manuscript, the authors analyze the role of small RNA pathways in antiviral defense in hemipteran insects. Through a knockdown approach, they find that knockdown of the miRNA (Ago1 and Dcl1) and piRNA (Piwi and Ago3) pathway genes affect fecundity and development rates. Knockdown of Ago2 led to an increase in viral replication and consequently an increase in viral siRNA numbers. Surprisingly, knockdown of Dcl2 did not affect siRNA levels and viral replication. This observation is the basis for the main conclusion of the title of Dicer 2 (DCL2) independent antiviral defense. Next, the authors generated a Dicer2 knockout using CRISPR/Cas9 and found that this led to a developmental arrest and no adult homozygotes were obtained. Finally, the authors performed Ago2 and DCL2 knockdowns in other insects and again find that Ago2 affects viral replication whereas DCL2 does not.

Overall, the manuscript supports the critical role of RNAi in antiviral defense and suggests a role of siRNA pathway in development in Laodelphax striatellus – the small brown planthopper, a vector for several plant viruses. These findings are in line with findings in other organisms and previous reports of in this species (https://doi.org/10.1186/s12870-018-1438-7; https://doi.org/10.3390/v13081591), and therefore not unexpected. The title suggests that the authors consider the conclusion that antiviral RNAi is independent of DCL2 the main novelty of the manuscript, but in my opinion this conclusion is not warranted based on the provided data (see below).

**********

**Part II – Major Issues: Key Experiments Required for Acceptance**

Please use this section to detail the key new experiments or modifications of existing experiments that should be absolutely required to validate study conclusions.

Generally, there should be no more than 3 such required experiments or major modifications for a "Major Revision" recommendation. If more than 3 experiments are necessary to validate the study conclusions, then you are encouraged to recommend "Reject".

Reviewer #1: 1. The mechanistic evidence supporting DCL2 independent vsiRNA biogenesis remains incomplete. The authors may consider expressing L. striatellus DCL2 and DCL1 in vitro, purifying the recombinant proteins, and performing dsRNA cleavage assays. Comparing the enzymatic activities of these RNase III–like proteins would provide direct biochemical evidence of their functions and clarify whether DCL2 is indeed dispensable for vsiRNA production in this species.

2. The observation that AGO2 knockdown increases both viral RNA and vsiRNA counts is expected if higher viral replication produces more precursor dsRNA. This phenomenon by itself does not reveal the nuclease responsible for vsiRNA generation. The authors should normalize vsiRNA abundance to viral genome copy numbers and analyze vsiRNA mapping profiles to distinguish genuine Dicer processed products from nonspecific degradation fragments, thereby providing stronger mechanistic support for their conclusion.

3. The manuscript currently lacks compliant data deposition information. According to PLOS Pathogens policy, all sequencing data supporting the findings must be deposited in an appropriate public repository, and accession numbers must be clearly provided in the data availability statement. The present statement that all data are available in the main text or Supplementary Materials does not meet this requirement. The authors should ensure that all raw and processed datasets are publicly accessible and that repository accession numbers are included before resubmission.

4. Several figures appear to rely on unadjusted two-tailed Student’s t-tests across multiple comparisons. The authors should specify whether each dataset represents biological or technical replicates, provide the exact sample size (n), test assumptions of normality and variance, and apply appropriate multiple comparison corrections where necessary.

Reviewer #2: The central claim, included in the title, that (selected) hemipteran insects produce siRNAs in a Dicer-2 independent fashion requires firm experimental proof. For this purpose, a model with validated functional knockout of dicer-2 activity must be achieved. While I recognize that this will be difficult to obtain, given the data presented here, this remains the ultimate experimental evidence to support this remarkable conclusion. If these data cannot be generated, the conclusions should be toned down significantly, in which case much of the general interest and novelty of this manuscript is reduced.

Another way forward would be identifying the proposed unknown nuclease, other than Dicer-2, that the authors suspect to be involved in siRNA biogenesis. Given the siRNA characteristics, Dicer-1 might be a logical candidate. In this line of reasoning, I was wondering if the authors have considered the possibility of redundant functions of Dicer 1 and Dicer 2 that could mask the siRNA phenotype of Dicer-2 knockdown. This could for example be tested by co-injecting dsRNA targeting both Dicer proteins at the same time.

Reviewer #3: - The conclusion that antiviral RNAi is independent of DCL2 is based on the observation that viral small RNAs are not reduced upon DCL2 knockdown. This conclusion is fully based on a knockdown approach (using RNAi to knockdown RNAi genes), which inevitably is incomplete: Even if RNA levels are strongly reduced (as shown by the authors), residual levels of DCL2 protein may be sufficient to process viral dsRNA. Another explanation for the lack of a phenotype may be based on the use of persistent virus infections. In such a setting, viral siRNAs are already preexisting and loaded in Argonaute 2 before knockdown and it is well-established that Argonaute-loaded small RNAs can have long half-lives. It is therefore possible that a knockdown of Dicer may therefore not significantly affect siRNA profiles.

- To more comprehensively analyze the DCL2 knockdown data, please show siRNA size profiles upon DCL2/AGO2/R2D2 knockdown in Figure 4 (akin to Figure 3A-b). Also, it would be useful to use the data to identify sources of endo-siRNAs and thus validate that DCL2 knockdown functionally affects siRNA levels.

- I am not aware of any other experimental systems in which viral siRNAs are produced in a Dicer-independent manner. The authors suggest that there may be an other, yet unidentified, DCL2 like enzyme that produces siRNAs. In that case, the lack of a phenotype for DCL2 knockdown may be a simple, unfortunate consequence of an incomplete reference genome/genome annotation (and the finding would be less remarkable). The authors may want to comment on the completeness of the reference genome.

- Has potential redundancy between DCL1 and DCL2 been assessed via a double knockdown? This would still be an important experiment to perform.

- I applaud the authors efforts to produce a DCL2 knockout using CRISPR. Unfortunately, the animals did not survive into adulthood to assess antiviral activity at this stage. Yet, some early stage homozygous nymphs were recovered. Are the persistent infections already detectable at this stage? If so, the authors should analyze viral RNA levels and viral small RNA profiles to solidify their conclusion.

- The authors report a role for DCL2/RNAi in development based on the observation that no homozygote adults were found. The mechanism by which RNAi affects development has not been studied. I would encourage the authors to at least perform small RNA profiles in early stage animals to study whether endogenous siRNAs are lost in these animals to provide a first glimpse on a mechanism by which RNAi controls development.

- Small RNA read counts are very low in Figure 3B compared to figure 3A. Moreover, miRNAs are notably low compared to figure 3A. It seems that something went wrong in the sequencing or data analyses. Please double check and/or repeat the sequence/analyses. Also, reads are plotted as negatives. Am I correct in assuming that this reflects the “sense” and “antisense” strands of the genome? If so, this would not be meaningful as genes and transposable elements can be in both orientations on the genome. I suggest plotting similarly to Figure 3A (i.e., only total counts).

- Effect of miRNA pathway genes are only analyzed for their effects on total small RNA populations (Figure 3A). I would encourage to also analyze small RNA reads mapping to miRNAs directly. Should miRNAs not be annotated in the genome, it would be worthwhile using their data to annotate miRNAs.

**********

**Part III – Minor Issues: Editorial and Data Presentation Modifications**

Please use this section for editorial suggestions as well as relatively minor modifications of existing data that would enhance clarity.

Reviewer #1: The figure legends should indicate sample sizes, exact n values, and clarify whether each dot represents an independent biological replicate in Figures 2–6. Providing these details will improve transparency and allow readers to better evaluate data variability and robustness.

The Methods section should be expanded to include essential experimental parameters such as the concentration of dsRNA used, the developmental stages at which injections were performed, and the precise sampling time points after injection.

Reviewer #2: Minor re-analysis:

The phylogenetic analysis should be revisited. In its current state, the protein-by-protein analysis is geared towards replicating the evolutionary distance of the organisms included. The analysis would become more informative if the clustering would be less supervised a priori, for instance by making a phylogenetic tree for the Argonaute proteins and separate trees for PIWI proteins and Dicer proteins, similar to the study ‘Comparative genomics of small RNA regulatory pathway components in vector mosquitoes’ (doi: 10.1186/1471-2164-9-425). For completeness, the PIWI protein Aubergine from Drosophila should be included in the analysis.

Editorial suggestions:

I wonder why the authors use the abbreviation DCL1 and DCL2 for Dicer-1 and Dicer-2, respectively. DCL usually refers to Dicer-like proteins from plants. The abbreviation in the insect field is Dcr1 and Dcr2 and should be used here accordingly.

Strictly speaking, the term ‘RNA interference’ should be used as a synonym for the siRNA pathway. (doi: 10.1038/nrg2504). I therefore, recommend using the term ‘small silencing RNA pathways’ when referring to miRNA, piRNA, and siRNA as a whole.

Lines 62-63: All small silencing RNAs discussed in this study bind to Argonaute proteins. piRNA distinguish themselves from microRNAs and siRNAs by binding to the PIWI clade of Argonaute proteins, which should be specified in the statement.

Line 64: siRNAs do not target exogenous or endogenous dsRNA. They are made from dsRNA and target single stranded transcripts

Reviewer #3: - Number of observations and replicates should be reported in the legends for all figures. Error bars need to be defined (SEM or STD).

- Fig. 2: Pictures of the molting defects require higher resolution figures and quantification. Also, for the reader without experience with this animal, it may be useful to briefly describe how this is visible from the figure.

- Fig. 2: extremely minor differences which large overlaps between the range of data are noted as statistically significant. Please verify that this is correct and that the correct statistical test has been used.

- For consistency with piRNA and siRNA data, please also show the development duration of nymphs for Ago1 and DCL1.

- Fig. 2, the legend reports that ANOVA and T tests were used in this figure. However, this is not appropriate for survival curves. Please use an appropriate test to analyze the survivals.

- Figure 3. Define the red and black bars in the legends.

- Fig. 3C-G: How similar are the EVE sequences? Can the small RNAs be unambiguously mapped to the individual elements?

- T test is not appropriate to analyze the data in Figure S4 as it assumes a normal distribution, and the data do not seem to be normally distributed. I would also advice the authors to verify normal distributions for other figures in which T tests and ANOVA’s were used.

- Please, consistently show individual data points in all figures showing bar graphs. Also, ensure consistency in plotting (e.g., in Fig. 5C dot plots are shown whereas in Fig. 5A/B bargraphs were used for the same data types).

- Line 245-247: The conclusion that Ago3 is not involved in the piRNA pathway cannot be simply drawn from the number of piRNAs. If there are only few ping-pong derived piRNAs in this species, no strong phenotype is expected for Ago3 knockdown. Indeed, Ago3 knockdown shows a phenotype in hatch rates in Fig S3, suggesting an essential role of Ago3. Rephrase the conclusion. Likewise, line 410 is not supported by the data.

Suggestions for data and text presentation:

- Fig 2B, d: define stage of the animals in the legend.

- Define the green dashed lines in Figure 4

- Fig. 1. The Drosophila PIWI gene Aubergine is lacking from the phylogenetic tree. I suggest adding them. Also, Apis Ago1 and Ago2 seem to be lacking from the trees. While not essential, it may be useful to add for consistency.

- Fig. 1. Define the scale bar

- Fig. 5C: the figure is a bit unclear to me. What are the 6 dots. I would advise the authors to also report the number of offspring for the different genotypes to make the figure more informative.

- Fig. 5D and 5E: The figure would benefit from a description for what is shown in the figure for people not working with these insects. Also, a wildtype control should be provided as comparison.

- Fig. 5D and 5E: the electropherogram of the homozygotes adds little information and could be deleted.

- Table S2: as I understand the table, it seems that the numbers report the number of animals with the indicated genotypes and not the “failure to hatch” as the title suggests. Please correct.

- Fig. S7: please report how many animals have been analyzed. Also, in my opinion, the symbols in the insect and under the graph add little information.

- Line 51: “it is triggered by dsRNA” is not entirely correct for piRNA pathway. Please correct.

- Line 63: “siRNAs are dsRNAs around 20-25 nt in length, targeting either exogenous or endogenous dsRNA”. I suggest rephrasing as siRNAs are not targeting dsRNA, rather they are derived from dsRNA.

- Line 192: please check the grammar.

- Line 267-268: a brief introduction of these viruses may be useful.

- Line 333 onwards: from the text it is unclear which virus was found in which species. Also, it is unclear how these viruses were identified in the first place. Please provide this information.

**********

PLOS authors have the option to publish the peer review history of their article (what does this mean?). If published, this will include your full peer review and any attached files.

If you choose “no”, your identity will remain anonymous but your review may still be made public.

**Do you want your identity to be public for this peer review?**  For information about this choice, including consent withdrawal, please see our Privacy Policy.

Reviewer #1: No

Reviewer #2: No

Reviewer #3: No

[NOTE: If reviewer comments were submitted as an attachment file, they will be attached to this email and accessible via the submission site. Please log into your account, locate the manuscript record, and check for the action link "View Attachments". If this link does not appear, there are no attachment files.]

**Figure resubmission:**

**Reproducibility:**

?>

# Author comment: Author response to Decision Letter 1

**DOI:** 10.1371/journal.ppat.1014753.r002

29 Mar 2026

Attachment Submitted filename: Response to Reviewers.docx

# Peer review: Decision Letter 1

**DOI:** 10.1371/journal.ppat.1014753.r003

**Authors:** Alexander E. Gorbalenya, Barry Rockx

29 May 2026

Functional dissection of conserved RNAi pathways reveals Dicer2-independent antiviral defense against RNA viruses in several hemipteran insects

PLOS Pathogens

Dear Dr. Li,

Thank you for submitting your manuscript to PLOS Pathogens. After careful consideration, we feel that it has merit but does not fully meet PLOS Pathogens's publication criteria as it currently stands. Therefore, we invite you to submit a revised version of the manuscript that addresses the points raised during the review process.

Please submit your revised manuscript by Jul 28 2026 11:59PM. If you will need more time than this to complete your revisions, please reply to this message or contact the journal office at plospathogens@plos.org. When you're ready to submit your revision, log on to https://www.editorialmanager.com/ppathogens/ and select the 'Submissions Needing Revision' folder to locate your manuscript file.

Please include the following items when submitting your revised manuscript:

* A letter that responds to each point raised by the editor and reviewer(s). You should upload this letter as a separate file labeled 'Response to Reviewers'. This file does not need to include responses to any formatting updates and technical items listed in the 'Journal Requirements' section below.

* A marked-up copy of your manuscript that highlights changes made to the original version. You should upload this as a separate file labeled 'Revised Manuscript with Track Changes'.

* An unmarked version of your revised paper without tracked changes. You should upload this as a separate file labeled 'Manuscript'.

As the corresponding author, your ORCID iD is verified in the submission system and will appear in the published article. PLOS supports the use of ORCID, and we encourage all coauthors to register for an ORCID iD and use it as well. Please encourage your coauthors to verify their ORCID iD within the submission system before final acceptance, as unverified ORCID iDs will not appear in the published article. Only the individual author can complete the verification step; PLOS staff cannot verify ORCID iDs on behalf of authors.

We look forward to receiving your revised manuscript.

Kind regards,

Barry Rockx

Academic Editor

PLOS Pathogens

Alexander Gorbalenya

Section Editor

PLOS Pathogens

Editor-in-Chief

PLOS Pathogens

Editor-in-Chief

PLOS Pathogens

orcid.org/0000-0002-7699-2064

**Additional Editor Comments (if provided):**

Thank you for your revised manuscript and the additional data provided to support central your claim that RNAi operates independently of Dicer2.

The reviewers all agree that despite your efforts, the current experiments do not directly identify the enzyme responsible for vsiRNA generation. Additional experiments are suggested by the reviewers, to provide this information more definitively.

As an alternative, the reviewers also provide you the option to tone down your conclusion of Dicer2 independence throughout the manuscript, including the title.

**Journal Requirements:**

If the reviewer comments include a recommendation to cite specific previously published works, please review and evaluate these publications to determine whether they are relevant and should be cited. There is no requirement to cite these works unless the editor has indicated otherwise.

1) Your Response to Reviewers should be uploaded as a single, individual file. Please remove your Response to Reviewers from your Cover Letter.

2) Please provide an Author Summary. This should appear in your manuscript between the Abstract (if applicable) and the Introduction, and should be 150-200 words long. The aim should be to make your findings accessible to a wide audience that includes both scientists and non-scientists. Sample summaries can be found on our website under Submission Guidelines:

https://journals.plos.org/plospathogens/s/submission-guidelines#loc-parts-of-a-submission

**Reviewers' Comments:**

Reviewer's Responses to Questions

**Part I - Summary**

Please use this section to discuss strengths/weaknesses of study, novelty/significance, general execution and scholarship.

Reviewer #1: I appreciate the substantial effort made in revising the manuscript. The additional experiments have strengthened the study and address several of my initial concerns.

However, I remain concerned that the central claim is still overstated. The manuscript repeatedly refers to DCR2-independent vsiRNA biogenesis. Based on the current data, this conclusion is stronger than what can be directly supported. The results demonstrate that DCR2 is not required or not limiting in the tested system, but they do not definitively exclude a contribution of DCR2 to vsiRNA production at the mechanistic level. In particular, residual DCR2 activity, partial redundancy, or the presence of additional nucleases cannot be ruled out. The current experiments do not directly identify the enzyme responsible for vsiRNA generation.

I therefore recommend that the authors moderate the wording throughout the manuscript. More cautious phrasing such as DCR2 is dispensable under the tested conditions, or vsiRNA production is not detectably affected by DCR2 depletion, would better reflect the data.

Reviewer #2: In the revised manuscript “Functional dissection of conserved RNAi pathways reveals Dicer2-independent antiviral defense against RNA viruses in several hemipteran insects” by Zhang et al. the authors have performed additional experiments to support their central claim that RNAi operates independently of Dicer2 in four hemipteran species. The two critical additions are an RNAi-of-RNAi assay and analysis of virus replication in heterozygous mutants. However, both of these assays do not address the major concern of residual protein activity. I disagree with the reviewers that the tubulin RNAi reporter assay firmly proofs Dicer2 independent RNAi ‘regardless of residual protein levels’ (line 313). Incomplete DCR2 depletion may still generate sufficient levels of tub siRNAs which may explain silencing of tubulin RNA as observed by qPCR in supplemental figure 7B. The conceptual difference compared to assays reported in figure 4 is that the target in this assay is not a replicating viral RNA but a cellular transcript. Yet, this change in experimental setup does not address the concern of residual protein activity. Likewise, heterozygous DCR2 mutants intrinsically have remaining DCR2 protein expression. Therefore, my initial concern remains, that the high burden of proof for DCR2-independent RNAi has not been met by the revised manuscript.

Reviewer #3: This is a revision of a manuscript addressing the role of RNAi in antiviral defense in hemipteran insects. My main concern on the first version of the manuscript (and that of the two other reviewers) was that the conclusion of Dicer-2 independent antiviral defense was not supported by the data. The authors have performed additional experiments to try to provide stronger support.

**********

**Part II – Major Issues: Key Experiments Required for Acceptance**

Please use this section to detail the key new experiments or modifications of existing experiments that should be absolutely required to validate study conclusions.

Generally, there should be no more than 3 such required experiments or major modifications for a "Major Revision" recommendation. If more than 3 experiments are necessary to validate the study conclusions, then you are encouraged to recommend "Reject".

Reviewer #1: (No Response)

Reviewer #2: A genetically clean assay that genuinely addresses the concern of residual protein activity is needed to convincingly show DCR2-independent RNAi.

Reviewer #3: Unfortunately, I remain unconvinced of the main conclusion of Dicer indendence:

1) The main argument remains centered on the use of an RNAi approach to knock down RNAi genes. The extra experiment provided in Figure S7 actually strengthens my concern: prior knockdown of Dicer 1 and Dicer 2 do not affect silencing of tubulin by dsRNA. The authors are not correct in their rebuttal with their statement in the text “This functional result is independent of the question of how much residual DCR2 protein remains”. RNAi of Dicer2 is critically dependent on the amount of Dicer2 protein remaining at the time of the second (tubulin specific) dsRNA. Although the authors refer to work that RNAi-of-RNAi worked in other insects, it is likely that there are gene specific differences in RNAi-of-RNAi efficiency, which may be related to differences in protein stability and turnover. The experiment of Figure S7 indicates that it had not been possible to sufficiently knock down Dicer2 to affect RNAi, and it is therefore not surprising that no viral phenotypes have been obtained.

2) While the combined knockdown does address potential redundancy between Dicer1 and Dicer2, but the experiment suffers from the limitation of using RNAi to knock down RNAi genes.

3) The experiment with heterozygous Dicer mutants (no antiviral phenotype despite reduced Dicer2 expression) is interesting, but not conclusive: Both Dicer and Argonaute are multi-turnover enzymes and reduced siRNA levels in heterozygous Dicer2 mutants may already suffice to mediate effective antiviral RNAi. Hence, there is unlikely to be a linear relation between Dicer expression and RNAi efficiency.

The most parsimonous conclusion of these experiments is that RNAi of RNAi simply does not work for Dicer2 in this species and that results from such experiments cannot be used to conclude that antiviral defense is Dicer2 independent. An alternative conclusion would be that there is another, yet unknown, nuclease generating the viral siRNAs that are loaded in Argonaute 2. However, in their rebuttal the authors indicate that this is not likely as the reference genome is high-quality.

Overall, I appreciate the authors efforts to convince the reviewers of their conclusions, but I remain unconvinced of their main conclusion. To move this manuscript forward, I would propose that the authors tone down their conclusion of Dicer2 indepence throughout the manuscript.

Other comments:

- My original suggestion to use small RNA data to identify sources of endo-siRNAs and validate that DCR2 knockdown functionally affects siRNA levels has not been addressed, nor rebutted. This should still be done. In this regard, the authors should also provide the total size distribution of the Dicer2/Ago2 knockdown, akin to those provided for miRNA and piRNA genes (Fig. 3A(b) and 3B(b)).

- The author’s response to my earlier comment that “Small RNA read counts are very low in Figure 3B compared to figure 3A.” is not entirely convincing. In Panel 3A(b) the number of 22-nt small RNAs in dsGFP control is approximately 120,000 RPM whereas this was only ~1500 RPM in panel 3B(b). This discrepancy requires an explanation. Were reads normalized to total library size or to total genome mapped reads? Are there many non-mapping reads in the libraries of panel 3B(b)? It seems that there is a large proportion of reads not accounted for.

- Figure 1A: R2D2 and Dicer enzymes have a vastly different domain structure and the sequences are unlikely to align (or only by introducing may/large gaps in the sequence). The trees should be made separately for the R2D2 and Dicer genes.

- Figure 4: I agree with Reviewer 1 that the number of viral siRNAs in Ago2 knockdown should be normalized to the viral RNA levels. The authors indicate that RPM is a standard approach to normalize the data (which is correct), but the authors can divide these numbers by the relative viral RNA levels from the qPCR assay shown in the right panel of figure 4B. It is not sufficient to merely suggest normalization to viral levels in the discussion.

- The authors refer to custom Python script. For reproducibility, these script should be deposited, e.g. in GitHub.

**********

**Part III – Minor Issues: Editorial and Data Presentation Modifications**

Please use this section for editorial suggestions as well as relatively minor modifications of existing data that would enhance clarity.

Reviewer #1: (No Response)

Reviewer #2: My previous minor issues have been addressed appropriately.

Reviewer #3: - Page 32: typo: Eepression

- Fig S9: y axis: I would suggest replacing ‘infective ratio’ with ‘infection rates’ (as also used in the legend)

**********

PLOS authors have the option to publish the peer review history of their article (what does this mean?). If published, this will include your full peer review and any attached files.

If you choose “no”, your identity will remain anonymous but your review may still be made public.

**Do you want your identity to be public for this peer review?**  For information about this choice, including consent withdrawal, please see our Privacy Policy.

Reviewer #1: No

Reviewer #2: No

Reviewer #3: No

[NOTE: If reviewer comments were submitted as an attachment file, they will be attached to this email and accessible via the submission site. Please log into your account, locate the manuscript record, and check for the action link "View Attachments". If this link does not appear, there are no attachment files.]

**Figure resubmission:**

**Reproducibility:**

?>

# Author comment: Author response to Decision Letter 2

**DOI:** 10.1371/journal.ppat.1014753.r004

26 Jul 2026

Attachment Submitted filename: Response to Reviewers.R2.docx

# Peer review: Decision Letter 2

**DOI:** 10.1371/journal.ppat.1014753.r005

**Authors:** Alexander E. Gorbalenya, Barry Rockx

22 Sep 2026

PPATHOGENS-D-25-02414R2

Functional dissection of conserved RNAi pathways in antiviral immunity of several hemipteran insects

PLOS Pathogens

Dear Dr. Li,

Thank you for submitting your manuscript to PLOS Pathogens. After careful consideration, we feel that it has merit but does not fully meet PLOS Pathogens's publication criteria as it currently stands. Therefore, we invite you to submit a revised version of the manuscript that addresses the points raised during the review process.

Please submit your revised manuscript by Nov 21 2026 11:59PM. If you will need more time than this to complete your revisions, please reply to this message or contact the journal office at plospathogens@plos.org. When you're ready to submit your revision, log on to https://www.editorialmanager.com/ppathogens/ and select the 'Submissions Needing Revision' folder to locate your manuscript file.

Please include the following items when submitting your revised manuscript:

* A letter that responds to each point raised by the editor and reviewer(s). You should upload this letter as a separate file labeled 'Response to Reviewers'. This file does not need to include responses to any formatting updates and technical items listed in the 'Journal Requirements' section below.

* A marked-up copy of your manuscript that highlights changes made to the original version. You should upload this as a separate file labeled 'Revised Manuscript with Track Changes'.

* An unmarked version of your revised paper without tracked changes. You should upload this as a separate file labeled 'Manuscript'.

If you would like to make changes to your financial disclosure, competing interests statement, or data availability statement, please make these updates within the submission form at the time of resubmission. Guidelines for resubmitting your figure files are available below the reviewer comments at the end of this letter.

As the corresponding author, your ORCID iD is verified in the submission system and will appear in the published article. PLOS supports the use of ORCID, and we encourage all coauthors to register for an ORCID iD and use it as well. Please encourage your coauthors to verify their ORCID iD within the submission system before final acceptance, as unverified ORCID iDs will not appear in the published article. Only the individual author can complete the verification step; PLOS staff cannot verify ORCID iDs on behalf of authors.

We look forward to receiving your revised manuscript.

Kind regards,

Barry Rockx

Academic Editor

PLOS Pathogens

Alexander Gorbalenya

Section Editor

PLOS Pathogens

Sumita Bhaduri-McIntosh

Editor-in-Chief

PLOS Pathogens

orcid.org/0000-0003-2946-9497

Dominique Soldati-Favre

Editor-in-Chief

PLOS Pathogens

orcid.org/0000-0003-4156-2109

**Additional Editor Comments (if provided):**

Dear authors, we appreciate all the revisions you have performed to improve the manuscript. Overall the reviewers are positive, however they have identified a few minor comments to be addressed.

**Journal Requirements:**

1) We noticed that you used "unpublished data" in the manuscript. We do not allow these references, as the PLOS data access policy requires that all data be either published with the manuscript or made available in a publicly accessible database. Please amend the supplementary material to include the referenced data or remove the references.

If the reviewer comments include a recommendation to cite specific previously published works, please review and evaluate these publications to determine whether they are relevant and should be cited. There is no requirement to cite these works unless the editor has indicated otherwise.

[Note: HTML markup is below. Please do not edit.]

**Reviewers' Comments:**

Reviewer's Responses to Questions

**Part I - Summary**

Please use this section to discuss strengths/weaknesses of study, novelty/significance, general execution and scholarship.

Reviewer #2: I appreciate that the authors have decided to tone down the claim about DCR2-independent processing of siRNAs throughout the manuscript. In line with these changes, I encourage the authors to change the statement ‘implying that siRNA-mediated antiviral pathway in hemipteran insects might be distinct to that of Drosophila and mosquitoes.’ (line 270), which is still an overstatement, despite the careful phrasing. In my opinion, there is no need to make a direct comparison to other Dipteran insects, and I recommend keeping this statement descriptive focusing on the data obtained in this study.

Reviewer #3: This is a second revision of a manuscript analyzing the role of RNAi genes in antiviral defense in hemipteran insects. The authors have adjusted their initial conclusion of Dicer2 independent viral siRNA production, as requested by all reviewers. Current conclusions are now supported by the presented data.

**********

**Part II – Major Issues: Key Experiments Required for Acceptance**

Please use this section to detail the key new experiments or modifications of existing experiments that should be absolutely required to validate study conclusions.

Generally, there should be no more than 3 such required experiments or major modifications for a "Major Revision" recommendation. If more than 3 experiments are necessary to validate the study conclusions, then you are encouraged to recommend "Reject".

Reviewer #2: (No Response)

Reviewer #3: none

**********

**Part III – Minor Issues: Editorial and Data Presentation Modifications**

Please use this section for editorial suggestions as well as relatively minor modifications of existing data that would enhance clarity.

Reviewer #2: Line 262, Figure Legend S4. It is unclear what reads/copies refers to. Based on the response to reviewer 3, I assume that this number is a read counts normalized to viral levels based on qPCR but could not find these details. The authors should therefore specifically mentioned which approach was used for normalizing the small RNA data.

Line 294-299. It is unclear to me why DCR2 single knockdown was tested for miRNAs and the triple knockdown for piRNAs. There appears no rational choice, why miRNA profiles for example are not analyzed in triple knockdown conditions. Moreover, the piRNA profile appears to include all types of genomic small RNAs, including miRNAs. The materials and method section, indeed indicates that small RNAs were mapped to genome and EVEs, an approach that is expected to include non-piRNA reads. The authors should state the purpose of this analysis and I would recommend them to show the total size profile in both the DCR2 and triple knockdown condition. The 21-23 peak in these plots will be dominated by microRNAs and the 25-30nt peak represent piRNA-sized reads.

Line 300 – 301. The statement ‘To directly assess the functional contributions of DCR1, DCR2, and AGO2 to RNAi 301 pathway activity’ implies that the previous knockdown experiments were inferior and that the RNAi-of-RNAi setup provides additional functional insights into the role of DCR1, DCR2, and AGO2. This is not supported and this introductory statement should be changed.

Line 438-448. This section should be revisited, as the paper now includes an analysis of vsiRNAs normalized to viral RNA abundance.

Line 466-467. Rephrase: “Identifying this alternative nuclease represents an important direction for future research.” > Identifying a putative alternative nuclease would represent an important direction for future research.

Textual recommendations

Line 14-17: I recommend referring to multiple small RNA silencing pathways rather than suggesting that there is a single silencing pathway that operates through multiple branches.

Line 92, 112: Diptera insects > Dipteran insects

Line 99-101: Grammar flaws. Please revise sentence structure.

Line 119: besides RNAi > besides mediating antiviral RNAi

Line 133: it should be mentioned which gene was edited.

In general, the manuscript still contains a number of language-related issues. I trust that these will be identified and corrected as the paper is moved forward.

Reviewer #3: none

**********

PLOS authors have the option to publish the peer review history of their article (what does this mean?). If published, this will include your full peer review and any attached files.

If you choose “no”, your identity will remain anonymous but your review may still be made public.

**Do you want your identity to be public for this peer review?**  For information about this choice, including consent withdrawal, please see our Privacy Policy.

Reviewer #2: No

Reviewer #3: No

[NOTE: If reviewer comments were submitted as an attachment file, they will be attached to this email and accessible via the submission site. Please log into your account, locate the manuscript record, and check for the action link "View Attachments". If this link does not appear, there are no attachment files.]

**Figure resubmission:**

**Reproducibility:**

To enhance the reproducibility of your results, we recommend that authors of applicable studies deposit laboratory protocols in protocols.io, where a protocol can be assigned its own identifier (DOI) such that it can be cited independently in the future. Additionally, PLOS ONE offers an option to publish peer-reviewed clinical study protocols. Read more information on sharing protocols at https://plos.org/protocols?utm_medium=editorial-email&utm_source=authorletters&utm_campaign=protocols

# Author comment: Author response to Decision Letter 3

**DOI:** 10.1371/journal.ppat.1014753.r006

12 Oct 2026

Attachment Submitted filename: Response to Editor and Reviewers-R3.docx

# Editor report: Decision Letter 3

**DOI:** 10.1371/journal.ppat.1014753.r007

**Authors:** Alexander E. Gorbalenya, Barry Rockx

23 Oct 2026

Dear Dr. Li,

We are pleased to inform that your manuscript, "Functional dissection of conserved RNAi pathways in antiviral immunity of several hemipteran insects", has been editorially accepted for publication at PLOS Pathogens.

Before your manuscript can be formally accepted and sent to production, you will need to complete our formatting changes, which you will receive by email within a week. Please note that your manuscript will not be scheduled for publication until you have made the required changes.

IMPORTANT NOTES

(1) Copyediting and Proofreading: The corresponding author will receive a typeset proof for review, to ensure errors have not been introduced during production. Please review the PDF proof of your manuscript carefully, as this is the last chance to correct any errors. Please note that major changes, or those which affect the scientific understanding of the work, will likely cause delays to the publication date of your manuscript.

(2) Appropriate Figure Files: Please remove all name and figure # text from your figure files. Please also take this time to check that your figures are of high resolution, which will improve the readbility of your figures and help expedite your manuscript's publication. Please note that figures must have been originally created at 300dpi or higher. Do not manually increase the resolution of your files. For instructions on how to properly obtain high quality images, please review our Figure Guidelines, with examples at: http://journals.plos.org/plospathogens/s/figures.

We encourage you to use PACE (http://pace.apexcovantage.com/), a figure-checking tool which helps authors ensure all figures meet PLOS requirements so that figures can publish in the highest quality. PACE is a digital diagnostic and conversion tool for figure files. It will provide information about any failed check(s) and, if able, will automatically convert the figure into an acceptable file that passes quality checks.

To use PACE, you must first register as a user. Then, login and navigate to the UPLOAD tab, where you will find detailed instructions on how to use the tool.

(3) Striking Image: Please upload a striking still image to accompany your article if one is available (you can include a new image or an existing one from within your manuscript). Should your paper be accepted, this image will be considered for our monthly issue image and may also appear on our website to feature your article. Please upload this as a separate file, selecting "striking image" as the file type upon upload. Please also include a separate "Other" file with a caption, including credits and any potential copyright information. Please do not include the caption in the main article file. If your image is from someone other than yourself, please ensure that the artist has read and agreed to the terms and conditions of the Creative Commons Attribution License at http://journals.plos.org/plospathogens/s/content-license. Please note that PLOS cannot publish copyrighted images.

(4) Press Release or Related Media: If you or your institution will be preparing press materials for this manuscript, please inform our press team in advance at plospathogens@plos.org as soon as possible. We ask that you contact us within one week to plan ahead of our fast Production schedule. If you need to know your paper's publication date for related media purposes, you must coordinate with our press team, and your manuscript will remain under a strict press embargo until the publication date and time.

(5) Update your Profile Information: Now that your manuscript has been provisionally accepted, please log into Editorial Manager and update your profile, if needed. Go to http://www.editorialmanager.com/ppathogens, log in, and click on the "Update My Information" link at the top of the page. Please update your user information to ensure an efficient production and billing process.

(6) LaTeX users only: Our staff will ask you to upload a TEX file in addition to the PDF before the paper can be sent to typesetting, so please carefully review our Latex Guidelines http://journals.plos.org/plospathogens/s/latex in the meantime.

Thank you again for supporting Open Access publishing; we are looking forward to publishing your work in PLOS Pathogens.

Best regards,

Barry Rockx

Academic Editor

PLOS Pathogens

Alexander Gorbalenya

Section Editor

PLOS Pathogens

Dominique Soldati-Favre

Editor-in-Chief

PLOS Pathogens

***********************************************************

# Editor report: Acceptance letter

**DOI:** 10.1371/journal.ppat.1014753.r008

**Authors:** Alexander E. Gorbalenya, Barry Rockx

Dear Prof. Li,

We are delighted to inform you that your manuscript, "Functional dissection of conserved RNAi pathways in antiviral immunity of several hemipteran insects," has been formally accepted for publication in PLOS Pathogens.

We have now passed your article onto the PLOS Production Department who will complete the rest of the pre-publication process. All authors will receive a confirmation email upon publication.

Thank you again for supporting open-access publishing; we are looking forward to publishing your work in PLOS Pathogens.

Best regards,

Dominique Soldati-Favre

Editor-in-Chief

PLOS Pathogens
